# Bladder cancer risk factors: a comprehensive umbrella review of meta-analyses

**DOI:** 10.1097/JS9.0000000000003371

**Published:** 2025-09-10

**Authors:** Desheng Zhang, Junwei Ren, Junjiang Ye, Ruicheng Wu, Jie Wang, Dengxiong Li, Yunjin Bai, Ping Han

**Affiliations:** a Department of Urology, West China Hospital, Sichuan University, Chengdu, Sichuan, China; b Institute of Urology, West China Hospital, Sichuan University, Chengdu, Sichuan, China.

**Keywords:** AMSTAR 2, bladder cancer, GRADE, meta-analysis, risk factors, umbrella review

## Abstract

**Background:**

Bladder cancer represents a significant global health challenge, characterized by poorly understood risk factors. This study aims to synthesize meta-analytical evidence, quantify risk associations, and inform prevention strategies.

**Methods:**

We conducted a comprehensive literature search in PubMed, Embase, Web of Science, and Cochrane Library up to October 2024. Meta-analysis quality was assessed using AMSTAR 2, and evidence certainty was evaluated via the GRADE approach. To explore heterogeneity and enhance interpretation, we conducted subgroup analyses for 23 exposure–outcome associations.

**Results:**

Eighty-four meta-analyses assessing 156 risk factors were included; 79 reported potential associations with bladder cancer. These covered dietary (n = 23), disease-related (n = 20), medication (n = 10), environmental and lifestyle (n = 9), occupational (n = 13), and physiological (n = 4) factors. The GRADE system rated 60 potentially associated outcomes as very low quality, 16 as low quality, and 3 as moderate quality. Moderate-certainty evidence identified ANCA-associated vasculitis (AAV) (RR = 3.84), opium consumption (RR = 4.07), and particulate matter with a diameter of 2.5 micrometers or less (PM2.5) exposure (RR = 1.07) as risk factors. Dose–response analyses revealed increased risk with processed meat (50 g/day), red meat (100 g/day), liquor or spirits (12 g/day), and with each 5 μg/m^3^ rise in PM2.5 or 10 μg/m^3^ rise in nitrogen dioxide (NO_2_). Cruciferous vegetable intake (≥412.5 g/week) was associated with reduced risk.

**Conclusion:**

This review identifies several modifiable, dose-responsive risk factors for bladder cancer and highlights areas supported by higher-certainty evidence. These findings may assist in guiding prevention efforts – such as reducing red and processed meat intake, improving air quality, and monitoring high-risk medication to help lower the burden of bladder cancer.

**HIGHLIGTHS**

- The “umbrella review” method was used to systematically integrate multiple meta-analysis results, comprehensively assessing six major bladder cancer risk factors: dietary, disease-related, medication-related, environmental and lifestyle, occupational, and physiological, and rigorously evaluating the quality of evidence through the AMSTAR and GRADE systems.
- Dose–response analyses showed increased risk with processed meat (50 g/day), red meat (100 g/day), liquor or spirits (12 g/day), and with each 5 μg/m³ rise in PM2.5 or 10 μg/m³ rise in NO_2_. In contrast, cruciferous vegetable intake (≥412.5 g/week) was associated with reduced risk.
- Processed meat, dietary glycemic index, Western diet, Lynch syndrome, benign prostatic hyperplasia, ANCA-associated vasculitis, opium consumption, pioglitazone, angiotensin receptor blockers, PM2.5, NO_2_, painters, and tooth loss are considered potential risk factors for bladder cancer.
- Cruciferous vegetable intake of ≥412.5 g/week, fish, fruit and vegetables, aspirin, metformin, lifestyle factors, physical activity, broccoli consumption, 5-alpha reductase inhibitors, and parity are considered protective factors against bladder cancer risk.

## Introduction

Bladder cancer constitutes a significant global health challenge, ranking among the most prevalent malignant tumors of the urinary system. In 2020, it accounted for approximately 573 000 new cases worldwide and resulted in around 213 000 deaths, thereby exerting a considerable impact on public health^[1]^. Projections from Cancer Statistics 2024 indicate an anticipated 84 870 new cases and 17 420 related fatalities in the United States alone^[2]^. Notably, bladder cancer is characterized by a high recurrence rate and necessitates lifelong monitoring, positioning it as one of the most financially burdensome cancers on a per-patient basis. While early detection and intervention can markedly enhance survival rates, the persistent high recurrence rate continues to pose substantial challenges in its prevention and management. Consequently, evidence-based research into the risk factors of bladder cancer underpins effective prevention strategies and early interventions, aiming to mitigate the incidence of this disease at its origin.

Despite the conduct of several meta-analyses aimed at identifying the risk factors for bladder cancer, such as the consumption of red and processed meats^[3]^, opium use^[4]^, air pollution^[5]^, and smoking^[6]^, the findings remain inconsistently reported. Moreover, the prevailing literature tends to enumerate these risk factors without delving into their biological mechanisms or systematically evaluating the quality of the evidence^[7]^. While certain systematic reviews have proposed mechanisms, they rely on outdated data and fail to address the most prevalent risk factors encountered in daily life, such as medication use, diseases, and physiological conditions^[8]^. For instance, earlier research posited that whole milk might decrease the risk of bladder cancer, yet recent meta-analyses have concluded the contrary. Furthermore, a study by Nassour *et al* highlighted that individuals with Lynch Syndrome (LS) exhibit a 7.48-fold increased risk of developing bladder cancer compared to the general population^[9]^, underscoring the critical need to incorporate disease factors into risk assessments for bladder cancer. Therefore, it is imperative to synthesize research that integrates the latest meta-analytical findings, including factors like diseases, medications, and physiological conditions, thereby providing an updated, evidence-based foundation for understanding bladder cancer and devising more effective preventive measures to alleviate both patient suffering and societal burden.

Numerous meta-analyses in recent years have investigated the association between bladder cancer and various risk factors, including smoking, occupational exposure, and dietary habits. Despite these efforts, drawing definitive conclusions has proven difficult due to limitations in study design, variations in the measurement of risk factors, and inconsistent findings. These challenges undermine the reliability and consistency of the existing research. Consequently, this study aims to synthesize the latest meta-analytic findings, encompassing six domains related to bladder cancer: diseases, medication use, diet, environmental and lifestyle factors, and occupational exposure. This integrative approach is intended to yield more precise and scientifically robust evidence, thereby enhancing strategies for the prevention and early intervention of bladder cancer. This study has been conducted and reported in accordance with the Transparency In The reporting of Artificial INtelligence (TITAN) 2025 criteria for transparency in the use of AI and novel technologies in surgical research^[10]^.

## Methods

### Umbrella review approach

Umbrella reviews provide a comprehensive and high-level synthesis of evidence across multiple meta-analyses concerning a specific research topic^[11,12]^. This study employed an umbrella review methodology to systematically examine meta-analyses that assess the relationship between various risk factors and bladder cancer. These factors include lifestyle choices, environmental exposures, diseases, medication use, occupational hazards, and physiological conditions. Risk factors in these studies are typically quantified consistently and their associations with bladder cancer are presented as pooled effect sizes. To maintain the quality and consistency of our findings, this review was restricted to systematic reviews that included a meta-analysis.

Studies focusing on micro-level risk factors, such as genetic markers and enzymes, were excluded in favor of macro-level risk factors, including lifestyle, environmental exposures, and occupational factors. This review has been registered with PROSPERO, and additional details can be accessed via the following link: https://www.crd.york.ac.uk/PROSPERO/. This review was conducted and reported in accordance with the Preferred Reporting Items for Systematic Reviews and Meta-Analyses (PRISMA) 2020 guidelines^[13]^.

### Literature search

We conducted an exhaustive literature search using PubMed, the Cochrane Database of Systematic Reviews, Embase, and Web of Science, starting from their inception and continuing until 15 October 2024, to identify systematic reviews and meta-analyses concerning risk factors for bladder cancer. Our search strategy employed Medical Subject Headings (MeSH), keywords, and truncated word variants to ensure thorough coverage. We utilized the following search formula: “(bladder cancer OR bladder neoplasm) AND (systematic review OR meta-analysis),” in accordance with the Scottish Interdisciplinary Guidelines Network (SIGN) criteria for systematic review searches^[14]^. Our inclusion criteria encompassed meta-analyses of observational studies and randomized controlled trials that explored factors associated with bladder cancer. Two investigators independently screened the retrieved titles and abstracts, selecting studies for full-text review based on predefined criteria. These investigators then independently evaluated the full-texts of these studies to determine their eligibility. In instances of disagreement, a third researcher resolved the conflict. Additionally, we examined the reference lists of all included articles to identify any pertinent studies that might have been missed in the initial electronic search, as detailed in Supplementary Digital Content Table S1, available at: http://links.lww.com/JS9/E995.

### Eligibility criteria

Our review included meta-analyses of observational studies (e.g., cohort, case–control, and cross-sectional studies with dichotomous outcomes) and interventional studies (randomized controlled trials) that investigated risk factors associated with bladder cancer. Eligible meta-analyses were required to report relative risks, odds ratios, hazard ratios, or standardized mean differences, focusing on consistent exposure variables and health outcomes. We considered studies conducted in adult populations of any race, gender, geographic region, or study setting, involving risk factors such as lifestyle, environmental exposures, occupational hazards, physiological conditions, disease-related factors, or substance use. When a study reported multiple health outcomes linked to bladder cancer risk factors, data were extracted for each outcome separately. For meta-analyses assessing the same exposures and outcomes, inclusion was determined as follows: if the studies were published more than 2 years apart, the most recent meta-analysis was included; if published within 2 years of each other, the one with the largest sample size was selected; If the sample sizes were identical, preference was given to the meta-analysis with the higher AMSTAR-2 rating. Furthermore, if the most recent study lacked a dose–response analysis but an earlier study included one, both were considered for data extraction. Exclusions applied to studies not relevant to bladder cancer risk factors or incidence, studies with unextractable data, research focusing on genetic polymorphisms or enzymatic mechanisms, non-meta-analytic studies, grey literature and non-English publications, which were excluded to ensure methodological consistency and reporting quality.

### Data extraction

Data were independently extracted from each eligible meta-analysis by two researchers, focusing on the following elements: assessed risk factors, comparison modes (e.g., presence vs. absence, increase per 10 mmHg diastolic blood pressure, DBP), health outcomes evaluated, total number of eligible meta-analyses, number of meta-analyses included in the review, sample sizes (both number of cases and total participants), metrics employed in the meta-analyses (e.g., Relative Risk [RR], Odds Ratio [OR], Hazard Ratio [HR]), pooled estimates along with their 95% confidence intervals, and the number of studies included in each meta-analysis, categorized by type (T [total], C [cohort study], P [case–control study]). Additionally, the model of effect (random or fixed effects), measures of heterogeneity (I^2^ statistic and Cochran’s Q test *P*-value), and assessment of publication bias (*P*-value from Egger’s test) were extracted. For meta-analyses that conducted subgroup analyses, we also extracted the specific results of those subgroup analyses. If a meta-analysis conducted a dose–response analysis, the *P*-value for the test of nonlinearity was also retrieved. Discrepancies in data extraction between reviewers were resolved through discussion or referral to a third reviewer.

### Quality assessment of methods and evidence

The quality of the meta-analyses was appraised using the AMSTAR 2 (A Measurement Tool to Assess Systematic Reviews), a validated tool designed to evaluate the methodological rigor of systematic reviews and meta-analyses. AMSTAR 2 assesses various methodological aspects, including the comprehensiveness of the literature search, the appropriateness of the synthesis methods, and the clarity in reporting^[15]^. The quality of evidence for each health outcome was evaluated using the GRADE framework, which categorizes evidence into four levels: high, moderate, low, and very low^[16]^. Factors such as unexplained heterogeneity, imprecision, and publication bias could lead to downgrading the quality of evidence, whereas larger effect sizes or evidence of dose–response relationships might warrant upgrading the evidence quality. For each meta-analysis, recorded data included the number of studies incorporated, effect estimates with their 95% confidence intervals, heterogeneity statistics (I^2^ and Cochran’s Q test *P*-values), and publication bias assessments (Egger’s test *P*-value, applicable when the number of studies was 10 or more). Sensitivity analyses were conducted to gauge the robustness of the findings, which involved excluding individual studies where sufficient data were available. Data pertaining to reported dose–response relationships in the meta-analyses were also extracted. Any disagreements concerning the methodological or evidence quality assessments were resolved through consensus.

### Data analysis

We performed a comprehensive reanalysis of RRs, ORs, HRs, standardized mean differences, and weighted mean differences across all included meta-analyses. To accommodate varying study conditions and designs, both random-effects and fixed-effects models were applied. We also calculated the I^2^ statistic to evaluate heterogeneity among the studies, and the significance of this heterogeneity was assessed using the *P*-value from Cochran’s Q-test. Furthermore, Egger’s regression tests were conducted to investigate the presence of small-study effects. Sensitivity analyses were meticulously performed for each risk factor, contingent on data availability, to ascertain the robustness of our findings, particularly after the exclusion of individual studies^[17–19]^.

In exploring dose–response relationships, we extracted pertinent data concerning the association between various risk factors, such as dietary and environmental exposures, and the risk of bladder cancer. Independent evaluations of effect estimates were conducted for each observational study and randomized controlled trial included in our meta-analyses. In cases where reanalysis data were unavailable directly from the meta-analyses, pooled data were retrieved to assess heterogeneity and publication bias. All analyses and evidence synthesis were performed using Review Manager version 5.3, while Egger’s regression tests and additional sensitivity analyses were executed using Stata version 12.1.

## Results

### Characteristics of meta-analyses

**Figure 1.**
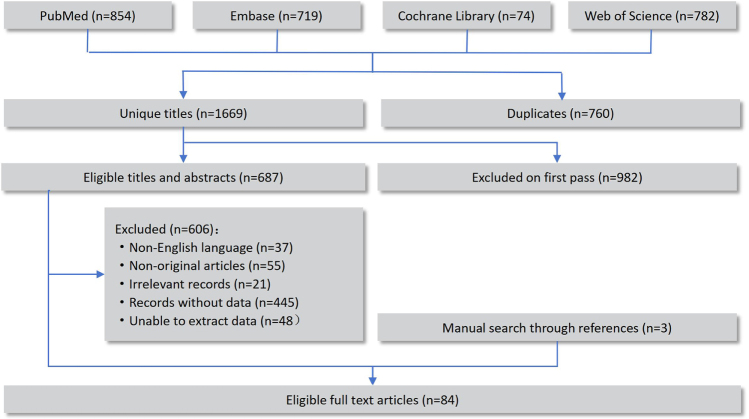
Flowchart of study selection for inclusion in the umbrella review on risk factors for bladder cancer.

**Figure 2.**
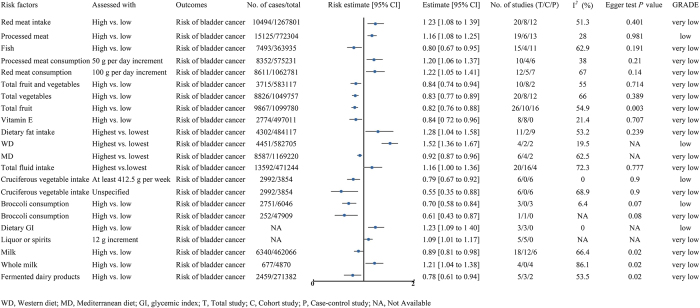
The impact of diet-related factors on bladder cancer risk.

**Figure 3.**
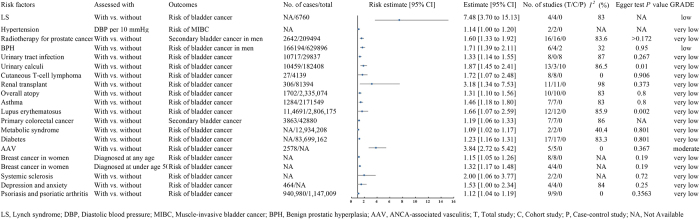
The impact of disease-related factors on bladder cancer risk.

**Figure 4.**
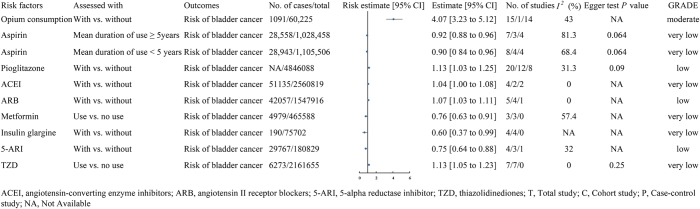
The impact of drug-related factors on bladder cancer risk.

**Figure 5.**
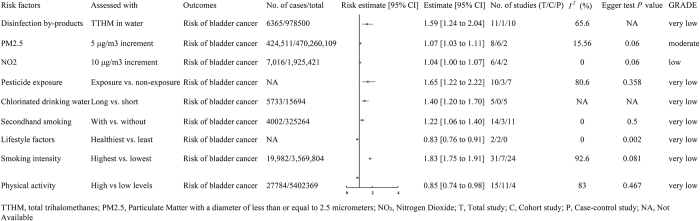
The impact of environmental and lifestyle-related factors on bladder cancer risk.

Figure 1 provides a flowchart that depicts the literature search and study selection process. We initially retrieved 2429 studies from four databases: PubMed, Cochrane Library, Embase, and Web of Science. After applying the inclusion criteria, 84 studies were selected, which encompassed meta-analytic results. We extracted meta-analytic data from these studies and generated forest plots to visually represent the putative associations identified between various factors and bladder cancer incidence. These factors were categorized as follows: diet (Fig. 2), disease (Fig. 3), drugs (Fig. 4), environment and lifestyle (Fig. 5), occupation and physical condition (Supplementary Digital Content Figures S1 and S2, available at: http://links.lww.com/JS9/E995), with further details provided in Table 1.

This figure illustrates the effects of diet-related factors (such as red meat intake, processed meat, fish, vegetables and fruits, whole grains, and dairy products) on the risk of bladder cancer. The figure includes the risk estimate and its 95% confidence interval (CI) for each risk factor, the number of cases and total sample size, heterogeneity analysis results (I^2^ value), bias assessment (Egger’s test *P*-value), and the quality of the systematic review.

This figure illustrates the effects of disease-related factors (such as Lynch syndrome, hypertension, prostate cancer radiotherapy, benign prostatic hyperplasia, urinary tract infections, urolithiasis, systemic sclerosis, lupus erythematosus, etc.) on the risk of bladder cancer. The figure includes the risk estimate and its 95% confidence interval (CI) for each disease-related factor, the number of cases and total sample size, heterogeneity analysis results (I^2^ value), bias assessment (Egger’s test *P*-value), and the quality of the systematic review.

This figure illustrates the effects of drug-related factors [such as opium intake, duration of aspirin use, pioglitazone, ACE inhibitors (ACEI), angiotensin II receptor blockers (ARB), metformin, insulin glargine, 5-ARIs, TZDs, etc.] on the risk of bladder cancer. The figure includes the risk estimate and its 95% confidence interval (CI) for each drug-related factor, the number of cases and total sample size, heterogeneity analysis results (I^2^ value), bias assessment (Egger’s test *P*-value), and the quality of the systematic review.

This figure illustrates the effects of environmental and lifestyle-related factors such as exposure to drinking water disinfection byproducts, air pollution (increased PM2.5 and NO_2_ levels), pesticide exposure, long-term consumption of chlorinated drinking water, smoking intensity, secondhand smoke exposure, and lifestyle factors (healthy vs. unhealthy) on the risk of bladder cancer. The figure includes the risk estimate and its 95% confidence interval (CI) for each risk factor, the number of cases and total sample size, heterogeneity analysis results (I^2^ value), bias assessment (Egger’s test *P*-value), and the quality of the systematic review.

**Table 1:** data extraction on bladder cancer risk factors

Risk factors	Assessed with	Outcomes	Total eligible MA	Included MA	Sample size case/total	MA metric	Estimates [95%CI]	No. of studies T/C/P	Effects model	I^2^; Q test *P* value	Egger test *P* value	AMSTAR-2	GRADE
Diet-related factors													
Significant													
Red meat intake	High vs. low	Risk of bladder cancer	2	Yu 2023	10 494/1 267 801	RR	1.23 [1.08–1.39]	20/8/12	Random	51.3%;0.004	0.401	Moderate	Very low
Processed meat	High vs. low	Risk of bladder cancer	2	Yu 2023	15 125/772 304	RR	1.16 [1.08–1.25]	19/6/13	Random	28.00%;0.125	0.981	Moderate	Low
Fish	High vs. low	Risk of bladder cancer	2	Yu 2023	7493/363 935	RR	0.80 [0.67–0.95]	15/4/11	Random	62.9%;0.001	0.191	Moderate	Very low
Processed meat consumption	50 g per day increment	Risk of bladder cancer	2	Crippa 2018	8352/575 231	RR	1.20 [1.06–1.37]	10/4/6	Random	38%;0.07	0.21	Critically low	Very low
Red meat consumption	100 g per day increment	Risk of bladder cancer	2	Crippa 2018	8611/1 062 781	RR	1.22[1.05–1.41]	12/5/7	Random	67%; <0.01	0.14	Critically low	Very low
Total fruit and vegetables	High vs. low	Risk of bladder cancer	1	Yao 2014	3715/583 117	RR	0.84 [0.74–0.94]	10/8/2	Random	55%;0.014	0.714	Critically low	Very low
Total vegetables	High vs. low	Risk of bladder cancer	1	Yao 2014	8826/1 049 757	RR	0.83 [0.77–0.89]	20/8/12	Random	66%;0.00	0.389	Critically low	Very low
Total fruit	High vs. low	Risk of bladder cancer	1	Yao 2014	9867/1 099 780	RR	0.82 [0.76–0.88]	26/10/16	Random	54.9%;0.00	0.003	Critically low	Very low
Vitamin E	High vs. low	Risk of bladder cancer	2	Lin 2019	2774/497 011	RR	0.84 [0 72–0.96]	8/8/0	Random	21.4%;0.259	0.707	Critically low	Very low
Dietary fat intake	Highest vs. lowest	Risk of bladder cancer	1	Wang 2019	4302/484 117	RR	1.28 [1.04–1.58]	11/2/9	Random	53.2%;0.019	0.239	Critically low	Very low
WD	Highest vs. lowest	Risk of bladder cancer	1	Dianatinasab 2022	4451/582 705	RR	1.52 [1.36–1.67]	4/2/2	Random	19.5%;0.293	NA	Low	Low
MD	Highest vs. lowest	Risk of bladder cancer	1	Dianatinasab 2022	8587/1 169 220	RR	0.92 [0.87–0.96]	6/4/2	Random	62.5%;0.021	NA	Low	Very low
Total fluid intake	Highest vs. lowest	Risk of bladder cancer	2	Hong 2018	13 592/471 244	RR	1.16 [1.00–1.36]	20/18/2	Random	72.3; <0.001	0.777	Critically low	Very low
Cruciferous vegetable intake	At least 412.5 g per week	Risk of bladder cancer	1	Zheng 2024	2992/3854	OR	0.79 [0.67–0.92]	6/0/6	Random	0.00%;0.600	0.90	Critically low	Low
Cruciferous vegetable intake	Unspecified	Risk of bladder cancer	1	Zheng 2024	2992/3854	OR	0.55 [0.35–0.88]	6/0/6	Random	68.9%;0.73	0.90	Critically low	Very low
Broccoli consumption	High vs. low	Risk of bladder cancer	1	Baladia 2024	2751/6046	OR	0.698 [0.578–0.844]	3/0/3	Random	6.4%;0.344	0.07	Moderate	Low
Broccoli consumption	High vs. low	Risk of bladder cancer	1	Baladia 2024	252/47 909	RR	0.610 [0.428–0.870]	1/1/0	Random	NA; NA	0.08	Moderate	Very low
Dietary GI	Highest vs. lowest	Risk of bladder cancer	1	Long 2022	NA	RR	1.23 [1.09–1.40]	3/3/0	Random	0.0%;0.001	NA	Moderate	Low
Liquor or spirits	12 g increment	Risk of bladder cancer	3	Lao 2021	NA	RR	1.09 [1.01–1.17]	5/5/0	Fixed	NA;0.12	NA	High	Very low
Milk	High vs. low	Risk of bladder cancer	1	Bermej 2019	6340/462 066	RR	0.89 [0.81–0.98]	18/12/6	Random	66.4%;0.000	0.020	Moderate	Very low
Whole milk	High vs. low	Risk of bladder cancer	1	Bermej 2019	677/4870	RR	1.21 [1.04–1.38]	4/0/4	Random	86.1%;0.000	0.020	Moderate	Very low
Fermented dairy products	High vs. low	Risk of bladder cancer	1	Bermej 2019	2459/271 382	RR	0.78 [0.61–0.94]	5/3/2	Random	53.5%;0.072	0.020	Moderate	Very low
Non-significant													
Egg	Egg consumption	Risk of bladder cancer	1	Li 2013	2715/184 727	RR	1.11 [0.90–1.35]	13/4/9	Random	63.3%;0.001	0.549	Critically low	Very low
Coffee consumption	125 ml /d increment	Risk of bladder cancer	3	Dai 2019	11 754/2 106 261	RR	1.01 [0.98–1.03]	15/15/0	Random	56.3%;0.65	>0.40	Critically low	Very low
Total carotenoid intake	Highest compared with lowest categories	Risk of bladder cancer	1	Wu 2020	6078/521 015	RR	0.88 [0.76–1.03]	11/4/7	Random	74.8%; <0.0001	0.07	Moderate	Very low
Circulating carotenoid concentrations	Highest with the lowest	Risk of bladder cancer	1	Wu 2020	1051/1546	RR	0.36 [0.12–1.07]	3/0/3	Random	85.2%;0.001	0.47	Moderate	Very low
White meat	High vs. low	Risk of bladder cancer	2	Yu 2023	8905/758 212	RR	0.96 [0.83–1.10]	15/6/9	Random	53.7%;0.007	0.078	Moderate	Very low
Total tea consumption	Highest vs. lowest	Risk of bladder cancer	2	Weng 2017	17 450/491 321	RR	0.96 [0.86–1.06]	32/7/25	Random	54.2%;0.000	0.43	Low	Very low
Black tea consumption	High vs. low	Risk of bladder cancer	2	Weng 2017	3116/62 008	RR	0.84 [0.70–1.01]	10/3/7	Random	34.9%;0.129	0.76	Low	Very low
Green tea consumption	High vs. low	Risk of bladder cancer	2	Weng 2017	2199/503 110	RR	0.95 [0.73–1.24]	7/3/4	Random	52.5%;0.049	0.06	Low	Very low
Dietary inflammatory potential	Highest vs. lowest	Risk of bladder cancer	1	Dai 2024	3633/373 010	RR	1.22[0.97–1.54]	6/3/3	Random	67%;0.01	<0.001	Moderate	Very low
DII	Highest vs. lowest	Risk of bladder cancer	1	Dianatinasab 2022	2167/258 078	RR	1.04 [0.94–1.13]	4/2/2	Random	61.4% 0.051	NA	Low	Very low
Soft drinks	Highest vs. lowest	Risk of bladder cancer	1	Boyle 2014	NA	RR	1.13 [0.89–1.45]	5/5/0	Random	0.00%; NA	0.38	Critically low	Very low
Alcohol consumption	Light vs. none	Risk of bladder cancer	3	Lao 2021	1928/488 213	RR	0.96 [0.84–1.08]	2/2/0	Random	0%;0.805	NA	High	Very low
Alcohol consumption	Moderate vs. none	Risk of bladder cancer	3	Lao 2021	1928/488 213	RR	0.90 [0.53–1.51]	2/2/0	Random	55.3%;0.135	NA	High	Very low
Alcohol consumption	Heavy vs. none	Risk of bladder cancer	3	Lao 2021	1928/488 213	RR	0.97 [0.69–1.37]	2/2/0	Random	48.0%;0.166	NA	High	Very low
Beer	12 g increment	Risk of bladder cancer	3	Lao 2021	NA	RR	1.03 [0.98–1.08]	5/5/0	Fixed	NA;0.29	NA	High	Very low
Wine	12 g increment	Risk of bladder cancer	3	Lao 2021	NA	RR	1.01 [0.97–1.05]	4/4/0	Fixed	NA;0.79	NA	High	Very low
Vitamin A	High dose vs. low dose	Risk of bladder cancer	2	Park 2017	NA	RR	0.86 [0.65–1.13]	5RCT	Random	61.7%; NA	0.393	Low	Low
Vitamin B6	High dose vs. low dose	Risk of bladder cancer	2	Park 2017	NA	RR	0.77 [0.49–1.20]	3 RCT	Random	78.8%;NA	0.393	Low	Low
Vitamin C	High dose vs. low dose	Risk of bladder cancer	2	Park 2017	NA	RR	0.74 [0.36–1.54]	2 RCT	Random	88.8%; NA	0.393	Low	Low
Vitamin D	High dose vs. low dose	Risk of bladder cancer	2	Park 2017	NA	RR	1.05 [0.85–1.29]	1 RCT	NA	NA	0.393	Low	Low
Beta-carotene	High dose vs. low dose	Risk of bladder cancer	2	Park 2017	NA	RR	1.19 [0.96–1.46]	6 RCT	Fixed	0%; NA	0.393	Low	Moderate
Nitrate	High dose vs. low dose	Risk of bladder cancer	1	Seyyedsalehi 2021	6145/715 657	OR	1.01 [0.76–1.33]	10/6/4	Random	87.2%;0.000	0.52	Low	Very low
Nitrate	Moderate dose vs. low dose	Risk of bladder cancer	1	Seyyedsalehi 2021	7042/1 193 628	OR	1.18 [0.87–1.59]	12/4/8	Random	94.4%;0.000	0.52	Low	Very low
Nitrite	High dose vs. low dose	Risk of bladder cancer	1	Seyyedsalehi 2021	3418/421 648	OR	0.91 [0.75–1.11]	6/5/1	Random	61.1%;0.025	0.52	Low	Very low
Nitrite	Moderate dose vs. low dose	Risk of bladder cancer	1	Seyyedsalehi 2021	4272/722 581	OR	1.42 [0.97–2.09]	7/5/2	Random	95.0%;0.000	0.52	Low	Very low
Sugar-sweetened beverages	Highest vs. lowest	Risk of bladder cancer	1	Li 2021	3039/239 587	RR	1.14 [0.98–1.33]	6/1/5	Random	0.00%;0.095	0.005	Moderate	Very low
Potato consumption	High and low intake	Risk of bladder cancer	1	Mofrad 2021	2295/5023	RR	0.72 [0.46–1.14]	5/0/5	Random	NA	NA	Moderate	Very low
Total dairy products	High vs. low	Risk of bladder cancer	1	Bermej 2019	3394/207 706	RR	0.92 [0.72–1.13]	6/2/4	Random	83.4%;0.000	0.020	Moderate	Very low
Cheese	High vs. low	Risk of bladder cancer	1	Bermej 2019	2879/274 291	RR	0.93 [0.79–1.07]	7/3/4	Random	72.7%;0.001	0.020	Moderate	Very low
Butter	High vs. low	Risk of bladder cancer	1	Bermej 2019	2196/191 031	RR	1.00 [0.95–1.06]	5/2/3	Random	6.0%;0.372	0.020	Moderate	Very low
Total meat	High vs. low	Risk of bladder cancer	2	Yu 2023	5425/137 163	RR	1.10 [0.92–1.31]	11/2/9	Random	55.2%;0.014	0.821	Moderate	Very low
Vitamin E	High vs. low	Risk of bladder cancer	2	Lin 2019	491/78 590	RR	1.10 [0.86–1.35]	3 RCT	Random	0.0%;0.963	0.707	Critically low	Low
Cruciferous vegetable intake	Unspecified	Risk of bladder cancer	1	Zheng 2024	NA/1 463 894	OR	1.00 [0.88–1.13]	10/10/0	Random	0.0%;1.000	0.015	Critically low	Very low
Cruciferous vegetable intake	>262.5 g/week	Risk of bladder cancer	1	Zheng 2024	NA/1 463 894	OR	0.91 [0.77 to1.07]	10/10/0	Random	0.0%;0.585	0.015	Critically low	Very low
Obesity (BMI = >30 kg/m^2^)	With vs. without	Risk of bladder cancer	1	Ahmadinezhad 2022	NA/29 563 710	RR	1.03 [0.96–1.12]	23/23/0	Random	80.2%;0.000	0.684	Critically low	Very low
Disease-related factors													
Significant													
LS	With vs. without	Relative risk of bladder cancer	1	Nassour 2023	NA/6760	RR	7.48 [3.70–15.13]	4/4/0	Random	83%; <0.01	NA	Critically low	Low
Hypertension	DBP per 10 mmHg	Risk of MIBC	1	Connaughton 2022	NA	OR	1.14 [1.00–1.20]	2/2/0	Random	NA	NA	Critically low	Very low
Radiotherapy for prostate cancer	With vs. without	Secondary bladder cancer in men	1	Zhao 2019	2642/209 494	HR	1.60 [1.33–1.92]	16/16/0	Random	83.6%, *P* = 0.000	>0.172	High	Very low
BPH	With vs. without	Risk of bladder cancer in men	1	Dai 2016	166 194/629 896	RR	1.71 [1.39–2.11]	6/4/2	Random	32%;0.19	0.95	Low	Low
Urinary tract infection	With vs. without	Risk of bladder cancer	1	Bayne 2018	10 717/29 837	RR	1.33 [1.14–1.55]	8/0/8	Random	87%; *P* = 0.000	0.267	Moderate	Very low
Urinary calculi	With vs. without	Risk of bladder cancer	1	Yu 2018	10 459/182 408	OR	1.87 [1.45–2.41]	13/3/10	Random	86.5%; NA	0.01	Critically low	Very low
Cutaneous T-cell lymphoma	With vs. without	Risk of bladder cancer	1	Goyal 2021	27/4139	OR	1.72 [1.07–2.48]	8/8/0	Random	0%;0.73	0.906	Moderate	Very low
Renal transplant	With vs. without	Risk of bladder cancer	1	Yan 2014	306/81 394	RR	3.18 [1.34–7.53]	11/11/0	Random	98.0%; *P* = 0.000	0.373	Critically low	Very low
Overall atopy	With vs. without	Risk of bladder cancer	1	Feng 2021	1702/2 335 074	RR	1.31 [1.10–1.56]	10/10/0	Random	83.0%; *P* < 0.01	0.80	Low	Very low
Asthma	With vs. without	Risk of bladder cancer	1	Feng 2021	1284/2 171 549	RR	1.46 [1.18–1.80]	7/7/0	Random	83.0%; *P* < 0.01	0.80	Low	Very low
Lupus erythematosus	With vs. without	Risk of bladder cancer	1	Zhang 2022	11 4691/2 806 175	RR	1.66 [1.07–2.59]	12/12/0	Random	85.9%; *P* <0.001	0.002	Low	Very low
Primary colorectal cancer	With vs. without	Secondary bladder cancer	1	Robertson 2022	3863/42 880	RR	1.19 [1.06–1.33]	7/7/0	Random	86%; *P* = 0.02	NA	Moderate	Very low
Metabolic syndrome	With vs. without	Risk of bladder cancer	1	Ahmadinezhad 2022	NA/12 934 208	RR	1.09 [1.02–1.17]	2/2/0	Random	40.4%;0.152	0.801	Critically low	Very low
Diabetes	With vs. without	Risk of bladder cancer	1	Ahmadinezhad 2022	NA/83 699 162	RR	1.23 [1.16–1.31]	17/17/0	Random	83.3%; *P* = 0.000	0.801	Critically low	Very low
AVV	With vs. without	Risk of bladder cancer	1	Shang 2015	2578/NA	RR	3.84 [2.72–5.42]	5/5/0	Fixed	0%;0.809	0.367	Low	Moderate
Breast cancer in women	Breast cancer diagnosed at any age	Risk of bladder cancer	1	Allen 2023	NA	RR	1.15 [1.05–1.26]	8/8/0	Random	NA	0.19	Low	Very low
Breast cancer in women	Breast cancer diagnosed at underage 50	Risk of bladder cancer	1	Allen 2023	NA	RR	1.32 [1.17–1.48]	4/4/0	Random	NA	0.19	Low	Very low
Systemic sclerosis	With vs. without	Risk of bladder cancer	1	Onishi 2013	NA	RR	2.00 [1.06–3.77]	2/2/0	Random	NA	0.72	Moderate	Very low
Depression and anxiety	With vs. without	Risk of bladder cancer	1	Wang 2020	464/NA	RR	1.53 [1.00–2.34]	4/4/0	Random	84.0%; <0.001	0.25	Moderate	Very low
Psoriasis and psoriatic arthritis	With vs. without	Risk of bladder cancer	1	Vaengebjerg 2020	940 980/1 147 009	RR	1.12 [1.04–1.19]	9/9/0	Random	0%;0.002	0.3563	Moderate	Very low
Non-significant													
Ankylosing spondylitis	With vs. without	Risk of bladder cancer	1	Yu 2024	257/58 503	RR	1.21 [0.92–1.60]	5/5/0	Random	50.4%;0.089	0.813	Moderate	Very low
Inflammatory bowel	With vs. without	Risk of bladder cancer	1	Zhang 2021	442 603/533 167	RR	0.99 [0.87–1.12]	11/9/2	Random	0.0%;0.441	0.194	Moderate	Very low
Hepatitis C virus infection	With vs. without	Risk of bladder cancer	1	Ma 2021	NA	RR	0.92[0.82–1.03]	5/3/2	Random	19.2%;0.293	0.536	Low	Very low
Periodontal disease	With vs. without	Risk of bladder cancer	1	Li 2022	NA	RR	1.19 [0.95–1.49]	3/3/0	Random	0%;0.664	0.473	High	Very low
Rhinitis	With vs. without	Risk of bladder cancer	1	Feng 2021	234/1 177 243	RR	1.03 [0.74–1.44]	4/4/0	Random	63%;0.04	0.80	Low	Very low
Breast cancer in women	Breast cancer diagnosed at age 50 or over	Risk of bladder cancer	1	Allen 2023	NA	RR	1.08 [0.89–1.30]	4/4/0	Random	NA	0.19	Low	Very low
Drug-related factors													
Significant													
Opium consumption	With vs. without	Risk of bladder cancer	1	Filho 2023	1091/60 225	RR	4.07 [3.23–5.12]	15/1/14	Random	43%;0.09	NA	High	Moderate
Aspirin	Mean duration of use ≥5 years	Risk of bladder cancer	1	Fan 2021	28 558/1 028 458	RR	0.92 [0.88–0.96]	7/3/4	Random	81.3%;0.00	0.064	High	Very low
Aspirin	Mean duration of use <5 years	Risk of bladder cancer	1	Fan 2021	28 943/1 105 506	RR	0.90 [0 84–0.96]	8/4/4	Random	68.4%;0.01	0.064	High	Very low
Pioglitazone	With vs. without	Risk of bladder cancer	1	Tang 2018	NA/4 846 088	OR	1.13 [1.03–1.25]	20/12/8	Random	31.3%;0.095	0.09	Low	Low
ACEI	With vs. without	Risk of bladder cancer	1	Xie 2020	51 135/2 560 819	RR	1.04 [1.00–1.08]	4/2/2	Random	0.00%;0.562	NA	Low	Very low
ARB	With vs. without	Risk of bladder cancer	1	Xie 2020	42 057/1 547 916	RR	1.07 [1.03–1. 11]	5/4/1	Random	0.00%;0.515	NA	Low	Low
Metformin	With vs. without	Risk of bladder cancer	1	Zhang 2021	4979/465 588	OR	0.76 [0.63–0.91]	3/3/0	Random	57.4%;0.070	NA	Critically low	Very low
Insulin glargine	With vs. without	Risk of bladder cancer	1	Tang 2012	190/75 702	OR	0.6 [0.37–0.99]	4/4/0	Random	NA	NA	Moderate	Very low
5-ARI	With vs. without	Risk of bladder cancer	1	Xiang 2021	29 767/180 829	OR	0.75 [0.64–0.88]	4/3/1	Fixed	32%;0.22	NA	Low	Low
TZD	Use vs. no use	Risk of bladder cancer	2	BOSETTI 2013	6273/2 161 655	RR	1.13 [1.05–1.23]	7/7/0	Random	0.00%;0.690	0.25	Critically low	Very low
Non-significant													
Pioglitazone	With vs. without	Risk of bladder cancer	1	Tang 2018	NA/9114	OR	1.84 [0.99–3.42]	2 RCT	Random	0.00%;0.511	0.09	Low	Moderate
Acetaminophen	Regular/any use	Risk of bladder cancer	1	Zhang 2013	6733/97 108	RR	1.01 [0.88–1.17]	10/2/8	Random	7.3%;0.375	0.23	Critically low	Very low
Non-aspirin NSAIDs	Regular/any use	Risk of bladder cancer	1	Zhang 2013	5663/761 678	RR	0.87 [0.73–1.05]	6/3/3	Random	79.3%;0.000	0.118	Critically low	Very low
CCB	With vs. without	Risk of bladder cancer	1	Xie 2020	45 299/1 097 423	RR	1.16 [0.91–1.47]	6/2/4	Random	83.2%;0.00	NA	Low	Very low
Diuretics	With vs. without	Risk of bladder cancer	1	Xie 2020	33 752/67 504	RR	1.16 [0.93–1.46]	2/0/2	Random	85.8%;0.001	NA	Low	Very low
Estrogen-progestogen therapy for hormone replacement therapy	With vs. without	Risk of bladder cancer	1	Xu 2020	NA	RR	0.79 [0.59–1.06]	5/5/0	Random	55.1%;0.063	NA	Critically low	Very low
Sodium-glucose transporter-2 inhibitors	With vs. without	Risk of bladder cancer	1	Spiazzi 2024	245/87 218	RR	0.93 [0.71–1.21]	6 RCT	Random	0%;NA	0.61	High	Moderate
Statins	Statins vs. placebos	Risk of bladder cancer	1	Symvoulidis 2023	215/27 849	RR	0.89 [0.68–1.16]	4 RCT	Fixed	0%;0.72	NA	High	Moderate
Statins	Statins vs. controls	Risk of bladder cancer	1	Symvoulidis 2023	3526/1 166 668	RR	1.32 [0.76–2.30]	6 RCT	Fixed	98%; <0.00001	NA	High	Low
Spironolactone	With vs. without	Risk of bladder cancer	1	Bommareddy 2022	NA/254 392	RR	0.89 [0.74–1.06]	3/1/2	Random	85%;0.001	NA	Low	Very low
ADT	Lack of AST	Risk of bladder cancer	1	Xiang 2021	71 764/242 845	OR	1.00 [0.46–2.15]	4/4/0	Random	94%; <0.00001	NA	Low	Very low
Androgen suppression therapy: total	Lack of AST	Risk of bladder cancer	1	Xiang 2021	101 531/423 674	OR	0.92 [0.68–1.24]	8/7/1	Random	90%; <0.00001	NA	Low	Very low
Thiazolidinediones: rosiglitazone	Use vs. no use	Risk of bladder cancer	2	BOSETTI 2013	3975/1 508 042	RR	1.08 [0.95–1.23]	3/3/0	Random	0%;0.941	0.25	Critically low	Very low
Thiazolidinediones: duration of pioglitazone	Duration of pioglitazone	Risk of bladder cancer	2	BOSETTI 2013	NA	RR	1.22 [0.93–1.59]	11/9/2	Random	82.3%;0.004	0.25	Critically low	Very low
Environmental and lifestyle-related factors													
Significant													
Disinfection by-products	Exposure vs. non-exposure	Risk of bladder cancer	1	Shi 2024	6365/978 500	RR	1.59 [1.24–2.04]	11/1/10	Random	65.6%; *P* = 0.00	NA	Moderate	Very low
PM2.5	5 μg/m^3^ increment	Risk of bladder cancer	1	Li 2024	424 511/470 260 109	RR	1.07[1.03–1.11]	8/6/2	Random	15.56%; *P* = 0.22	0.06	Moderate	Moderate
NO_2_	10 μg/m^3^ increment	Risk of bladder cancer	1	Li 2024	7016/1 925 421	RR	1.04 [1.00–1.07]	6/4/2	Random	0.00%; *P* = 0.45	0.06	Moderate	Low
Pesticide exposure	Pesticide exposure vs. non-pesticide exposure	Risk of bladder cancer	1	Liang 2016	NA	OR	1.65 [1.22–2.22]	10/3/7	Random	80.6%;0.000	0.358	Low	Very low
Chlorinated drinking water	Long vs. short	Risk of bladder cancer	1	Villanueva 2003	5733/15 694	OR	1.4 [1.2–1.7]	5/0/5	Fixed	NA;0.549	NA	Critically low	Very low
Secondhand smoking	With vs. without	Risk of bladder cancer	2	Yan 2018	4002/325 264	RR	1.22 [1.06–1.40]	14/3/11	Random	0.0%;0.691	0.50	Low	Very low
Lifestyle factors	Healthiest vs. least	Risk of bladder cancer	1	Zhang 2020	NA	HR	0.83 [0.76–0.91]	2/2/0	Random	0.00%; *P* = 050	0.002	Low	Very low
Smoking intensity	Highest vs. lowest	Risk of bladder cancer	2	Zhao 2022	19 982/3 569 804	RR	1.83 [1.75–1.91]	31/7/24	Random	92.6%;0.000	0.081	Moderate	Very low
Physical activity	High vs. low levels	Risk of bladder cancer	1	Keimling 2014	27 784/5 402 369	RR	0.85 [0.74–0.98]	15/11/4	Random	83%; <0.001	0.467	Critically low	Very low
Non-significant													
Chloroform	Exposure vs. non-exposure	Risk of bladder cancer	1	Shi 2024	857/89 434	RR	1.00 [0.99–1.02]	3/1/2	Random	66.8%;0.049	NA	Moderate	Very low
Nitrate	Exposure vs. non-exposure	Risk of bladder cancer	1	Arafa 2022	2457/8679	OR	1.00 [0.69–1.45]	3/1/2	Random	65.04%;0.057	NA	Low	Very low
Contaminated drinking water	Veterans vs. general population	Risk of bladder cancer	1	Kronstedt 2024	NA/370 408	HR	1.25 [0.97–1.61]	2/2/0	Fixed	37%;0.21	NA	Critically low	Very low
Occupational factors													
Significant													
Tetrachloroethylene	Exposure vs. non-exposure	Risk of bladder cancer	1	Vlaanderen 2014	139/NA	RR	1.47 [1.16–1.85]	8/3/5	Random	0%;0.979	0.013	Low	Very low
Painters	Painters vs. non-painters	Risk of bladder cancer	1	Guha 2010	NA	RR	1.25 [1.16–1.34]	41/11/30	Random	23.5%;0.093	NA	Critically low	Low
Rubber-manufacturing industry	Exposure vs. non-exposure	Risk of bladder cancer	1	Boniol 2017	NA/367 994	RR	1.36 [1.18–1.57]	54/35/19	Random	57%; <0.01	0.169	Critically low	Very low
Hairdressers	Hairdressers vs. non-hairdressers	Risk of bladder cancer	1	Harling 2010	530/136 301	RR	1.30 [1.14–1.47]	18/8/10	Fixed	NA;0.79	NA	Moderate	Very low
Hairdressers	Job held ≥10 years	Risk of bladder cancer	1	Harling 2010	57/15 768	RR	1.70 [1.01–2.88]	6/0/6	Fixed	NA;0.46	NA	Moderate	Very low
Male career firefighters	Firefighters vs. non-firefighters	Risk of bladder cancer	1	DeBono 2023	NA	RR	1.16 [1.08–1.26]	10/10/0	Random	0%;0.71	NA	Low	Very low
Diesel exhaust exposure	Heavy vs. non-Heavy	Risk of bladder cancer	1	Boffetta 2001	NA	RR	1.37 [1.05–1.81]	2/1/1	Random	NA;0.6	NA	Critically low	Very low
Diesel exhaust exposure	Truck drivers vs. non-truck drivers	Risk of bladder cancer	1	Boffetta 2001	NA	RR	1.17 [1.06–1.29]	11/0/11	Random	NA;0.3	NA	Critically low	Very low
Diesel exhaust exposure	Bus drivers vs. non-bus drivers	Risk of bladder cancer	1	Boffetta 2001	NA	RR	1.33 [1.22–1.45]	7/1/6	Random	NA;0.4	NA	Critically low	Very low
Coal mine workers	Coal vs. non-coal workers	Risk of bladder cancer	1	Alif 2022	NA	OR	2.17 [1.32–4.02]	2/0/2	Random	NA	NA	Low	Very low
Occupational lead exposure	Exposure vs. non-exposure	Risk of bladder cancer	1	Fu 1995	NA	RR	1.41 [1.16–1.71]	5/4/1	Random	NA	NA	Critically low	Very low
AO	Veterans vs. general population	Risk of bladder cancer	1	Kronstedt 2024	NA/2 998 392	HR	1.17 [1.01–1.36]	4/4/0	Random	89%;0.00001	NA	Critically low	Very low
DU	Veterans vs. general population	Risk of bladder cancer	1	Kronstedt 2024	NA/5825	HR	2.13 [1.31–3.48]	4/4/0	Fixed	0%;0.77	NA	Critically low	Low
Non-significant													
Occupational exposure to welding fumes	Exposure vs. non-exposure	Risk of bladder cancer	1	Collatuzzo 2024	993/10 892	RR	1.26 [0.98–1.60]	5/5/0	Random	52.0%;0.052	0.343	Moderate	Very low
Benzene exposure	Exposure vs. non-exposure	Risk of bladder cancer	1	Seyyedsalehi 2024	NA	RR	1.07 [0.97–1.18]	35/30/5	Random	44.4%;0.003	0.748	Low	Very low
Occupational asbestos exposure	Exposure vs. non-exposure	Risk of bladder cancer	1	Franco 2023	NA/504 130	RR	1.04 [0.95–1.13]	28/28/0	Random	72.9%;0.000	NA	Critically low	Very low
Bitumen workers	Bitumen workers vs. non-bitumen workers	Risk of bladder cancer	1	Mundt 2018	545/598	RR	1.09 [0.93–1.27]	21/15/6	Random	47.5%;0.006	0.225	Critically low	Very low
Roofers	Roofers vs. non-roofers	Risk of bladder cancer	1	Mundt 2018	146/190	RR	1.18 [0.86–1.62]	7/5/2	Random	39.4%;0.129	NA	Critically low	Very low
Pavers	Pavers vs. on-pavers	Risk of bladder cancer	1	Mundt 2018	335/341	RR	0.97 [0.81–1.16]	11/10/1	Random	34.7%;0.121	NA	Critically low	Very low
Personal hair dye use	Any type of hair dyes compared with no use	Risk of bladder cancer	1	Turati 2014	5136/19 736	RR	0.93 [0.82–1.05]	17/2/15	Random	34.1%;0.069	0.54	Low	Very low
Personal hair dye use	Personal use of permanent hair dyes compared with no use	Risk of bladder cancer	1	Turati 2014	2694/9619	RR	0.92 [0.77–1.09]	7/1/6	Random	27.9%;0.197	0.54	Low	Very low
Coal mine workers	Exposure vs. non-exposure	Risk of bladder cancer	1	Alif 2022	NA	RR	0.79 [0.58–1.10]	4/4/0	Random	NA	NA	Low	Very low
Acrylonitrile workers	Exposure vs. non-exposure	Risk of bladder cancer	1	Collins 1998	5/118	RR	0.8 [0.3–2.2]	3/3/0	Random	NA; 0.49	NA	Critically low	Very low
Male career firefighters	Duration of employment: <10 yrs	Risk of bladder cancer	1	DeBono 2023	NA	RR	1.34 [0.78–2.28]	4/4/0	Random	8%;0.37	NA	Low	Very low
Male career firefighters	Duration of employment: 10–20 yrs	Risk of bladder cancer	1	DeBono 2023	NA	RR	1.71 [0.63–4.68]	4/4/0	Random	8%;0.37	NA	Low	Very low
Male career firefighters	Duration of employment: >20 yrs	Risk of bladder cancer	1	DeBono 2023	NA	RR	0.88 [0.55–1.44]	4/4/0	Random	8%;0.37	NA	Low	Very low
Hairdressers	Job held ≥5 years	Risk of bladder cancer	1	Harling 2010	23/47 604	RR	1.52 [0.79–2.93]	3/1/2	Fixed	NA;0.89	NA	Moderate	Very low
Physiological factors													
Significant													
ABO blood group	A vs. O	Risk of bladder cancer	1	Cui 2023	4967/1 946 008	HR	1.12 [1.01–1.23]	5/0/5	Random	20.8%; *P* = 0.282	NA	Low	Very low
Parity	Ever parity	Risk of bladder cancer	1	Bai 2017	6214/2 699 564	RR	0.76 [0.70–0.82]	13/6/7	Fixed	0.00%;0.795	NA	Low	Low
Early menopause	Menopause vs. non-menopause	Risk of bladder cancer	1	Xu 2020	NA	RR	1.22 [1.06–1.40]	7/7/0	Random	19.2%;0.238	NA	Critically low	Very low
Tooth loss	Highest vs. lowest	Risk of bladder cancer	1	Shi 2018	605/64 095	RR	1.23 [1.12–1.35]	2/2/0	Random	0.00%;0.596	0.239	Moderate	Low
Non-significant													
ABO blood group	AB vs. O	Risk of bladder cancer	1	Cui 2023	4967/1 946 008	HR	1.06 [0.95–1.19]	5/0/5	Random	0.00%; *P* = 0.315	NA	Low	Very low
ABO blood group	B vs. O	Risk of bladder cancer	1	Cui 2023	4967/1 946 008	HR	1.09 [0.97–1.23]	5/0/5	Random	14.6%; *P* = 0.321	NA	Low	Very low
Age of menarche	Menarcheal age	Risk of bladder cancer	1	Li 2022	3719/1 350 207	RR	0.96 [0 85–1.08]	12/10/2	Random	0.00%; *P* = 0.462	0.507	Moderate	Very low
Overweight (BMI = >25 kg/m^2^)	With vs. without	Risk of bladder cancer	1	Ahmadinezhad 2022	NA/33 697 415	RR	1.06 [0.98–1.15]	23/23/0	Random	65.4%;0.000	0.684	Critically low	Very low
Obesity (BMI = >30 kg/m^2^)	With vs. without	Risk of bladder cancer	1	Ahmadinezhad 2022	NA/29 563 710	RR	1.03 [0.96–1.12]	23/23/0	Random	80.2%;0.000	0.684	Critically low	Very low

WD, Western diet; MD, Mediterranean diet; GI, glycemic index; DII, dietary-inflammatory-index; BMI, body mass index; LS, Lynch syndrome; DBP, diastolic blood pressure; MIBC, muscle-invasive bladder cancer; BPH, benign prostatic hyperplasia; AAV, ANCA-associated vasculitis; NSAIDs, non-steroidal anti-inflammatory drugs; CCB, calcium channel blockers; ADT, androgen deprivation therapy; AST, androgen suppression therapy; ACEI, angiotensin-converting enzyme inhibitors; ARB, angiotensin II receptor blockers; 5-ARI, 5-alpha reductase inhibitor; TZD, thiazolidinediones; TTHM, total trihalomethanes; PM2.5, particulate matter with a diameter of less than or equal to 2.5 micrometers; NO_2_, nitrogen dioxide; AO, agent orange; DU, depleted uranium; T, total study; C, cohort study; P, case–control study; NA, not available.

A total of 79 outcomes reported potential associations, including 23 dietary risk factors, 20 disease-related risk factors, 10 drug-related risk factors, 9 environmental and lifestyle risk factors, 13 occupational risk factors, and 4 physiological risk factors (Supplementary Digital Content Figure S3, available at: http://links.lww.com/JS9/E995). All included outcomes underwent a quality assessment using the GRADE criteria: 60 were deemed very low quality, 16 low quality, 3 moderate quality. The results were presented in descending order of GRADE rating. Notably, no outcomes were classified as high quality in this review.

### Dietary risk factors

#### Evidence of low quality

A meta-analysis of 6 cohort and 13 case–control studies found a positive association between processed meat intake and bladder cancer risk (RR = 1.16, 95% CI: 1.08–1.25) (low quality)^[3]^. Another analysis of 2 cohort and 2 case–control studies indicated that a Western diet (WD) was linked to a higher risk of bladder cancer (RR = 1.52, 95% CI: 1.36–1.67) (low quality)^[20]^. A dose–response analysis involving 6 case–control studies demonstrated that increased intake of cruciferous vegetables (≥412.5 g/week) was inversely related to bladder cancer risk (RR = 0.79, 95% CI: 0.67–0.92) (low quality)^[21]^. Additionally, 3 cohort studies suggested that a higher glycemic index (GI) was associated with an elevated risk of bladder cancer (RR = 1.23, 95% CI: 1.09–1.40) (low quality)^[22]^. Lastly, a meta-analysis of three case–control studies suggested a potential inverse association between higher broccoli consumption and bladder cancer risk (RR = 0.698, 95% CI: 0.578–0.844) (low quality)^[23]^ (Fig. 2).

#### Evidence of very low quality

The main dietary risk factors for bladder cancer include red meat intake (RR = 1.23)^[3]^, high-fat diet (RR = 1.28)^[24]^, total fluid intake (RR = 1.16)^[25]^, whole milk consumption (RR = 1.21)^[26]^, a 100 g/day increase in red meat (RR = 1.22), a 50 g/day increase in processed meat (RR = 1.20)^[27]^, and a 12 g alcohol increase from spirits or liquor (RR = 1.09)^[28]^ (Fig. 2).

The main dietary protective factors against bladder cancer include fish intake (RR = 0.80)^[3]^, the Mediterranean Diet (WD) (RR = 0.92)^[20]^, cruciferous vegetables (OR = 0.55, case–control study)^[21]^, broccoli (RR = 0.61, cohort study)^[23]^, milk (RR = 0.89)^[26]^, fermented milk products (RR = 0.78)^[26]^, total fruit intake (RR = 0.82)^[29]^, total vegetable intake (RR = 0.83)^[29]^, combined fruit and vegetable intake (RR = 0.84)^[29]^, and vitamin E intake (RR = 0.84)^[30]^ (Fig. 2).

No meaningful link was observed between bladder cancer and egg consumption^[31]^, total carotenoids, circulating carotenoid concentrations^[32]^, white meat^[3]^, total tea, black tea, green tea^[33]^, dietary inflammatory potential^[34]^, the Dietary Inflammatory Index (DII)^[20]^, soft drinks^[35]^, alcohol^[28]^, nitrites, nitrates^[36]^, sugar-sweetened beverages^[37]^, potatoes^[38]^, total dairy products, cheese, butter^[26]^, total meat^[3]^, or cruciferous vegetables (unspecified, cohort study)^[21]^. Dose–response analyses showed no significant association between coffee consumption and bladder cancer risk, even with a daily increase of one cup (125 mL)^[39]^, 12 grams (1 cup) of beer or wine^[28]^, or more than 262.5 g of cruciferous vegetables per week^[21]^. In randomized controlled trials, no significant link was found between bladder cancer and the consumption of vitamin A, B_6_, C, D, β-carotene^[40]^, or E^[30]^ (Table 1).

### Disease risk factors

#### Evidence of moderate and low quality

Individuals who had AAV had a moderately increased chance of acquiring bladder cancer compared to those who did not have the condition, according to a meta-analysis that included five cohort studies (RR = 3.84, 95% CI: 2.72–5.42) (moderate)^[41]^. Another meta-analysis including four cohort studies indicated that those with LS faced a potentially increased risk of bladder cancer (RR = 7.48, 95% CI: 3.70–15.13) (low)^[9]^. Additionally, a meta-analysis comprising two case–control studies and four cohort studies showed that those with benign prostatic hyperplasia (BPH) were far more likely to develop bladder cancer (RR = 1.71, 95% CI: 1.39–2.11) (low)^[42]^ (Fig. 3).

#### Very low-quality evidence

Several conditions are potentially associated with a higher incidence of bladder cancer, including a 10 mmHg increase in DBP (OR = 1.14)^[43]^, radiotherapy for prostate cancer (OR = 1.60)^[44]^, urinary tract infections (RR = 1.33)^[45]^, urinary calculi (OR = 1.87)^[46]^, cutaneous T-cell lymphoma (OR = 1.72)^[47]^, kidney transplant (RR = 3.18)^[48]^, atopy (RR = 1.31),^[49]^, asthma (RR = 1.46)^[49]^, systemic lupus erythematosus (RR = 1.66)^[50]^, systemic sclerosis (RR = 2.00)^[51]^, psoriasis and psoriatic arthritis (RR = 1.12)^[52]^, primary colorectal cancer (RR = 1.19)^[53]^, breast cancer in women (RR = 1.15)^[54]^, breast cancer diagnosed in women younger than 50 years old (RR = 1.32)^[54]^, depression and anxiety (RR = 1.53)^[55]^, metabolic syndrome (RR = 1.09)^[56]^, and diabetes (RR = 1.23)^[56]^ (Fig. 3).

No meaningful links were identified between bladder cancer risk and the following conditions: ankylosing spondylitis^[57]^, inflammatory bowel disease^[58]^, hepatitis C virus infection^[59]^, periodontal disease^[60]^, rhinitis^[49]^, and breast cancer diagnosed in women over the age of 50^[54]^ (Table 1).

### Drug risk factors

#### Moderate and low-quality evidence

A meta-analysis involving one cohort study and 14 case–control studies revealed that opium smokers had a fourfold higher likelihood of developing bladder cancer compared to non-smokers (RR = 4.07, 95% CI: 3.23–5.12) (moderate)^[4]^. Additionally, a review of 12 cohort studies and 8 case–control studies revealed that pioglitazone use was linked to a higher likelihood of bladder cancer (OR = 1.13, 95% CI: 1.03–1.25) (low)^[61]^. Similarly, a study involving 4 cohorts and 1 case–control showed a slight rise in the risk of bladder cancer among users of angiotensin receptor blockers (ARBs) (RR = 1.07, 95% CI: 1.03–1.11) (low)^[62]^. In contrast, an analysis of three cohorts and one case–control study observed a lower likelihood of bladder cancer among users of 5-alpha reductase inhibitors (5-ARIs) (OR = 0.75, 95% CI: 0.64–0.88) (low)^[63]^ (Fig. 4).

#### Very low-quality evidence

Certain medications are associated with altered bladder cancer risk, including angiotensin-converting enzyme inhibitors (ACEIs) (RR = 1.04)^[62]^ and thiazolidinediones (TZDs) (RR = 1.13)^[64]^ as risk-enhancing drugs, while long-term aspirin use (≥5 years, RR = 0.92)^[65]^, short-term aspirin use (<5 years, RR = 0.90)^[65]^, metformin (OR = 0.76)^[66]^, and insulin glargine (OR = 0.60)^[67]^ show protective effects (Fig. 4).

No meaningful link was observed between bladder cancer and acetaminophen, non-aspirin non-steroidal anti-inflammatory drugs (NSAIDs)^[68]^, calcium channel blockers (CCBs), diuretics^[62]^, estrogen-progestin therapy in hormone replacement therapy^[69]^, spironolactone^[70]^, androgen suppression therapy (including total androgen suppression and androgen deprivation therapy, ADT)^[63]^, TZDs (rosiglitazone)^[64]^, or other drugs such as ketones, pioglitazone, and duration of pioglitazone use^[64]^. A review of randomized controlled trials found no significant links between bladder cancer risk and pioglitazone^[61]^, inhibitors of sodium-glucose cotransporters 2 (SGLT2i)^[71]^, or statins^[72]^ (Table 1).

### Environmental and lifestyle risk factors

#### Evidence of moderate and low quality

A dose–response analysis of six cohorts and two case–control studies revealed that for every 5 μg/m^3^ increase in ambient PM_2.5_ concentration, the risk of bladder cancer increased by 7% (RR = 1.07, 95% CI: 1.03–1.11) (moderate)^[5]^. Similarly, a dose–response analysis of four cohorts and two case–control studies demonstrated that for every 10 μg/m^3^ increase in NO_2_ concentration, the risk of bladder cancer increased by 4% (RR = 1.04, 95% CI: 1.00–1.07) (low)^[5]^ (Fig. 5).

#### Very low-quality evidence

Disinfection by-products (RR = 1.59)^[73]^, pesticide exposure (OR = 1.65)^[74]^, chlorinated drinking water (OR = 1.40)^[75]^, secondhand smoke (RR = 1.22)^[76]^, and highest intensity smoking (RR = 1.83)^[6]^ are associated with increased risk of bladder cancer, while high physical activity (RR = 0.85)^[77]^ and the healthiest lifestyle adherence (HR = 0.83)^[78]^ are linked to reduced risk.

A cohort study found no significant associations between exposure to chloroform^[73]^, nitrates^[79]^, or contaminated drinking water^[80]^ and bladder cancer risk (Table 1).

### Occupational risk factors

#### Low-quality evidence

A pooled analysis of 11 cohort and 30 case–control studies suggested a potential association between working as a painter and an increased risk of bladder cancer (RR = 1.25, 95% CI: 1.16–1.34) (low)^[81]^. Additionally, an analysis of four cohort studies reported that military veterans exposed to depleted uranium (DU) had a 2.13-fold increased risk of bladder cancer (RR = 2.13, 95% CI: 1.31–3.48) (low)^[80]^ (Supplementary Digital Content Figure S1, available at: http://links.lww.com/JS9/E995).

#### Very low-quality evidence

Perchloroethylene (RR = 1.47)^[82]^, rubber manufacturing (RR = 1.36)^[83]^, barbering (RR = 1.30)^[84]^, barbering ≥10 years (RR = 1.70)^[84]^, male firefighters (RR = 1.16)^[85]^, heavy diesel exhaust (RR = 1.37)^[86]^, truck driving with diesel exhaust exposure (RR = 1.17)^[86]^, general diesel exposure (RR = 1.33)^[86]^, coal mining (RR = 2.17)^[87]^, inorganic lead exposure (RR = 1.41)^[88]^, and Agent Orange exposure among veterans (RR = 1.17)^[80]^ are associated with increased risk of bladder cancer (Supplementary Digital Content Figure S1, available at: http://links.lww.com/JS9/E995).

In cohort studies, no significant associations were found between bladder cancer risk and occupational exposure to welding fumes^[89]^, benzene exposure^[90]^, asbestos exposure^[91]^, or working as asphalt workers, roofers, and paving workers^[92]^. Similarly, no associations were found for personal use of hair dyes^[93]^, coal miners (in cohort studies only)^[87]^, acrylonitrile workers^[94]^, male firefighters (categorized by years of service: <10, 10–20, >20 years)^[85]^, or hairdressers (≥5 years in the occupation)^[84]^ (Table 1).

### Physiological risk factors

#### Evidence of low quality

A combined analysis of two cohort studies revealed that the absence of teeth was linked to a 23% higher risk of bladder cancer (RR = 1.23, 95% CI: 1.12–1.35) (low)^[95]^. In addition, a separate meta-analysis of six cohort and seven case–control studies indicated that individuals who had given birth were at a lower risk of developing bladder cancer compared to those who had never been pregnant (RR = 0.76, 95% CI: 0.70–0.82) (low)^[96]^ (Supplementary Digital Content Figure S2, available at: http://links.lww.com/JS9/E995).

#### Very low-quality evidence

Blood group A (RR = 1.12)^[97]^ and early menopause (RR = 1.22)^[69]^ are associated with increased risk of bladder cancer (Supplementary Digital Content Figure S2, available at: http://links.lww.com/JS9/E995).

In cohort studies, No meaningful links were found between bladder cancer risk and the following factors: ABO blood group (AB vs. O, B vs. O)^[97]^, age at menarche^[98]^, with a BMI of 25 kg/m^2^ or higher for overweight, and 30 kg/m^2^ or greater for obesity^[56]^ (Table 1).

### Subgroup analysis

We carefully extracted all available subgroup analyses from the included studies, yielding a total of 23 distinct evaluations. These involved a wide range of exposures, including different meat intakes^[3]^ (Supplementary Digital Content Table S2, available at: http://links.lww.com/JS9/E995), coffee consumption^[39]^ (Supplementary Digital Content Table S3, available at: http://links.lww.com/JS9/E995), milk intake^[26]^ (Supplementary Digital Content Table S4, available at: http://links.lww.com/JS9/E995), total fluid intake^[25]^ (Supplementary Digital Content Table S5, available at: http://links.lww.com/JS9/E995), vitamin E intake^[30]^ (Supplementary Digital Content Table S6, available at: http://links.lww.com/JS9/E995), alcohol consumption^[28]^ (Supplementary Digital Content Table S7, available at: http://links.lww.com/JS9/E995), dietary fat intake^[24]^ (Supplementary Digital Content Table S8, available at: http://links.lww.com/JS9/E995), BPH^[42]^ (Supplementary Digital Content Table S9, available at: http://links.lww.com/JS9/E995), lupus erythematosus^[50]^ (Supplementary Digital Content Table S10, available at: http://links.lww.com/JS9/E995), radiotherapy for prostate cancer^[44]^ (Supplementary Digital Content Table S11, available at: http://links.lww.com/JS9/E995), renal transplantation^[48]^ (Supplementary Digital Content Table S12, available at: http://links.lww.com/JS9/E995), diabetes^[56]^ (Supplementary Digital Content Table S13, available at: http://links.lww.com/JS9/E995), urinary calculi^[46]^, (Supplementary Digital Content Table S14, available at: http://links.lww.com/JS9/E995), pioglitazone use^[61]^ (Supplementary Digital Content Table S15, available at: http://links.lww.com/JS9/E995), aspirin intake^[65]^ (Supplementary Digital Content Table S16, available at: http://links.lww.com/JS9/E995), ARBs^[62]^ (Supplementary Digital Content Table S17, available at: http://links.lww.com/JS9/E995), smoking intensity^[6]^ (Supplementary Digital Content Table S18, available at: http://links.lww.com/JS9/E995), hairdressers^[84]^ (Supplementary Digital Content Table S19, available at: http://links.lww.com/JS9/E995), rubber-manufacturing industry^[83]^ (Supplementary Digital Content Table S20, available at: http://links.lww.com/JS9/E995), benzene exposure^[90]^ (Supplementary Digital Content Table S21, available at: http://links.lww.com/JS9/E995), physical activity^[77]^ (Supplementary Digital Content Table S22, available at: http://links.lww.com/JS9/E995), pesticide exposure^[74]^ (Supplementary Digital Content Table S23, available at: http://links.lww.com/JS9/E995), and parity^[96]^ (Supplementary Digital Content Table S24, available at: http://links.lww.com/JS9/E995). To enhance the clarity of the results and prioritize more credible evidence, we reported subgroup analyses rated as moderate or low certainty in the main text, while those graded as very low certainty were summarized in the supplementary materials (Supplementary Digital Content Tables S2–S24, available at: http://links.lww.com/JS9/E995).

#### Different meat intakes

Subgroup analyses suggested varying degrees of association between different types of meat intake and bladder cancer risk. Total meat intake (11 studies) showed no clear association overall (RR = 1.10, 95% CI: 0.92–1.31; I^2^ = 55.2%), with similar findings across study designs and regions; however, studies published after 2008 (RR = 1.31) and those with higher quality scores (RR = 1.27) indicated a possible positive association. Red meat intake (20 studies) was potentially associated with increased risk (RR = 1.23), particularly in case–control studies (RR = 1.31), non-European populations (RR = 1.47), and post-2008 publications (RR = 1.26). Processed meat intake (19 studies) showed some evidence of positive association (RR = 1.16), with slightly stronger indications in non-European groups (RR = 1.39). White meat intake (15 studies) did not demonstrate a consistent association overall (RR = 0.96), though inverse relationships were suggested in non-European regions (RR = 0.75) and earlier studies. Fish intake (15 studies) was associated with a reduced risk (RR = 0.80), especially in populations outside Europe/USA (RR = 0.60) and lower-quality studies. Conversely, subgroup analyses found no clear association for total meat intake across any study design or region. Red meat showed no association in cohort studies, earlier publications, or lower-quality studies. Processed meat was not associated with risk in cohort studies. White meat and fish intake showed no association in cohort studies, Europe/USA regions, earlier publications, or lower-quality studies (Supplementary Digital Content Table S2, available at: http://links.lww.com/JS9/E995).

#### BPH

Subgroup analyses suggested a potential association between the exposure and bladder cancer risk across 6 studies (RR = 1.71, 95% CI: 1.39–2.11; I^2^ = 32%). This association was observed in both case–control and cohort studies, with RRs of 2.50 and 1.58, respectively. By ethnicity, increased risks were reported in Asian (RR = 1.95) and Caucasian populations (RR = 1.51). Risk estimates were higher in population-based studies (RR = 2.18) compared to hospital-based studies (RR = 1.71). Studies with higher quality (NOS score >6) also indicated a positive association (RR = 1.75, 95% CI: 1.38–2.22).

Subgroup analysis showed no association between BPH and bladder cancer in studies with a NOS score ≤6 (Supplementary Digital Content Table S9, available at: http://links.lww.com/JS9/E995).

#### Pioglitazone use

Subgroup analyses suggested a potential association between pioglitazone use and bladder cancer risk (19 studies; OR = 1.13, 95% CI: 1.03–1.25; I^2^ = 31.3%). Higher risks were observed in studies that adjusted for smoking (OR = 1.28), were conducted in Europe (OR = 1.17), or were funded by non-industry sources (OR = 1.20). Increased risks were also reported in comparisons with non-TZD users (OR = 1.62) and in cohort studies (OR = 1.12). Regarding exposure, elevated risks were found in the 10.5–28 g (OR = 1.27) and >28 g (OR = 1.66) dose groups, as well as with 1–2 years (OR = 1.25) and >2 years (OR = 1.49) of use.

Subgroup analyses showed no clear association between pioglitazone use and bladder cancer risk in studies without smoking adjustment, conducted in the United States or Asia, or funded by industry sources (e.g., Takeda). No significant associations were found in subgroups using rosiglitazone, insulin, placebo, or in never users of pioglitazone. Similarly, case–control studies, both sexes (men and women), lower cumulative doses (≤10.5 g, ≤14 g, 14–40 g, >40 g), and shorter durations of use (≤1 year, ≤1.5 years, 1.5–4 years, >4 years) did not show consistent evidence of increased risk. (Supplementary Digital Content Table S15, available at: http://links.lww.com/JS9/E995).

#### Parity

Subgroup analyses suggested an inverse association between parity and bladder cancer risk (13 studies; RR = 0.76, 95% CI: 0.70–0.82; I^2^ = 0%). Reduced risks were observed for 1–2 births (RR = 0.82), 3–4 births (RR = 0.79), and ≥5 births (RR = 0.76) compared with nulliparous women. Consistent associations were found in both cohort and case–control studies, in studies with fewer than 250 cases (RR = 0.68) and more than 250 cases (RR = 0.78), among never smokers (RR = 0.47), and in studies adjusted for smoking, age, or BMI.

No significant association between parity and bladder cancer risk was observed among ever smokers (Supplementary Digital Content Table S24, available at: http://links.lww.com/JS9/E995)

### Heterogeneity

We reevaluated 133 risk factors for heterogeneity using both random-effects and fixed-effects models. This reanalysis identified significant variability (I^2^ >50% or *P*-value <0.1 for Cochran’s Q test) in approximately 72 risk factors. Such heterogeneity in results is likely attributable to variations in study settings, geographic locations, ethnic backgrounds, gender, age, methodological quality, study design, sample sizes, duration of follow-up, and adjustments for potential confounding variables. Additionally, 23 factors were not analyzed due to a lack of sufficient data.

### Risk of bias assessment

Within this umbrella review, Egger’s test was conducted on 84 studies. The results indicated significant publication bias in 21 studies, particularly those investigating urinary tract stones (*P* = 0.01), systemic lupus erythematosus (*P* = 0.002), lifestyle factors (*P* = 0.002), total fruit consumption (*P* = 0.003), exposure to tetrachloroethylene (*P* = 0.013), intake of cruciferous vegetables (*P* = 0.015), and various dairy products including milk (*P* = 0.02), whole milk (*P* = 0.02), fermented dairy products (*P* = 0.02), and dietary inflammatory potential (*P* < 0.001), along with sugar-sweetened beverages (*P* = 0.005), and aggregate dairy products, cheese, and butter (*P* = 0.02). These findings suggest a potential over-reporting of favorable results. Moreover, 23 studies either did not perform or failed to report results from Egger’s test, thus precluding a comprehensive assessment of publication bias. For those meta-analyses that were not reanalyzed, no significant publication bias was detected, or the statistical tests for publication bias were not conducted or reported for specific exposures, which may be partially explained by the limited number of studies involved. The study generated funnel plots for outcome indicators with ≥10 studies, specifically including: red meat intake (Supplementary Digital Content Figure S4, available at: http://links.lww.com/JS9/E995), processed meat (Supplementary Digital Content Figure S5, available at: http://links.lww.com/JS9/E995), fish (Supplementary Digital Content Figure S6, available at: http://links.lww.com/JS9/E995), processed meat consumption (Supplementary Digital Content Figure S7, available at: http://links.lww.com/JS9/E995), red meat consumption (100 g per day increment) (Supplementary Digital Content Figure S8, available at: http://links.lww.com/JS9/E995), total fruit and vegetables (Supplementary Digital Content Figure S9, available at: http://links.lww.com/JS9/E995), total vegetables (Supplementary Digital Content Figure S10, available at: http://links.lww.com/JS9/E995), funnel plot of total fruit intake and bladder cancer risk (Supplementary Digital Content Figure S11, available at: http://links.lww.com/JS9/E995), dietary fat intake (Supplementary Digital Content Figure S12, available at: http://links.lww.com/JS9/E995), milk intake (Supplementary Digital Content Figure S13, available at: http://links.lww.com/JS9/E995), radiotherapy for prostate cancer (Supplementary Digital Content Figure S14, available at: http://links.lww.com/JS9/E995), urinary calculi (Supplementary Digital Content Figure S15, available at: http://links.lww.com/JS9/E995), renal transplant (Supplementary Digital Content Figure S16, available at: http://links.lww.com/JS9/E995), overall atopy (Supplementary Digital Content Figure S17, available at: http://links.lww.com/JS9/E995), diabetes (Supplementary Digital Content Figure S18, available at: http://links.lww.com/JS9/E995), opium consumption (Supplementary Digital Content Figure S19, available at: http://links.lww.com/JS9/E995), pioglitazone (Supplementary Digital Content Figure S20, available at: http://links.lww.com/JS9/E995), disinfection by-products (Supplementary Digital Content Figure S21, available at: http://links.lww.com/JS9/E995), pesticide exposure (Supplementary Digital Content Figure S22, available at: http://links.lww.com/JS9/E995), secondhand smoking (Supplementary Digital Content Figure S23, available at: http://links.lww.com/JS9/E995), smoking intensity (Supplementary Digital Content Figure S24, available at: http://links.lww.com/JS9/E995), physical activity (Supplementary Digital Content Figure S25, available at: http://links.lww.com/JS9/E995), painters (Supplementary Digital Content Figure S26, available at: http://links.lww.com/JS9/E995), parity (Supplementary Digital Content Figure S27, available at: http://links.lww.com/JS9/E995). Notably, the funnel plots for milk intake (Supplementary Digital Content Figure S13, available at: http://links.lww.com/JS9/E995), disinfection by-products exposure (Supplementary Digital Content Figure S21, available at: http://links.lww.com/JS9/E995), and parity (Supplementary Digital Content Figure S27, available at: http://links.lww.com/JS9/E995) showed visible asymmetry, suggesting potential publication bias. To further assess its impact, we conducted trim-and-fill analyses for these three exposure–outcome relationships. The results indicated that although potential unpublished studies may exist, the adjusted effect estimates remained largely unchanged, and statistical significance was not materially affected, suggesting that the original findings are relatively robust (Supplementary Digital Content Figures S28–S30, available at: http://links.lww.com/JS9/E995).

### AMSTAR 2 and GRADE classification

Across the entirety of the reviewed studies, methodological quality assessed at the study level using AMSTAR-2 showed that 7 articles were rated as high, 25 as moderate, 25 as low, and 28 as critically low. Supplementary Digital Content Table S25 (available at: http://links.lww.com/JS9/E995) summarizes the AMSTAR-2 overall ratings for each included article. With respect to the GRADE classification, approximately 21 outcomes were adjudged as “low,” 128 as “very low,” and 7 as “moderate” in quality. Despite these outcomes being meta-analyses of randomized controlled trials, they were predominantly classified as low quality due to prevalent issues such as bias, inconsistency, and imprecision. Studies that were assigned a “moderate” quality rating typically exhibited either a distinct dose–response relationship or a substantial effect size, with no significant biases identified. In contrast, the majority of the outcomes were assessed as “very low” in quality. Supplementary Digital Content Table S26 (available at: http://links.lww.com/JS9/E995) details the GRADE scores associated with each outcome.

To facilitate interpretation, we have summarized the most robust associations – those based on moderate to low GRADE certainty – highlighting their respective effect sizes, AMSTAR 2, and GRADE classifications (refer to Table 2).

**Table 2:** Main-text summary of the most robust risk and protective factors for bladder cancer

Risk factors	Assessed with	Effect size	AMSTAR-2	GRADE level
Opium consumption	With vs. without	4.07 [3.23 to 5.12]	High	Moderate
PM2.5	5 μg/m^3^ increment	1.07[1.03 to 1.11]	Moderate	Moderate
AAV	With vs. without	3.84 [2.72 to 5.42]	Moderate	Moderate
Processed meat	High vs. low	1.16 [1.08 to 1.25]	Moderate	Low
Tooth loss	Highest vs. lowest	1.23 [1.12 to 1.35]	Moderate	Low
Dietary GI	Highest vs. lowest	1.23 [1.09 to 1.40]	Moderate	Low
NO_2_	10 μg/m^3^ increment	1.04 [1.00 to 1.07]	Moderate	Low
BPH	With vs. without	1.71 [1.39 to 2.11]	Low	Low
Pioglitazone	With vs. without	1.13 [1.03 to 1.25]	Low	Low
ARB	With vs. without	1.07 [1.03 to 1. 11]	Low	Low
WD	Highest vs. lowest	1.52 [1.36 to 1.67]	Low	Low
Painters	Painters vs. non-painters	1.25 [1.16 to 1.34]	Critically low	Low
DU	Military Veterans vs. general population	2.13 [1.31 to 3.48]	Critically low	Low
LS	With vs. without	7.48 [3.70 to 15.13]	Critically low	Low
Protective factors				
Broccoli consumption	High vs. low	0.698 [0.578 to 0.844]	Moderate	Low
5-ARI	With vs. without	0.75 [0.64 to 0.88]	Low	Low
Parity	Ever parity	0.76 [0.70 to 0.82]	Low	Low
Cruciferous vegetable intake	≥412.5 g/week	0.79 [0.67 to 0.92]	Critically low	Low

PM2.5, particulate matter with a diameter of less than or equal to 2.5 micrometers; NO_2_, nitrogen dioxide; AAV, ANCA-associated vasculitis; LS, Lynch syndrome; WD, Western diet; GI, glycemic index; BPH, benign prostatic hyperplasia; ARB, angiotensin II receptor blockers; DU, depleted uranium; 5-ARI, 5-alpha reductase inhibitor.

## Discussion

### Key findings and potential reasons

This meta-analysis identified potential associations between various risk factors for bladder cancer and numerous health outcomes, utilizing diverse exposure measures such as smoking versus non-smoking, low-dose versus high-dose exposure, long-term versus short-term exposure, and incremental daily exposure versus no exposure. The criteria for inclusion were met by 84 publications, which encompassed a total of 2429 independent studies. This extensive body of literature includes findings from 79 studies that suggested potential associations across various factors, including diet, disease, medications, environmental factors, lifestyle choices, occupational hazards, and physiological conditions, all in relation to the incidence of bladder cancer.

### Dietary risk factors

Recent studies suggest a link between red and processed meat consumption and an increased risk of bladder cancer. The IARC classifies red meat as probably carcinogenic (Group 2A) and processed meat as carcinogenic (Group 1). Epidemiological data show that daily intake of 100 g of red meat increases colorectal cancer risk by 17%, while 50 g of processed meat increases risk by 18%^[99]^. This association is likely driven by carcinogenic compounds like nitrosamines, heterocyclic aromatic compounds, polycyclic hydrocarbons, and heme iron, which are produced during cooking and increase cancer risk^[100]^. Furthermore, the intestinal microbiota may also play a critical role in the pathogenesis of bladder cancer. Furthermore, the intestinal microbiota may play a key role in bladder cancer by converting nitrosamines like N-Butyl-N-(4-hydroxybutyl) nitrosamine (BBN) into more carcinogenic forms. Antibiotics that reduce intestinal bacteria have been shown to lower this risk^[101]^.

In contrast, fish consumption, rich in omega-3 fatty acids with antiproliferative, anti-inflammatory, and antioxidant properties, may reduce bladder cancer risk by inhibiting cancer development through epigenetic mechanisms^[102]^. The dietary glycemic index (GI) is another factor linked to bladder cancer risk^[22]^. Diets with a high GI elevate blood glucose levels, leading to hyperinsulinemia and insulin resistance, which can promote cellular proliferation, inhibit apoptosis, and increase cancer risk^[103–105]^. Insulin further enhances cancer cell proliferation and invasion by activating the IGF-1 and stimulating oncogenic pathways such as PI3K/AKT/mTOR and RAS/RAF/MAPK^[106–108]^. A high-fat diet increases bladder cancer risk by changing the gut microbiota, boosting certain bacteria, and activating the mTORC1 pathway. This promotes myeloid-derived suppressor cells, which aid tumor progression^[109]^. Additionally, excessive consumption of fluids, particularly those containing disinfection by-products found in tap water, has been correlated with an elevated risk of bladder cancer^[110]^.

Cruciferous vegetables, including broccoli, are associated with a reduced risk of bladder cancer. These vegetables contain high levels of thioglucosides, phenolic compounds, and carotenoids, which manifest anticancer properties through the regulation of cell proliferation, inflammatory responses, and various other mechanisms^[111,112]^. Thioglucosides metabolize into isothiocyanates, potent molecules with anti-oncogenic capabilities that inhibit tumor cell migration, modulate epithelial-mesenchymal transition (EMT), and promote apoptosis^[113–116]^. Dietary patterns potentially influence bladder carcinogenesis. A WD, characterized by an excessive intake of animal fats, red meat, and refined carbohydrates, is associated with an elevated risk of bladder cancer^[20]^. In contrast, the MD, which is rich in plant-based foods like fruits, vegetables, and whole grains and includes lower consumption of meat and processed products, offers a protective effect against this disease^[20]^. Vitamin E is noted for its multifaceted benefits, including antioxidant, anti-inflammatory, and lipid-regulating properties, and its role in modulating crucial cellular signaling pathways, all contributing to a reduced cancer risk^[117]^. Milk and fermented dairy products also contribute to lowering bladder cancer risk due to their high content of vitamin E, calcium, and probiotics. These nutrients support cell differentiation, enhance intestinal flora, and provide anticancer benefits^[118,119]^. However, research indicates that extracellular vesicles (EVs) found in milk can trigger apoptosis and suppress tumor growth by transporting specific miRNAs; yet, they might also facilitate tumor metastasis under certain conditions^[120]^. Excessive consumption of whole milk may increase the risk of bladder cancer, primarily due to factors such as IGF-1, branched-chain amino acids, saturated fatty acids, and exosomal microRNAs (miRs), which promote cancer cell proliferation via the mTORC1 signaling pathway^[119,121,122]^.

### Disease risk factors

AAV is associated with an increased risk of bladder cancer, potentially due to its oncogenic effects mediated by prolonged immune activation and inflammatory responses. Specifically, abnormal T-cell activation and aberrant cytokine expression, such as IL-6 and IL-10, are implicated in the pathogenesis of cancer development^[123]^. Moreover, cyclophosphamide (CYC), a therapeutic commonly utilized in the treatment of AAV, significantly contributes to this increased cancer risk. Its metabolite, acrolein, exhibits pronounced carcinogenicity and toxicity to the bladder mucosa following urinary excretion, which not only leads to hemorrhagic cystitis^[124–126]^ but also promotes bladder dysfunction via mechanisms such as oxidative stress, inflammatory responses, and epithelial cell detachment^[127]^. Additionally, individuals with LS, an inherited disorder resulting from mutations in DNA repair genes (e.g., MLH1, MSH2, MSH6, PMS2) or deletions in the EPCAM gene^[128]^, face a heightened risk of bladder cancer^[9]^. The mechanism involves microsatellite instability (MSI), which escalates the risk of bladder cancer and other malignancies^[129,130]^. Likewise, patients with BPH exhibit an increased risk of bladder cancer^[42]^, likely due to chronic lower urinary tract damage from urine retention, which persistently exposes the bladder epithelium to carcinogens^[131]^.

### Drug risk factors

Opium consumption is potentially associated with a higher risk of bladder cancer^[4]^. The IARC categorizes opium as a Group 1 human carcinogen, substantiating its role in the etiology of various cancers, including those of the larynx, lung, and bladder. Although the precise carcinogenic mechanisms remain elusive, research indicates that the tar residues and combustion products of opium are genotoxic and may facilitate tumorigenesis through several pathways, including the induction of angiogenesis, immunosuppression, alterations in sphincter tone, and synergistic effects of impurities^[132,133]^. Additionally, Pioglitazone, a PPARγ agonist, has been associated with an increased likelihood of developing bladder cancer^[61]^. PPARγ agonists may promote bladder cancer development by increasing ROS production, disrupting the cell cycle and immune regulation, and inducing M2 macrophage polarization. These effects contribute to DNA damage, immune evasion, and tumor progression^[134,135]^.

PPARγ plays a dual role in bladder cancer, influenced by the tumor microenvironment and ligand type. It can inhibit tumor growth by activating the PTEN pathway, degrading NF-κB/p65 and MUC1-C, and reducing MMP-2/9. However, under conditions such as RXRA mutations or PPARG amplification, it may promote tumor progression and immune evasion via the SHH gene and AKT/GSK3β pathway. Its function also depends on ligand specificity and molecular modifications^[136,137]^. The association between antihypertensive drugs, such as ARBs and ACEIs, and the risk of bladder cancer remains uncertain. In vitro studies have suggested that ARBs might promote tumor cell proliferation, angiogenesis, and tumor progression^[138]^. Recent investigations have shown that ARBs, such as losartan, while inhibiting tumor cell growth in vitro, might enhance tumor progression and reduce the efficacy of immunotherapy in vivo, potentially due to a reduction in CD8+ T-cell infiltration within the tumor^[139]^. However, the majority of studies indicate that ARBs and ACEIs could exert potential antitumor effects by inhibiting the RAAS, improving the tumor microenvironment, and enhancing the effectiveness of immunotherapy^[140]^. Moreover, nitrosamine impurities found in ARB products have been identified as potentially carcinogenic, necessitating further research to mitigate these risks^[141]^. In contrast, 5-ARIs provide protective effects by decreasing the occurrence and progression of bladder cancer through the inhibition of the conversion of testosterone to dihydrotestosterone (DHT), regulation of tumor-associated protein expression, and blocking the procarcinogenic effects of 5α-reductase^[142]^.

### Environmental and lifestyle risk factors

Recent studies have linked PM2.5 and NO_2_ exposure to increased bladder cancer risk. Our analysis shows that every 5 µg/m^3^ rise in PM2.5 and 10 µg/m^3^ rise in NO_2_ are associated with higher risk. The WHO’s 2021 Air Quality Guidelines, which recommend annual exposure limits of 5 µg/m^3^ for PM2.5 and 10 µg/m^3^ for NO_2_, provide external support for our findings^[143]^. At the cellular level, PM2.5 promotes oxidative stress, leading to the production of ROS that inflict DNA damage^[144]^. Furthermore, PM2.5 incites an inflammatory response that elevates IL-6 and TNF-α levels, and it influences gene expression through epigenetic pathways, including DNA methylation and miRNA expression^[145]^. Similarly, NO_2_, indicative of traffic and fossil fuel emissions, may possess carcinogenic properties and potentially acts in synergy with other pollutants, though the precise mechanisms remain to be fully elucidated^[5]^. Smoking is a well-established risk factor for bladder cancer, introducing carcinogens like aromatic amines and polycyclic aromatic hydrocarbons that damage urothelial DNA. It also generates ROS, causing oxidative stress, impairing immune surveillance, and reducing the effectiveness of intravesical BCG therapy, all of which increase bladder cancer risk and impact treatment outcomes^[146]^. Physical activity protects against bladder cancer by reducing inflammation, enhancing immune function, and inhibiting tumor growth through modulation of cytokines and immune cell activity in the tumor microenvironment^[147]^.

### Physiological risk factors

Parity has been associated with a decreased risk of bladder cancer in women^[96]^. Hormonal changes during pregnancy may reduce bladder cancer risk by regulating estrogen and progesterone receptors. ERβ activation can suppress tumor-promoting factors and enhance immunity, while progesterone helps maintain immune balance and reduce immunosuppression^[148]^. Post-delivery, alterations in pelvic and pelvic floor structures may diminish the exposure of the bladder epithelium to urinary carcinogens, further decreasing cancer risk^[149,150]^. In contrast, tooth loss has been linked to an increased likelihood of bladder cancer. Periodontitis, extending beyond a localized dental condition, may heighten systemic cancer risk through mechanisms such as chronic inflammation, release of inflammatory mediators, microbial dissemination, immune dysregulation, and interactions with genetic factors^[151]^.

### Advantages

This umbrella review offers a comprehensive meta-analytic synthesis of risk factors associated with bladder cancer, encompassing six domains: diet, lifestyle, environmental exposures, occupational exposures, medication use, and physiological factors. In light of the significant incidence and recurrence rates of bladder cancer, this research contributes to clinical prevention and enhances public health initiatives, particularly in the realms of early screening, risk assessment, and the development of tailored intervention strategies. Employing a systematic methodology, the review amalgamates findings across various disciplines, establishing a robust scientific basis for the identification and management of bladder cancer risk factors. A meticulous process of literature screening and data extraction was implemented to guarantee the thoroughness and reliability of the findings. Where ample data were available, the aggregate effect sizes of the risk factors were recalculated using both random-effects and fixed-effects models. To augment the robustness of the study, assessments of heterogeneity and detection of publication bias were conducted. Moreover, employing both the AMSTAR 2 and GRADE methodologies, a rigorous evaluation of evidence quality for each health outcome was undertaken, affirming the scientific validity and credibility of the results.

### Evidence-based and multi-level prevention approaches

Effective prevention of bladder cancer necessitates a sophisticated, evidence-based approach that addresses both individual behaviors and broader environmental and systemic determinants. Based on currently available evidence, certain exposures have been suggested as modifiable and may represent potential targets for preventative strategies. Notably, opium consumption, ambient air pollution (specifically PM2.5 and NO_2_), and ANCA-associated vasculitis are consistently associated with an increased risk of bladder cancer. These findings underscore the need for urgent clinical and public health interventions, including opioid substitution therapies, the imposition of stringent air quality standards (e.g., reducing PM2.5 to below 5 µg/m^3^ and NO_2_ to below 10 µg/m^3^), and enhanced screening protocols for patients with ANCA-associated autoimmune diseases.

Apart from a few well-established high-risk factors, the impact of other potential contributors to bladder cancer risk often depends on the dosage, duration of exposure, and specific contextual factors. Among these, dietary factors are modifiable. For example, frequent consumption of red meat (≥100 g/day) or processed meat (≥50 g/day) may be associated with an increased risk, whereas higher intake of cruciferous vegetables (≥412.5 g/week) may offer protective effects. Additionally, diets with a high glycemic index, Western dietary patterns, high fat intake, and excessive alcohol consumption – particularly liquor or spirits (≥12 g/drink) – have also been linked to elevated risk. Based on current evidence, it is advisable to limit red meat intake to less than 100 g per day, increase cruciferous vegetable intake to at least 412.5 g per week, and reduce the consumption of high-sugar, high-fat, and high-alcohol products to mitigate potential risks.

In addition, reproductive and biological factors – such as early-onset breast cancer and hypertension (with each 10 mmHg increase in diastolic blood pressure) – may further modify individual risk profiles and support the case for more rigorous clinical monitoring. Conversely, some factors – including parity, regular physical activity, metformin use, aspirin therapy, and androgen suppression via 5-ARIs – have been linked to potential protective effects and may be considered in the development of tailored prevention strategies.

At the community level, interventions should focus on environmental equity, especially in areas with high pollution and limited access to healthcare. Strategies include free screening, risk communication, and subsidies for healthy food options. AI-based risk prediction and mobile health platforms can enhance prevention efforts. In conclusion, bladder cancer prevention requires precise risk identification, personalized interventions, and policy changes to reduce disease burden and improve health outcomes.

### Constraints

Despite its broad scope, this umbrella review has several limitations. Most included meta-analyses were of low or very low quality and showed high heterogeneity (I^2^ > 50%) due to differences in populations, exposures, outcomes, and study designs, which may affect the reliability of pooled estimates. As heterogeneity is inherent to umbrella reviews, we accounted for it in the GRADE assessments, often downgrading the certainty of evidence. This ensured that conclusions remained cautious and evidence-based. Future studies should adopt standardized definitions, consistent methods, and high-quality designs to strengthen the evidence on bladder cancer risk factors.

Secondly, some results showed inconsistencies. For example, the protective effect of cruciferous vegetables varied across studies^[21]^; alcohol consumption, particularly spirits, may increase the risk of bladder cancer^[28]^; evidence regarding milk intake is conflicting, with some studies suggesting a protective effect^[26]^, while data from the UK Biobank and FinnGen suggest an increased risk^[152]^. Furthermore, the relationship between ARBs and bladder cancer remains unclear^[62]^. These inconsistencies may arise from differences in study design, sample characteristics, and methodology, emphasizing the need for mechanistic and high-quality research. To address this, we conducted subgroup analyses across regions, genders, age groups, and study types to identify specific associations and assess potential modifiers. These analyses enhance our understanding of bladder cancer risk factors and guide future research.

Additionally, depression may be a consequence of bladder cancer and its treatments, thereby increasing the likelihood of reverse causation in observational studies. Tumor-associated inflammation may activate the hypothalamic-pituitary-adrenal (HPA) axis and disrupt neurotransmitter functionality, leading to depressive symptoms^[153]^. Furthermore, chemotherapy-related discomfort and psychological stress, particularly during the initial diagnostic phase, could exacerbate these symptoms^[154]^. These bidirectional mechanisms make causal inference challenging, underscoring the importance of prospective or mechanistic studies to more accurately determine the temporal sequence and direction of causality.

This review did not examine interactions between risk factors, such as the synergistic effects of smoking and air pollution. This omission may lead to an underestimation of the combined risks associated with these factors. Although we identified more intrinsic risk factors such as genetic susceptibility, polymorphisms, findings from Mendelian randomization, and enzyme functionality^[155–158]^, the focus of this review was on modifiable risk factors for bladder cancer to inform clinical prevention and public health interventions. Consequently, genetic and other micro-level factors, typically non-modifiable, were not included, potentially resulting in an incomplete depiction of all potential risk factors for bladder cancer. Future environmental research could emphasize critical areas, including conducting high-quality prospective cohort studies to robustly validate the association between long-term medication use and bladder cancer risk. Additionally, investigations into gene-environment interactions are necessary to understand how genetic predispositions may alter the impacts of environmental exposures such as air pollutants, diet, and pharmaceuticals.

Regarding literature inclusion, to ensure methodological consistency, reporting quality, and reproducibility, this review excluded grey literature and non-English publications, considering only peer-reviewed meta-analyses published in English with clearly defined criteria. Through the AMSTAR 2 quality assessment, we indirectly evaluated whether the included reviews had considered these sources. In terms of assessing publication bias, we referred to Egger’s test results from the original meta-analyses and conducted supplementary analyses when original data were available. However, the inability to further evaluate some studies due to data unavailability limited the comprehensiveness of our bias assessment. More critically, the generally low quality of the included meta-analyses precluded formal sensitivity analyses, underscoring the urgent need for high-quality systematic reviews and meta-analyses in this field.

## Conclusion

This comprehensive umbrella review summarizes the evidence on modifiable risk factors for bladder cancer, providing an evidence-based reference for prevention strategies. In addition to reaffirming the well-known roles of smoking and occupational exposures, the review also identifies various lifestyle, dietary, pharmaceutical, and environmental factors that may offer opportunities for targeted interventions. These findings highlight the importance of focusing prevention efforts on modifiable exposures to reduce the burden of bladder cancer.

Despite the heterogeneity and methodological limitations in the existing evidence, several factors have been identified as potential risk enhancers, including high intake of processed meat, exposure to specific medications such as pioglitazone and opioids, and certain environmental pollutants. On the other hand, increased intake of cruciferous vegetables and adherence to the Mediterranean dietary pattern seem to offer protective effects. These associations provide initial insights for health education, lifestyle modifications, and the formulation of public health policies.

Future research could benefit from focusing on a more integrated framework to deepen the understanding of bladder cancer risk factors. For instance, the exploration of the Microenvironment-Exposure-Susceptibility model could view bladder cancer as a result of dynamic interactions among external exposures, individual susceptibility, and local microenvironmental changes. Additionally, developing a risk prioritization strategy based on intervenability, strength of evidence, and population-level exposure would help optimize the allocation of public health resources. Emphasis should also be placed on individualized risk modeling and stratified prevention, integrating dietary patterns, comorbidities, medication history, and occupational exposures to promote precision public health. These directions reflect a shift from static risk aggregation to dynamic, system-level understanding and targeted prevention.

## Supplementary Material

## Data Availability

All data generated or analyzed during this study are included in this article. The data are available from the corresponding author upon reasonable request.

## Research registration unique identifying number (UIN)

This review is registered with PROSPERO (registration number: CRD42025632360).
