# Supplementary material for: Bladder cancer risk factors: a comprehensive umbrella review of meta-analyses
Source: Int J Surg. 2025 Sep 10;112(1):1479–503. doi: 10.1097/JS9.0000000000003371 (PMC12825672; doi:10.1097/JS9.0000000000003371)
Supplement: Supplementary file 1 [file js9-112-1479-001.docx]

**Supplementary Materials**

TableS1 Literature search strategy

pubmed：854

((("Urinary Bladder Neoplasms"[Mesh]) OR (((((((((((Urinary Bladder Neoplasm) OR (Bladder Neoplasms)) OR (Bladder Neoplasm)) OR (Bladder Tumors)) OR (Bladder Tumor)) OR (Urinary Bladder Cancer)) OR (Bladder Cancer)) OR (Bladder Cancers)) OR (Cancer of Bladder)) OR (Cancer of the Bladder)) OR (Malignant Tumor of Urinary Bladder))) AND (("Risk Factors"[Mesh]) OR ((((((((((Risk Factor) OR (Population at Risk)) OR (Populations at Risk)) OR (Risk Scores)) OR (Risk Score)) OR (Risk Factor Scores)) OR (Risk Factor Score)) OR (Health Correlates)) OR (Social Risk Factors)) OR (Social Risk Factor)))) AND (systematic review OR meta-analysis)

embase 719


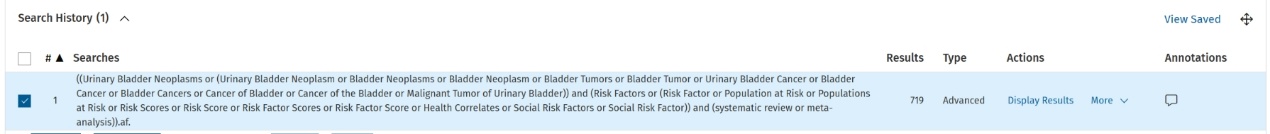


(((Urinary Bladder Neoplasms) OR (((((((((((Urinary Bladder Neoplasm) OR (Bladder Neoplasms)) OR (Bladder Neoplasm)) OR (Bladder Tumors)) OR (Bladder Tumor)) OR (Urinary Bladder Cancer)) OR (Bladder Cancer)) OR (Bladder Cancers)) OR (Cancer of Bladder)) OR (Cancer of the Bladder)) OR (Malignant Tumor of Urinary Bladder))) AND ((Risk Factors) OR ((((((((((Risk Factor) OR (Population at Risk)) OR (Populations at Risk)) OR (Risk Scores)) OR (Risk Score)) OR (Risk Factor Scores)) OR (Risk Factor Score)) OR (Health Correlates)) OR (Social Risk Factors)) OR (Social Risk Factor)))) AND (systematic review OR meta-analysis)

Cochrance 74


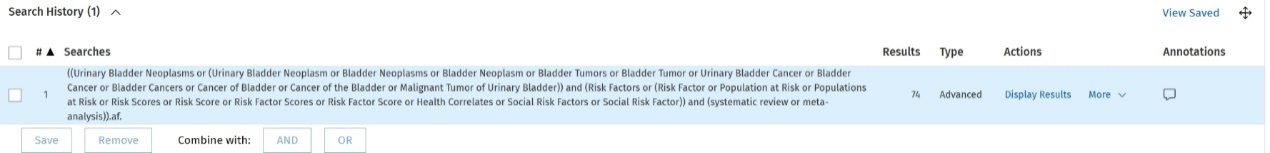


Web of Science 782

(((Urinary Bladder Neoplasms) OR (((((((((((Urinary Bladder Neoplasm) OR (Bladder Neoplasms)) OR (Bladder Neoplasm)) OR (Bladder Tumors)) OR (Bladder Tumor)) OR (Urinary Bladder Cancer)) OR (Bladder Cancer)) OR (Bladder Cancers)) OR (Cancer of Bladder)) OR (Cancer of the Bladder)) OR (Malignant Tumor of Urinary Bladder))) AND ((Risk Factors) OR ((((((((((Risk Factor) OR (Population at Risk)) OR (Populations at Risk)) OR (Risk Scores)) OR (Risk Score)) OR (Risk Factor Scores)) OR (Risk Factor Score)) OR (Health Correlates)) OR (Social Risk Factors)) OR (Social Risk Factor)))) AND (systematic review OR meta-analysis) (Topic)

Table S2 Subgroup analysis of different meat intakes and bladder cancer risk

| Subgroup | No. of studies | RR | 95%CI | I*^2^* |
| --- | --- | --- | --- | --- |
| **Total meat** | 11 | 1.10 | 0.92-1.31 | 55.20% |
| Study design |  |  |  |  |
| Case-control | 9 | 1.09 | 0.89-1.34 | 63.20% |
| Cohort | 2 | 1.13 | 0.81-1.58 | 0.00% |
| Region |  |  |  |  |
| Europe/USA | 8 | 1.11 | 0.86-1.38 | 61.60% |
| Others | 3 | 1.10 | 0.80-1.53 | 47.40% |
| Publication year |  |  |  |  |
| <2008 | 3 | 0.75 | 0.61-0.92 | 0.00% |
| ≥2008 | 8 | 1.3 | 1.14-1.49 | 0.00% |
| Quality score |  |  |  |  |
| <7 | 6 | 0.96 | 0.71-1.29 | 71.80% |
| ≥7 | 6 | 1.27 | 1.08-1.50 | 0.00% |
| **Red meat** | 20 | 1.23 | 1.08-1.39 | 51.30% |
| Study design |  |  |  |  |
| Case-control | 12 | 1.31 | 1.07-1.60 | 67.20% |
| Cohort | 8 | 1.11 | 0.97-1.27 | 0.00% |
| Region |  |  |  |  |
| Europe/USA | 13 | 1.16 | 1.00-1.34 | 55.70% |
| Others | 7 | 1.47 | 1.13-1.90 | 30.30% |
| Publication year |  |  |  |  |
| <2008 | 9 | 1.13 | 0.83-1.54 | 61.30% |
| ≥2008 | 11 | 1.26 | 1.11-1.44 | 42.50% |
| Quality score |  |  |  |  |
| <7 | 7 | 1.25 | 0.84-1.86 | 77.40% |
| ≥7 | 13 | 1.21 | 1.10-1.34 | 3.20% |
| **Processed meat** | 19 | 1.16 | 1.08-1.25 | 28.00% |
| Study design |  |  |  |  |
| Case-control | 13 | 1.21 | 1.10-1.33 | 38.30% |
| Cohort | 6 | 1.08 | 0.95-1.22 | 0.00% |
| Region |  |  |  |  |
| Europe/USA | 13 | 1.12 | 1.04-1.22 | 3.90% |
| Others | 6 | 1.39 | 1.15-1.67 | 41.30% |
| Publication year |  |  |  |  |
| <2009 | 9 | 1.15 | 1.02-1.29 | 23.60% |
| ≥2009 | 10 | 1.21 | 1.09-1.34 | 34.80% |
| Quality score |  |  |  |  |
| <7 | 7 | 1.15 | 1.00-1.32 | 20.80% |
| ≥7 | 12 | 1.15 | 1.07-1.28 | 36.80% |
| **White meat** | 15 | 0.96 | 0.83-1.10 | 53.70% |
| Study design |  |  |  |  |
| Case-control | 9 | 0.95 | 0.75-1.19 | 65.80% |
| Cohort | 6 | 0.99 | 0.85-1.14 | 24.90% |
| Region |  |  |  |  |
| Europe/USA | 11 | 1.01 | 0.87-1.17 | 58.00% |
| Others | 4 | 0.75 | 0.57-0.99 | 0.00% |
| Publication year |  |  |  |  |
| <2008 | 5 | 0.8 | 0.55-1.16 | 74.30% |
| ≥2008 | 9 | 0.99 | 0.88-1.15 | 25.10% |
| Quality score |  |  |  |  |
| <7 | 5 | 0.83 | 0.57-1.20 | 73.00% |
| ≥7 | 9 | 1.01 | 0.88-1.15 | 34.00% |
| **Fish** | 15 | 0.8 | 0.67-0.95 | 62.90% |
| Study design |  |  |  |  |
| Case-control | 11 | 0.8 | 0.65-0.98 | 64.50% |
| Cohort | 4 | 0.78 | 0.52-1.18 | 68.60% |
| Region |  |  |  |  |
| Europe/USA | 11 | 0.86 | 0.71-1.05 | 63.70% |
| Others | 4 | 0.6 | 0.42-0.87 | 45.40% |
| Publication year |  |  |  |  |
| <2008 | 7 | 0.69 | 0.45-1.06 | 79.40% |
| ≥2008 | 8 | 0.84 | 0.73-0.96 | 19.50% |
| Quality score |  |  |  |  |
| <7 | 7 | 0.75 | 0.52-1.08 | 78.00% |
| ≥7 | 8 | 0.81 | 0.69-0.94 | 28.90% |

Notes: No, number; RR, relative risk; CI: Confidence interval

Table S3 Subgroup analysis of 125 ml /d increment of Coffee consumption and bladder cancer risk

| Subgroup | No. of studies | RR | 95%CI | I*^2^* |
| --- | --- | --- | --- | --- |
| Total | 15 | 1.01 | 0.98-1.03 | 56.30% |
| Geographic region |  |  |  |  |
| US | 7 | 1.01 | 0.97-1.04 | 70.00% |
| Europe | 5 | 1.02 | 0.99-1.04 | 0% |
| Japan | 3 | 0.92 | 0.75-1.14 | 65.30% |
| Duration of follow-up |  |  |  |  |
| ≥ 10 yr | 11 | 1.01 | 0.98–1.04 | 62.60% |
| < 10 yr | 4 | 1 | 0.95–1.04 | 42.30% |
| No. of participants |  |  |  |  |
| ≥ 50,000 | 8 | 1 | 0.98-1.03 | 61.10% |
| < 50,000 | 7 | 1.04 | 0.97-1.13 | 52% |
| Sex |  |  |  |  |
| Men | 9 | 1.01 | 0.98-1.05 | 42.00% |
| Women | 6 | 0.98 | 0.89-1.08 | 56% |
| No. of cases |  |  |  |  |
| ≥ 200 | 10 | 1 | 0.98-1.02 | 57.30% |
| < 200 | 5 | 1.12 | 1.04-1.21 | 0% |
| % Male cases |  |  |  |  |
| ≥ 75% | 6 | 1.02 | 0.94-1.10 | 69.30% |
| < 75% | 7 | 1.01 | 0.99-1.04 | 18.50% |
| % Current smoker |  |  |  |  |
| ≥ 25% | 7 | 1 | 0.94-1.07 | 52.60% |
| < 25% | 8 | 1.01 | 0.98-1.03 | 63.60% |
| Smoking status |  |  |  |  |
| Never | 6 | 1.02 | 0.95-1.09 | 58.00% |
| Former | 3 | 1.01 | 0.97-1.05 | 69.70% |
| Never/former | 6 | 1.03 | 0.98-1.08 | 76.30% |
| Current | 3 | 1.04 | 1.01-1.07 | 0% |
| Former/current | 4 | 1.01 | 0.95-1.07 | 38.40% |
| Statistical adjustment |  |  |  |  |
| Smoking |  |  |  |  |
| Poorer | 3 | 1.08 | 1.02-1.15 | 8.90% |
| Moderate | 7 | 0.98 | 0.90-1.07 | 60.50% |
| Better | 5 | 1 | 0.98-1.02 | 49.70% |
| Alcohol drinking |  |  |  |  |
| No | 11 | 1.02 | 0.99-1.04 | 34.50% |
| Yes | 4 | 0.98 | 0.92-1.04 | 68.80% |
| Education |  |  |  |  |
| No | 9 | 1.03 | 0.99-1.07 | 44.10% |
| Yes | 6 | 0.99 | 0.96-1.03 | 69.50% |
| Physical activity |  |  |  |  |
| No | 12 | 1.01 | 0.97-1.05 | 53.60% |
| Yes | 3 | 1 | 0.97-1.04 | 75.70% |
| BMI |  |  |  |  |
| No | 12 | 1.02 | 0.99-1.05 | 53.60% |
| Yes | 3 | 0.98 | 0.93-1.03 | 75.70% |
| Diabetes |  |  |  |  |
| No | 12 | 1.01 | 0.98-1.04 | 43.20% |
| Yes | 3 | 0.99 | 0.92-1.05 | 78.40% |
| Family history of cancer |  |  |  |  |
| No | 13 | 1.01 | 0.98-1.05 | 49.90% |
| Yes | 2 | 1 | 0.96-1.04 | 87.00% |
| Energy intake |  |  |  |  |
| No | 13 | 1.01 | 0.99-1.04 | 59.00% |
| Yes | 2 | 0.98 | 0.91-1.04 | 51.30% |
| Fruit/vegetable consumption |  |  |  |  |
| No | 11 | 1.02 | 0.98-1.06 | 51.50% |
| Yes | 4 | 1 | 0.97-1.03 | 72.10% |
| Tea consumption |  |  |  |  |
| No | 10 | 1.02 | 0.99-1.05 | 40.80% |
| Yes | 5 | 0.99 | 0.96-1.03 | 62.10% |

Notes: No, number; RR, Relative risk; CI: Confidence interval

Table S4 Subgroup analysis of milk intake and bladder cancer risk

| Subgroup | No. of studies | RR | 95%CI | I*^2^* |
| --- | --- | --- | --- | --- |
| Gender |  |  |  |  |
| Male | 4 | 0.86 | 0.65-1.07 | 78.70% |
| Female | 3 | 0.97 | 0.85-1.08 | 68.20% |
| Geographical location |  |  |  |  |
| Americas | 7 | 0.88 | 0.76-1.01 | 74.90% |
| Europe | 6 | 0.96 | 0.79-1.13 | 51.20% |
| Asia | 5 | 0.8 | 0.57-1.04 | 69.40% |
| Type of design |  |  |  |  |
| Case-control | 12 | 0.9 | 0.80-1.01 | 72.40% |
| Cohort | 6 | 0.86 | 0.70-1.02 | 31.60% |

Notes: No, number; RR, Relative risk; CI: Confidence interval

Table S5 Subgroup analysis of total fluid intake and bladder cancer risk

| Subgroup | No. of studies | RR | 95%CI | I*^2^* |
| --- | --- | --- | --- | --- |
| Total | 20 | 1.16 | 1.00-1.36 | 72.30% |
| Type of design |  |  |  |  |
| Cohort | 4 | 0.98 | 0.85-1.13 | 0% |
| Case-control | 16 | 1.23 | 1.01-1.49 | 75.80% |
| Gender |  |  |  |  |
| Male | 12 | 1.37 | 1.04-1.81 | 83.30% |
| Female | 12 | 1.03 | 0.78-1.35 | 48.70% |
| Geographical Region |  |  |  |  |
| America | 9 | 1.23 | 0.99-1.54 | 78.70% |
| Europe | 8 | 1.13 | 0.88-1.45 | 60.70% |
| Asia | 3 | 1 | 0.64-1.57 | 67.80% |

Notes: No, number; RR, Relative risk; CI: Confidence interval

Table S6 Subgroup analysis of vitamin E intake and bladder cancer risk

| Subgroup | No. of studies | RR | 95%CI | I*^2^* |
| --- | --- | --- | --- | --- |
| Total | 8 | 0.84 | 0.72-0.96 | 21.40% |
| Study design |  |  |  |  |
| Cohort | 8 | 0.84 | 0.72-0.96 | 21.40% |
| RCT | 3 | 1.1 | 0.86-1.35 | 0.00% |
| Gender |  |  |  |  |
| Male | 7 | 0.86 | 0.74-0.98 | 28.90% |
| Male + Female | 3 | 1 | 0.80-1.20 | 0.00% |
| Female | 2 | 0.83 | 0.07-1.60 | 85.90% |
| Geographic location |  |  |  |  |
| USA | 7 | 0.84 | 0.70-0.98 | 30.70% |
| Europe | 4 | 0.96 | 0.79-1.13 | 0.00% |
| Duration of follow-up |  |  |  |  |
| <10 years | 5 | 0.96 | 0.79-1.12 | 0.00% |
| >10 years | 6 | 0.84 | 0.70-0.98 | 45.90% |

Notes: No, number; RR, Relative risk; CI: Confidence interval

Table S7 Subgroup analysis of alcohol consumption and bladder cancer risk

| Subgroup | No. of studies | RR | 95%CI | I*^2^* |
| --- | --- | --- | --- | --- |
| gender |  |  |  |  |
| all gender | 8 | 1.07 | 0.95-1.20 | 65.30% |
| male | 4 | 1.23 | 1.13-1.35 | 3.70% |
| female | 5 | 0.93 | 0.82-1.04 | 38.40% |
| different sources |  |  |  |  |
| Beer | 5 | 1.01 | 0.82-1.23 | 75.90% |
| Beer-male | 4 | 1.09 | 0.91-1.31 | 67% |
| Beer-female | 1 | 0.99 | 0.83-1.18 | NA |
| Wine | 5 | 1.05 | 0.90-1.23 | 55.90% |
| Wine-male | 4 | 1.10 | 0.94-1.28 | 48.10% |
| Wine-female | 1 | 0.95 | 0.81-1.11 | NA |
| Liquor or spirits | 6 | 1.21 | 1.04-1.41 | 53.70% |
| Liquor or spirits-male | 4 | 1.19 | 1.03-1.38 | 53.40% |
| Liquor or spirits-female | 1 | 0.97 | 0.81-1.16 | NA |
| intensity |  |  |  |  |
| Light | 2 | 0.96 | 0.84-1.08 | 0.00% |
| Moderate | 2 | 0.9 | 0.53-1.51 | 55.30% |
| Heavy | 2 | 0.97 | 0.69-1.37 | 48% |

Notes: No, number; RR, Relative risk; CI: Confidence interval

Table S8 Subgroup analysis of dietary fat intake and bladder cancer risk

| Subgroup | No. of studies | RR | 95%CI | I*^2^* |
| --- | --- | --- | --- | --- |
| Total | 10 | 1.28 | 1.04-1.58 | 53.20% |
| Fat type |  |  |  |  |
| Saturated fat | 5 | 1.163 | 0.735-1.842 | 75.60% |
| Monounsaturated fat | 4 | 1.049 | 0.801-1.373 | 33.00% |
| Polyunsaturated fat | 5 | 0.926 | 0.686-1.248 | 50.20% |
| Animal fat | 2 | 1.537 | 0.178-13.261 | 88.90% |
| Study design |  |  |  |  |
| Cohort | 2 | 0.983 | 0.834-1.160 | 0.00% |
| Case-control | 9 | 1.432 | 1.153-1.778 | 29.20% |
| PBCC | 4 | 1.198 | 0.721-1.992 | 48.70% |
| HBCC | 5 | 1.501 | 1.196-1.883 | 19.20% |
| Geographic locations |  |  |  |  |
| Europe | 6 | 1.359 | 1.027-1.798 | 63.90% |
| North America | 5 | 1.159 | 0.794-1.692 | 46.40% |

Notes: No, number; RR, Relative risk; CI: Confidence interval；PBCC: population-based case-control studies; HBCC: hospital-based case-control studies.

Table S9 Subgroup analysis of BPH and bladder cancer risk

| Subgroup | No. of studies | RR | 95%CI | I*^2^* |
| --- | --- | --- | --- | --- |
| Total | 6 | 1.71 | 1.39-2.11 | 32% |
| Study design |  |  |  |  |
| Case-control study | 2 | 2.5 | 1.63-3.84 | 0% |
| Cohort-study | 4 | 1.58 | 1.28-1.95 | 31% |
| Ethnicity |  |  |  |  |
| Caucasian | 3 | 1.51 | 1.03-2.22 | 22% |
| Asian | 2 | 1.95 | 1.44-2.63 | 40% |
| Source of participants |  |  |  |  |
| Population-based study | 2 | 2.18 | 1.17-4.06 | 0% |
| Hospital-based study | 3 | 1.71 | 1.27-2.32 | 68% |
| Quality of included studies |  |  |  |  |
| NOS score＞6 | 5 | 1.75 | 1.38-2.22 | 43% |
| NOS score≤6 | 1 | 1.4 | 0.71-2.67 | NA |

Notes: BPH, Benign prostatic hyperplasia; No, number; RR, Relative risk; CI: Confidence interval; NA, Not Available

Table S10 Subgroup analysis of lupus erythematosus and bladder cancer risk

| Subgroup | No. of studies | RR | 95%CI | I*^2^* |
| --- | --- | --- | --- | --- |
| Total | 12 | 1.66 | 1.07-2.59 | 85.90% |
| geographic region |  |  |  |  |
| European countries | 5 | 1.81 | 1.05-3.12 | NA |
| Asian countries | 4 | 2.46 | 0.65-9.29 | NA |

Notes: No, number; RR, Relative risk; CI: Confidence interval; NA, Not Available

Table S11 Subgroup analysis of radiotherapy for prostate cancer and bladder cancer risk

| Subgroup | No. of studies | OR | 95%CI | I*^2^* |
| --- | --- | --- | --- | --- |
| Total | 16 | 1.6 | 1.33-1.92 | 83.60% |
| Radiotherapy without description | 10 | 1.79 | 1.35-2.37 | 85.30% |
| EBRT | 3 | 1.27 | 1.20-1.34 | 0% |
| Brachytherapy | 3 | 1.33 | 0.87–2.05 | 12.30% |

Notes: EBRT, External beam radiotherapy; No, number; OR, Odds ratio; CI: Confidence interval

Table S12 Subgroup analysis of renal transplantation and bladder cancer risk

| Subgroup | No. of studies | RR | 95%CI | I*^2^* |
| --- | --- | --- | --- | --- |
| Total | 11 | 3.18 | 1.34-7.53 | 98.00% |
| ethnicity |  |  |  |  |
| European | 8 | 2 | 1.51-2.65 | 70.50% |
| Asian | 3 | 14.74 | 3.66-59.35 | 93.70% |

Notes: No, number; RR, Relative risk; CI: Confidence interval

Table S13 Subgroup analysis of diabetes and bladder cancer risk

| Subgroup | No. of studies | RR | 95%CI | I*^2^* |
| --- | --- | --- | --- | --- |
| Total | 17 | 1.23 | 1.16-1.31 | 83.30% |
| Gender |  |  |  |  |
| Women | 10 | 1.23 | 1.12-1.34 | 56.50% |
| Men | 16 | 1.21 | 1.13-1.29 | 80.10% |

Notes: No, number; RR, Relative risk; CI: Confidence interval

Table S14 Subgroup analysis of urinary calculi and bladder cancer risk

| Subgroup | No. of studies | OR | 95%CI | I*^2^* |
| --- | --- | --- | --- | --- |
| Total | 13 | 1.87 | 1.45-2.41 | 86.50% |
| Calculi location | 13 | 1.87 | 1.45–2.41 | 86.50% |
| Bladder calculi | 9 | 2.17 | 1.52–3.08 | 77.80% |
| Kidney calculi | 5 | 1.39 | 1.06–1.82 | 77.20% |
| EBRT,External beam radiotherapy;No, number; OR, Odds ratio;CI:Confidence interval |  |  |  |  |
| Male | 7 | 2.04 | 1.41–2.96 | 84.10% |
| Female | 6 | 2.99 | 2.37–3.76 | 0.00% |
| Study design |  |  |  |  |
| Case-control study | 10 | 1.75 | 1.25–2.45 | 84.90% |
| Cohort study | 3 | 2.27 | 1.55–3.32 | 89.50% |
| Geographic |  |  |  |  |
| America | 5 | 1.6 | 1.15–2.24 | 66.10% |
| Europe | 4 | 1.36 | 1.14–1.64 | 34.00% |
| Asia | 4 | 3.05 | 2.21–4.21 | 84.40% |

Notes: No, number; OR, Odds ratio; CI: Confidence interval

Table S15 Subgroup analysis of pioglitazone use and bladder cancer risk

| Subgroup | No. of studies | OR | 95%CI | I*^2^* |
| --- | --- | --- | --- | --- |
| Total | 19 | 1.13 | 1.03-1.25 | 31.30% |
| Smoking adjusted |  |  |  |  |
| Yes | 6 | 1.28 | 1.02-1.61 | 43.10% |
| No | 13 | 1.03 | 0.98-1.08 | 0% |
| Region of study |  |  |  |  |
| Europe | 8 | 1.17 | 1.00-1.36 | 67.60% |
| United States | 9 | 1.03 | 0.78-1.36 | 0% |
| Asia | 2 | 1.11 | 0.92-1.34 | 0% |
| Source of funding |  |  |  |  |
| Industry (Takeda) | 5 | 1 | 0.85-1.19 | 0% |
| Other | 14 | 1.2 | 1.05-1.36 | 43.80% |
| Type of comparators |  |  |  |  |
| Never use of Thiazolidinedione | 3 | 1.62 | 1.27-2.08 | 0% |
| Never use of Pioglitazone | 13 | 1.04 | 0.99-1.09 | 0% |
| Rosiglitazone | 1 | 1.14 | 0.79-1.65 | NA |
| Insulin | 1 | 0.92 | 0.63-1.34 | NA |
| Placebo | 1 | 0.65 | 0.33-1.28 | NA |
| Design of study |  |  |  |  |
| Cohort studies | 12 | 1.12 | 1.00-1.24 | 40.10% |
| Case–control studies | 7 | 1.21 | 0.96-1.56 | 7.50% |
| Sex |  |  |  |  |
| Men | 3 | 1.12 | 0.96-1.31 | 67.30% |
| Women | 3 | 1.01 | 0.98-1.08 | 0% |
| Cumulative dose-1 |  |  |  |  |
| ≤10.5 g | 6 | 1.17 | 0.99-1.39 | 0% |
| 10.5–28 g | 4 | 1.27 | 1.05-1.54 | 0% |
| >28 g | 4 | 1.66 | 1.32-2.07 | 0% |
| Cumulative dose-2 |  |  |  |  |
| ≤14 g | 2 | 0.96 | 0.79-1.17 | 0% |
| 14–40 g | 2 | 1.07 | 0.86-1.32 | 0% |
| >40 g | 2 | 0.92 | 0.58-1.44 | 43.50% |
| Cumulative duration-1 |  |  |  |  |
| ≤1 years | 9 | 1.07 | 0.92-1.23 | 33.90% |
| 1–2 years | 7 | 1.25 | 1.11-1.40 | 0.20% |
| >2 years | 7 | 1.49 | 1.21-1.84 | 57.50% |
| Cumulative duration-2 |  |  |  |  |
| ≤1.5 years | 2 | 0.97 | 0.78-2.82 | 0% |
| 1.5–4 years | 2 | 0.94 | 0.73-1.22 | 20.50% |
| >4 years | 2 | 1.11 | 0.85-1.44 | 0% |

Notes: No, number; OR, Odds ratio; CI: Confidence interval; NA, Not Available

Table S16 Subgroup analysis of aspirin intake and bladder cancer risk

| Subgroup | No. of studies | RR | 95%CI | I*^2^* |
| --- | --- | --- | --- | --- |
| Total | 31 | 1.01 | 0.97-1.04 | 0% |
| Regular users | 20 | 1.01 | 0.97-1.05 | 5.70% |
| Non-regular users | 7 | 1.03 | 0.94-1.12 | 0% |
| Mean duration of use ≥ years | 9 | 0.92 | 0.88-0.96 | 81.30% |
| Mean duration of use < 5 years | 9 | 0.9 | 0.84-0.96 | 68.40% |
| gender |  |  |  |  |
| Male | 5 | 1.03 | 0.96-1.10 | 60.80% |
| Female | 5 | 1.01 | 0.87-1.18 | 0% |
| smoking status |  |  |  |  |
| Non-smokers | 5 | 0.97 | 0.85-1.10 | 0% |
| Former smokers | 7 | 1.07 | 0.99-1.17 | 0% |
| current smokers | 5 | 0.9 | 0.78-1.03 | 35.20% |
| region |  |  |  |  |
| Multi-regions | 4 | 0.97 | 0.91-1.04 | 0% |
| Asia | 3 | 1.01 | 0.95-1.07 | 46.70% |
| Europe | 5 | 1.11 | 1.00-1.24 | 0% |
| North America | 19 | 1 | 0.95-1.06 | 0% |
| study type |  |  |  |  |
| Cohort study | 18 | 1.01 | 0.97-1.05 | 0% |
| Case control study | 12 | 1 | 0.94-1.06 |  |
| Others | 1 | 0.94 | 0.64-1.38 | NA |

Notes: No, number; RR, Relative risk; CI: Confidence interval；NA, Not Available

Table S17 Subgroup analysis of ARB use and bladder cancer risk

| Subgroup | No. of studies | RR | 95%CI | I*^2^* |
| --- | --- | --- | --- | --- |
| Total | 5 | 1.07 | 1.03-1.11 | 0% |
| Hypertension adjusted |  |  |  |  |
| Yes | 2 | 1.1 | 1.04-1.15 | 0% |
| No | 3 | 1.03 | 0.96-1.10 | 0% |
| Smoking adjusted |  |  |  |  |
| Yes | 2 | 1.03 | 0.96-1.10 | 0% |
| No | 3 | 1.1 | 1.04-1.15 | 0% |

Notes: ARB, Angiotensin II receptor blockers; No, number; RR, Relative risk; CI: Confidence interval

Table S18 Subgroup analysis of smoking intensity and bladder cancer risk

| Subgroup | No. of studies | RR | 95%CI | I*^2^* |
| --- | --- | --- | --- | --- |
| Total | 31 | 1.83 | 1.75-1.91 | 92.60% |
| region |  |  |  |  |
| China | 3 | 1.52 | 1.30-1.77 | 7.90% |
| Europe | 12 | 1.65 | 1.52-1.80 | 74.10% |
| USA | 9 | 2.15 | 2.01-2.30 | 96.90% |
| Turkey | 1 | 1.21 | 0.85-1.71 | NA |
| Belgian | 1 | 1.65 | 1.29-2.10) | NA |
| Canada | 1 | 1.20 | 0.95-1.52 | NA |
| Uruguay | 1 | 1.35 | 0.81-2.26 | NA |
| Egypt | 1 | 1.29 | 0.98-1.69 | NA |
| Australia | 1 | 2.81 | 2.21-3.58 | 0% |

Notes: No, number; RR, Relative risk; CI: Confidence interval; NA, Not Available

Table S19 Subgroup analysis of hairdresser occupation and bladder cancer risk

| Subgroup | No. of studies | RR | 95%CI | I*^2^* |
| --- | --- | --- | --- | --- |
| Total | 42 | 1.34 | 1.21-1.48 | NA |
| Gender |  |  |  |  |
| female | 17 | 1.25 | 1.05-1.50 | NA |
| male | 32 | 1.52 | 1.34-1.72 | NA |
| Study period |  |  |  |  |
| Ascertainment of cases < 1979 | 19 | 1.37 | 1.19-1.58 | NA |
| Ascertainment of cases 1980-1989 | 11 | 1.24 | 1.00-1.53 | NA |
| Ascertainment of cases > 1990 | 10 | 1.42 | 1.16-1.75 | NA |
| Study area |  |  |  |  |
| USA/Canada | 20 | 1.28 | 1.08-1.52 | NA |
| Europe/Nordic countries | 20 | 1.34 | 1.19-1.52 | NA |
| Adjusted data |  |  |  |  |
| Adjusted for smoking | 23 | 1.35 | 1.13-1.61 | NA |
| No adjustment | 19 | 1.33 | 1.18-1.50 | NA |
| Study quality level |  |  |  |  |
| High quality | 11 | 1.35 | 1.03-1.77 | NA |
| Moderate quality | 31 | 1.34 | 1.20-1.49 | NA |

Notes: No, number; RR, Relative risk; CI: Confidence interval

Table S20 Subgroup analysis of rubber-manufacturing industry exposure and bladder cancer risk

| Subgroup | No. of studies | RR | 95%CI | I*^2^* |
| --- | --- | --- | --- | --- |
| Total | 54 | 1.36 | 1.18-1.57 | 57% |
| Study design: |  |  |  |  |
| Cohort studies | 35 | 1.32 | 1.08-1.60 | 67% |
| Case-control studies | 19 | 1.43 | 1.20-1.71 | 4% |
| Gender: |  |  |  |  |
| Men | 43 | 1.26 | 1.09-1.45 | 43% |
| Women | 10 | 2.23 | 1.39-3.60 | 0% |
| Mortality/incidence: |  |  |  |  |
| Cancer mortality | 29 | 1.24 | 1.01-1.54 | 56% |
| Cancer incidence | 30 | 1.42 | 1.19-1.7 | 53% |
| Type of industry: |  |  |  |  |
| Tyre industry | 17 | 1.19 | 0.95-1.48 | 0% |
| General rubber goods industry | 12 | 1.19 | 0.93-1.51 | 0% |
| Employment characteristics: |  |  |  |  |
| Hired after 1960 | 8 | 1.06 | 0.66-1.71 | 0% |
| Hired after 1970 | 6 | 0.81 | 0.40-1.64 | 0% |
| With the longest duration of employment | 7 | 1.45 | 0.98-2.13 | 0% |

Notes: No, number; RR, Relative risk; CI: Confidence interval

Table S21 Subgroup analysis of benzene exposure and bladder cancer risk

| Subgroup | No. of studies | RR | 95%CI | I*^2^* |
| --- | --- | --- | --- | --- |
| Total | 35 | 1.07 | 0.97-1.18 | 44.40% |
| Region |  |  |  |  |
| North America | 12 | 1.06 | 0.78–1.43 | NA |
| Europe | 18 | 1.06 | 0.99–1.13 | NA |
| Others | 5 | 1.08 | 0.69–1.71 | NA |
| Study design |  |  |  |  |
| Case–control | 5 | 1.17 | 0.95–1.45 | NA |
| Cohort | 30 | 1.04 | 0.92–1.19 | NA |
| Quality score |  |  |  |  |
| Low quality (<8) | 14 | 1.07 | 0.97–1.18 | NA |
| High quality (≥8) | 21 | 1.05 | 0.87–1.28 | NA |
| Outcome |  |  |  |  |
| Incidence | 17 | 1.1 | 1.01–1.20 | NA |
| Mortality | 23 | 0.99 | 0.83–1.19 | NA |
| Years of publication |  |  |  |  |
| <2000 | 20 | 1.02 | 0.86–1.21 | NA |
| ≥2000 | 15 | 1.11 | 1.01–1.21 | NA |
| Sex |  |  |  |  |
| Men | 22 | 1.07 | 0.92–1.24 | NA |
| Women | 3 | 1.15 | 0.94–1.40 | NA |
| Both sexes | 16 | 1.06 | 1–1.13 | NA |
| Dose category |  |  |  |  |
| Low | 5 | 1.19 | 0.92–1.53 | NA |
| Medium | 4 | 1.06 | 0.99–1.13 | NA |
| High | 5 | 1.2 | 0.81–1.79 | NA |
| Industry |  |  |  |  |
| Oil industry | 15 | 0.98 | 0.87–1.12 | NA |
| Chemical industry | 3 | 2.02 | 0.96–4.24 | NA |
| Other industries | 7 | 0.92 | 0.71–1.19 | NA |
| Benzene exposure across multiple industries | 6 | 1.09 | 0.96–1.24 | NA |
| Mixed | 4 | 1.08 | 0.74–1.58 | NA |
| Adjustment for tobacco smoking |  |  |  |  |
| No | 30 | 1.04 | 0.93–1.15 | NA |
| Yes | 5 | 1.28 | 0.96–1.72 | NA |

Notes: No, number; RR, Relative risk; CI: Confidence interval

Table S22 Subgroup analysis of physical activity and bladder cancer risk

| Subgroup | No. of studies | RR | 95%CI | I*^2^* |
| --- | --- | --- | --- | --- |
| Total | 15 | 0.85 | 0.74-0.98 | 83% |
| Gender |  |  |  |  |
| Men | 12 | 0.92 | 0.82-1.05 | 67% |
| Women | 4 | 0.83 | 0.72-0.94 | 0% |
| Men and women combined | 2 | 0.48 | 0.24-0.96 | 75% |
| Intensity of physical activity |  |  |  |  |
| Moderate | 11 | 0.85 | 0.75-0.98 | 76% |
| Vigorous | 11 | 0.8 | 0.64-1.00 | 87% |
| Component or measure of physical activity |  |  |  |  |
| Energy expenditure | 5 | 0.76 | 0.49-1.17 | 83% |
| Activity duration | 4 | 0.9 | 0.77-1.05 | 0% |
| Activity frequency | 5 | 0.85 | 0.75-0.97 | 0% |
| Qualitative measures | 4 | 0.92 | 0.72-1.18 | 92% |
| Timing in life of physical activity |  |  |  |  |
| Recent | 10 | 0.84 | 0.66-1.07 | 82% |
| Consistent over time | 2 | 0.85 | 0.63-1.13 | 9% |
| Past | 6 | 0.87 | 0.71-1.06 | 84% |
| Type of physical activity assessment |  |  |  |  |
| Interview | 4 | 0.78 | 0.39-1.56 | 91% |
| Self reported | 11 | 0.84 | 0.76-0.92 | 46% |
| By proxy | 3 | 1.05 | 0.79-1.39 | 58% |
| Adjustment for body mass index |  |  |  |  |
| Adjusted for body mass index | 8 | 0.78 | 0.58-1.06 | 86% |
| Not adjusted for bodymass index | 10 | 0.91 | 0.79-1.04 | 68% |
| Adjustment for smoking |  |  |  |  |
| Adjusted for smoking | 13 | 0.83 | 0.69-1.01 | 75% |
| Not adjusted for smoking | 5 | 0.9 | 0.72-1.12 | 88% |
| Study geographic region |  |  |  |  |
| North America | 10 | 0.75 | 0.62-0.93 | 72% |
| Europe | 7 | 0.98 | 0.79-1.22 | 87% |
| Asia | 1 | 0.94 | 0.77-1.15 | NA |

Notes: No, number; RR, Relative risk; CI: Confidence interval；NA, Not Available

Table S23 Subgroup analysis of pesticide exposure and bladder cancer risk

| Subgroup | No. of studies | OR | 95%CI | I*^2^* |
| --- | --- | --- | --- | --- |
| Total | 10 | 1.65 | 1.22-2.22 | 80.60% |
| Study design |  |  |  |  |
| Case-control | 7 | 2.075 | 1.183-3.638) | 84.70% |
| Cohort | 3 | 1.191 | 0.979-1.448 | 14.70% |
| Gender |  |  |  |  |
| Male | 5 | 1.154 | 1.020-1.306 | 23.30% |
| Female | 1 | NA | NA | NA |
| Region |  |  |  |  |
| Europe | 3 | 1.187 | 0.722-1.951 | 66.70% |
| America | 5 | 1.741 | 1.270-2.388 | 0% |
| Africa | 2 | 2.619 | 0.507-13.52 | 96.50% |
| Exposure assessment |  |  |  |  |
| Questionnaire | 2 | 2.177 | 0.975-4.859 | 44.20% |
| Interview | 4 | 2.457 | 0.755-7.989 | 89.00% |
| Database | 4 | 1.148 | 1.079-1.221 | 0% |
| Control factors |  |  |  |  |
| >3 | 3 | 1.607 | 1.065-2.423 | 48.90% |
| ≤ 3 | 7 | 1.752 | 0.659-4.657 | 93.70% |
| Study quality |  |  |  |  |
| High | 3 | 1.17 | 1.001-1.368 | 44.30% |
| Low | 7 | 1.959 | 1.081-3.550 | 79.90% |

Notes: No, number; OR, Odds risk; CI: Confidence interval; NA, Not Available

Table S24 Subgroup analysis of parity and bladder cancer risk.

| Subgroup | No. of studies | RR | 95%CI | I*^2^* |
| --- | --- | --- | --- | --- |
| Total | 13 | 0.76 | 0.70-0.82 | 0% |
| Number of parity |  |  |  |  |
| 1-2 vs.0 | 7 | 0.82 | 0.71-0.94 | 17.70% |
| 3-4 vs.0 | 7 | 0.79 | 0.68-0.91 | 0% |
| ≥ 5 vs.0 | 6 | 0.76 | 0.66-0.88 | 0% |
| Study design |  |  |  |  |
| Cohort study | 6 | 0.77 | 0.71–0.84 | 0% |
| Case-control study | 7 | 0.71 | 0.60–0.83 | 0% |
| Number of cases |  |  |  |  |
| < 250 | 7 | 0.68 | 0.56–0.81 | 0% |
| > 250 | 6 | 0.78 | 0.71–0.84 | 0% |
| Location |  |  |  |  |
| USA | 9 | 0.72 | 0.65–0.81 | 0% |
| Others | 4 | 0.79 | 0.71–0.87 | 39.70% |
| Smoking |  |  |  |  |
| Never smoking | 5 | 0.47 | 0.35–0.63 | 0% |
| Ever smoking | 4 | 0.9 | 0.67–1.21 | 0% |
| Adjustment for smoking |  |  |  |  |
| Yes | 8 | 0.67 | 0.57–0.79 | 0% |
| No | 5 | 0.79 | 0.72–0.86 | 0% |
| Adjustment for age |  |  |  |  |
| Yes | 9 | 0.77 | 0.71–0.84 | 0% |
| No | 4 | 0.65 | 0.52–0.81 | 0% |
| Adjustment for BMI |  |  |  |  |
| Yes | 5 | 0.66 | 0.53–0.81 | 0% |
| No | 8 | 0.76 | 0.70–0.82 | 0% |

Notes: No, number; RR, Relative risk; CI: Confidence interval; BMI, Body Mass Index

Table S25. Methodological quality of included reviews assessed by AMSTAR-2

| Author, year | Include PICO elements | Predefine methods and explain deviations | Justify study design selection | Comprehensive search | Dual study selection | Dual extraction | List exclusions with reasons | Describe studies |
| --- | --- | --- | --- | --- | --- | --- | --- | --- |
| Nassour 2023^[1]^ | Yes | Yes | Yes | Partial Yes | Yes | Yes | Yes | Partial Yes |
| Connaughton 2022^[2]^ | Yes | Partial Yes | Yes | Partial Yes | Yes | Yes | Partial Yes | Partial Yes |
| Zhao 2019^[3]^ | Yes | Yes | Yes | Partial Yes | Yes | Yes | Yes | Yes |
| Dai 2016^[4]^ | Yes | Partial Yes | Yes | Partial Yes | Yes | Yes | No | Partial Yes |
| Bayne 2018^[5]^ | Yes | Partial Yes | Yes | Partial Yes | Yes | Yes | Partial Yes | Partial Yes |
| Yu 2018^[6]^ | Yes | Partial Yes | Yes | Partial Yes | Yes | Yes | Partial Yes | Partial Yes |
| Goyal 2021^[7]^ | Yes | Partial Yes | Yes | Partial Yes | Yes | Yes | Partial Yes | Yes |
| Yan 2014^[8]^ | Yes | Partial Yes | Yes | Partial Yes | Yes | Yes | Partial Yes | Yes |
| Feng 2021^[9]^ | Yes | Yes | Yes | Partial Yes | Yes | Yes | No | Yes |
| Zhang 2022^[10]^ | Yes | Yes | Yes | Partial Yes | Yes | Yes | No | Yes |
| Robertson 2022^[11]^ | Yes | Partial Yes | Yes | Partial Yes | No | No | Partial Yes | Yes |
| Ahmadinezhad 2022^[12]^ | Yes | No | Yes | Partial Yes | Yes | Yes | No | Yes |
| Shang 2015^[13]^ | Yes | No | Yes | Partial Yes | Yes | Yes | Partial Yes | Partial Yes |
| Allen 2023^[14]^ | Yes | Partial Yes | Yes | Partial Yes | Yes | Yes | Partial Yes | Partial Yes |
| Onishi 2013^[15]^ | Yes | Partial Yes | No | Partial Yes | Yes | No | Partial Yes | Yes |
| Wang 2020^[16]^ | Yes | Partial Yes | Yes | Partial Yes | Yes | Yes | Partial Yes | Partial Yes |
| Vaengebjerg 2020^[17]^ | Yes | Partial Yes | Yes | Partial Yes | Yes | Yes | Partial Yes | Yes |
| Yu 2024^[18]^ | Yes | Yes | Yes | Partial Yes | Yes | Yes | Partial Yes | Yes |
| Zhang 2021^[19]^ | Yes | Partial Yes | Yes | Partial Yes | Yes | Yes | Partial Yes | Partial Yes |
| Ma 2021^[20]^ | Yes | Partial Yes | Yes | Partial Yes | Yes | Yes | No | Partial Yes |
| Li 2022^[21]^ | Yes | Yes | Yes | Partial Yes | Yes | Yes | Yes | Yes |
| Yu 2023^[22]^ | Yes | Yes | Yes | Partial Yes | Yes | Yes | Partial Yes | Yes |
| Crippa 2018[^23]^ | Yes | No | Yes | No | Yes | Yes | Yes | Partial Yes |
| Yao 2014^[24]^ | Yes | No | Yes | Partial Yes | Yes | Yes | Partial Yes | Yes |
| Lin 2019^[25]^ | Yes | No | Yes | Partial Yes | Yes | Yes | Yes | Partial Yes |
| Wang 2019^[26]^ | Yes | No | No | Partial Yes | Yes | Yes | Partial Yes | Partial Yes |
| Dianatinasab 2022^[27]^ | Yes | Yes | Yes | Partial Yes | Yes | Yes | No | Yes |
| Hong 2018^[28]^ | Yes | No | Yes | Partial Yes | Yes | Yes | No | Partial Yes |
| Zheng 2024^[29]^ | Yes | No | Yes | Partial Yes | Yes | Yes | No | Yes |
| Baladia 2024^[30]^ | Yes | Partial Yes | Yes | Partial Yes | Yes | Yes | Yes | Yes |
| Long 2022^[31]^ | Yes | Yes | Yes | Partial Yes | Yes | Yes | Partial Yes | Yes |
| Lao 2021^[32]^ | Yes | Yes | Yes | Partial Yes | Yes | Yes | Yes | Yes |
| Bermej 2019^[33]^ | Yes | Yes | Yes | Partial Yes | Yes | Yes | Partial Yes | Yes |
| Li 2013^[34]^ | Yes | Partial Yes | Yes | No | Yes | Yes | No | Yes |
| Dai 2019^[35]^ | Yes | No | Yes | Partial Yes | Yes | Yes | Partial Yes | Partial Yes |
| Wu 2020^[36]^ | Yes | Yes | Yes | Partial Yes | Yes | Yes | Partial Yes | Partial Yes |
| Weng 2017^[37]^ | Yes | No | Yes | Partial Yes | Yes | Yes | Partial Yes | Yes |
| Dai 2024^[38]^ | Yes | Yes | Yes | Partial Yes | Yes | Yes | Partial Yes | Yes |
| Boyle 2014^[39]^ | Yes | No | Yes | Yes | No | No | Partial Yes | Partial Yes |
| Park 2017^[40]^ | Yes | No | Yes | Partial Yes | Yes | No | Partial Yes | Partial Yes |
| Seyyedsalehi 2021^[41]^ | Yes | No | Yes | Partial Yes | Yes | Yes | Partial Yes | Partial Yes |
| Li 2021^[42]^ | Yes | Partial Yes | Yes | Partial Yes | Yes | Yes | Partial Yes | Yes |
| Mofrad 2021^[43]^ | Yes | Yes | Yes | Partial Yes | Yes | Yes | Partial Yes | Yes |
| Filho 2023^[44]^ | Yes | Yes | Yes | Yes | Yes | Yes | Partial Yes | Yes |
| Fan 2021^[45]^ | Yes | Yes | Yes | Partial Yes | Yes | Yes | Yes | Yes |
| Tang 2018^[46]^ | Yes | No | Yes | Partial Yes | Yes | Yes | Partial Yes | Partial Yes |
| Xie 2020^[47]^ | Yes | No | Yes | Partial Yes | Yes | Yes | Partial Yes | Yes |
| Zhang 2021^[48]^ | Yes | No | Yes | Partial Yes | Yes | Yes | Partial Yes | Partial Yes |
| Tang 2012^[49]^ | Yes | Partial Yes | Yes | Partial Yes | Yes | Yes | Partial Yes | Partial Yes |
| Xiang 2021^[50]^ | Yes | Yes | Yes | Partial Yes | Yes | Yes | Partial Yes | Yes |
| BOSETTI 2013^[51]^ | Yes | No | Yes | Partial Yes | Yes | Yes | Partial Yes | Yes |
| Zhang 2013^[52]^ | Yes | No | No | No | Yes | Yes | Partial Yes | Partial Yes |
| Xu 2020^[53]^ | Yes | No | Yes | No | Yes | Yes | No | No |
| Spiazzi 2024^[54]^ | Yes | Yes | Yes | Yes | Yes | Yes | Partial Yes | Yes |
| Symvoulidis 2023^[55]^ | Yes | Yes | Yes | Partial Yes | Yes | Yes | Yes | Yes |
| Bommareddy 2022^[56]^ | Yes | Yes | Yes | Partial Yes | Yes | Yes | Partial Yes | Yes |
| Shi 2024^[57]^ | Yes | Yes | Yes | Partial Yes | Yes | Yes | Partial Yes | Yes |
| Li 2024^[58]^ | Yes | Yes | Yes | Partial Yes | Yes | Yes | Partial Yes | Yes |
| Liang 2016^[59]^ | Yes | No | No | Partial Yes | Yes | Yes | Partial Yes | Partial Yes |
| Villanueva 2003^[60]^ | Yes | No | Yes | Partial Yes | No | No | Partial Yes | Yes |
| Yan 2018^[61]^ | Yes | No | Yes | Partial Yes | Yes | Yes | Partial Yes | Yes |
| Zhang 2020^[62]^ | Yes | No | Yes | Partial Yes | Yes | Yes | Partial Yes | Yes |
| Zhao 2022^[63]^ | Yes | Partial Yes | Yes | Partial Yes | Yes | Yes | Partial Yes | Yes |
| Keimling 2014^[64]^ | Yes | Partial Yes | Yes | Partial Yes | Yes | Yes | Partial Yes | Partial Yes |
| Arafa 2022^[65]^ | Yes | No | Yes | Partial Yes | No | No | Yes | Yes |
| Kronstedt 2024^[66]^ | Yes | Yes | Yes | Partial Yes | Yes | Yes | Yes | Yes |
| Vlaanderen 2014^[67]^ | Yes | Partial Yes | Yes | Partial Yes | No | Yes | Yes | Partial Yes |
| Guha 2010^[68]^ | Yes | No | No | No | Yes | Yes | No | Partial Yes |
| Boniol 2017^[69]^ | Yes | No | Yes | Partial Yes | Yes | Yes | Yes | Partial Yes |
| Harling 2010^[70]^ | Yes | Partial Yes | No | Partial Yes | Yes | Yes | Partial Yes | Partial Yes |
| DeBono 2023^[71]^ | Yes | Yes | Yes | Partial Yes | Yes | Yes | Partial Yes | Yes |
| Boffetta 2001^[72]^ | Yes | No | No | No | No | No | No | Partial Yes |
| Alif 2022^[73]^ | Yes | Yes | No | Yes | Yes | Yes | No | Yes |
| Fu 1995^[74]^ | Yes | No | No | No | No | No | No | Partial Yes |
| Collatuzzo 2024^[75]^ | Yes | Yes | Yes | Partial Yes | Yes | Yes | Partial Yes | Partial Yes |
| Seyyedsalehi 2024^[76]^ | Yes | Partial Yes | No | Partial Yes | Yes | Yes | Partial Yes | Yes |
| Franco 2023^[77]^ | Yes | No | No | Partial Yes | Yes | Yes | No | Yes |
| Mundt 2018^[78]^ | Yes | No | Yes | No | No | No | Yes | Yes |
| Turati 2014^[79]^ | Yes | No | Yes | Partial Yes | Yes | Yes | Partial Yes | Yes |
| Collins 1998^[80]^ | Yes | No | No | No | No | No | No | Yes |
| Cui 2023^[81]^ | Yes | No | No | Partial Yes | No | Yes | Partial Yes | Partial Yes |
| Bai 2017^[82]^ | Yes | No | Yes | Partial Yes | Yes | Yes | Partial Yes | Partial Yes |
| Shi 2018^[83]^ | Yes | Partial Yes | Yes | Partial Yes | Yes | Yes | Partial Yes | Yes |
| Li 2022^[84]^ | Yes | Partial Yes | Yes | Partial Yes | Yes | Yes | Partial Yes | Yes |

P,Patient / Population / Problem; I,Intervention; C,Comparison / Control; O, Outcome

Table S25. Methodological quality of included reviews assessed by AMSTAR-2. (continue)

| Author, year | Use proper bias assessment | Report funding sources | Use proper methods | Assess bias impact | Consider bias in interpretation | Explain heterogeneity | Assess and discuss publication bias | Report conflicts and funding | Quality |
| --- | --- | --- | --- | --- | --- | --- | --- | --- | --- |
| Nassour 2023^[1]^ | Partial Yes | No | No | No | Yes | Yes | No | Yes | Critically Low |
| Connaughton 2022^[2]^ | Yes | Yes | Yes | No | No | No | No | Yes | Critically Low |
| Zhao 2019^[3]^ | Yes | No | Yes | No | Yes | Yes | Yes | Yes | High |
| Dai 2016^[4]^ | Yes | No | Yes | Yes | Yes | Yes | Yes | Yes | low |
| Bayne 2018^[5]^ | Partial Yes | No | Yes | Yes | Yes | Yes | Yes | Yes | moderate |
| Yu 2018^[6]^ | No | No | Yes | No | No | Yes | Yes | Yes | Critically Low |
| Goyal 2021^[7]^ | Partial Yes | No | Yes | Yes | Yes | Yes | Yes | Yes | moderate |
| Yan 2014^[8]^ | No | No | Yes | No | No | Yes | Yes | Yes | Critically Low |
| Feng 2021^[9]^ | Yes | No | Yes | Yes | Yes | Yes | Yes | Yes | low |
| Zhang 2022^[10]^ | Yes | No | Yes | Yes | Yes | Yes | Yes | Yes | low |
| Robertson 2022^[11]^ | Yes | No | Yes | No | Yes | Yes | Yes | Yes | moderate |
| Ahmadinezhad 2022^[12]^ | Yes | No | Yes | No | No | Yes | Yes | Yes | Critically Low |
| Shang 2015^[13]^ | Yes | No | Yes | No | Yes | Yes | Yes | Yes | low |
| Allen 2023^[14]^ | Yes | No | Yes | No | Yes | Yes | Yes | Yes | low |
| Onishi 2013^[15]^ | Partial Yes | No | Yes | Yes | Yes | Yes | Yes | Yes | moderate |
| Wang 2020^[16]^ | Yes | No | Yes | Yes | Yes | Yes | Yes | Yes | moderate |
| Vaengebjerg 2020^[17]^ | Yes | No | Yes | Yes | Yes | Yes | Yes | Yes | moderate |
| Yu 2024^[18]^ | Yes | No | Yes | Yes | Yes | Yes | Yes | Yes | moderate |
| Zhang 2021^[19]^ | Yes | No | Yes | Yes | Yes | Yes | Yes | Yes | moderate |
| Ma 2021^[20]^ | Partial Yes | No | Yes | Yes | Yes | Yes | Yes | Yes | low |
| Li 2022^[21]^ | Yes | No | Yes | Yes | Yes | Yes | Yes | Yes | high |
| Yu 2023^[22]^ | Yes | No | Yes | Yes | Yes | Yes | Yes | Yes | moderate |
| Crippa 2018[^23]^ | No | No | Yes | No | No | Yes | Yes | Yes | Critically Low |
| Yao 2014^[24]^ | No | No | Yes | No | No | Yes | Yes | Yes | Critically Low |
| Lin 2019^[25]^ | No | No | Yes | No | No | Yes | Yes | Yes | Critically Low |
| Wang 2019^[26]^ | No | No | Yes | No | No | Yes | Yes | Yes | Critically Low |
| Dianatinasab 2022^[27]^ | Yes | No | Yes | No | Yes | Yes | Yes | Yes | low |
| Hong 2018^[28]^ | Yes | No | Yes | No | Yes | Yes | Yes | Yes | Critically Low |
| Zheng 2024^[29]^ | Yes | No | Yes | Yes | Yes | Yes | Yes | Yes | Critically Low |
| Baladia 2024^[30]^ | Yes | No | Yes | Yes | Yes | Yes | Yes | Yes | moderate |
| Long 2022^[31]^ | Yes | No | Yes | No | Yes | Yes | Yes | Yes | moderate |
| Lao 2021^[32]^ | Yes | No | Yes | Yes | Yes | Yes | Yes | Yes | high |
| Bermej 2019^[33]^ | Yes | No | Yes | Yes | Yes | Yes | Yes | Yes | moderate |
| Li 2013^[34]^ | No | No | Yes | No | No | Yes | Yes | Yes | Critically Low |
| Dai 2019^[35]^ | No | No | Yes | Yes | Yes | Yes | Yes | Yes | Critically Low |
| Wu 2020^[36]^ | Partial Yes | No | Yes | Yes | Yes | Yes | Yes | Yes | moderate |
| Weng 2017^[37]^ | Partial Yes | No | Yes | Yes | Yes | Yes | Yes | Yes | low |
| Dai 2024^[38]^ | Yes | No | Yes | Yes | Yes | Yes | Yes | Yes | moderate |
| Boyle 2014^[39]^ | No | No | Yes | No | No | Yes | Yes | Yes | Critically Low |
| Park 2017^[40]^ | Yes | No | Yes | No | No | Yes | Yes | Yes | low |
| Seyyedsalehi 2021^[41]^ | Yes | No | Yes | Yes | Yes | Yes | Yes | Yes | low |
| Li 2021^[42]^ | Yes | No | Yes | Yes | Yes | Yes | Yes | Yes | moderate |
| Mofrad 2021^[43]^ | Yes | No | Yes | Yes | Yes | Yes | Yes | Yes | moderate |
| Filho 2023^[44]^ | Yes | No | Yes | Yes | Yes | Yes | Yes | Yes | High |
| Fan 2021^[45]^ | Yes | No | Yes | Yes | Yes | Yes | Yes | Yes | High |
| Tang 2018^[46]^ | Yes | Yes | Yes | Yes | Yes | Yes | Yes | Yes | low |
| Xie 2020^[47]^ | Yes | No | Yes | Yes | Yes | Yes | Yes | Yes | low |
| Zhang 2021^[48]^ | No | No | Yes | No | No | Yes | Yes | Yes | Critically Low |
| Tang 2012^[49]^ | Partial Yes | No | Yes | Yes | Yes | Yes | Yes | Yes | moderate |
| Xiang 2021^[50]^ | Yes | No | Yes | No | Yes | Yes | No | Yes | low |
| BOSETTI 2013^[51]^ | No | No | Yes | No | No | Yes | Yes | Yes | Critically Low |
| Zhang 2013^[52]^ | No | No | Yes | No | No | Yes | Yes | Yes | Critically Low |
| Xu 2020^[53]^ | No | No | Yes | No | No | No | No | Yes | Critically Low |
| Spiazzi 2024^[54]^ | Yes | Yes | Yes | Yes | Yes | Yes | Yes | Yes | High |
| Symvoulidis 2023^[55]^ | Yes | No | Yes | No | Yes | Yes | Yes | Yes | High |
| Bommareddy 2022^[56]^ | Yes | No | Yes | No | Yes | Yes | No | Yes | low |
| Shi 2024^[57]^ | Yes | No | Yes | Yes | Yes | Yes | Yes | Yes | moderate |
| Li 2024^[58]^ | Yes | No | Yes | Yes | Yes | Yes | Yes | Yes | moderate |
| Liang 2016^[59]^ | Yes | No | Yes | Yes | Yes | Yes | Yes | Yes | low |
| Villanueva 2003^[60]^ | No | No | Yes | No | No | Yes | Yes | Yes | Critically Low |
| Yan 2018^[61]^ | Yes | No | Yes | Yes | Yes | Yes | Yes | Yes | low |
| Zhang 2020^[62]^ | Yes | No | Yes | Yes | Yes | Yes | Yes | Yes | low |
| Zhao 2022^[63]^ | Yes | No | Yes | No | Yes | Yes | Yes | Yes | moderate |
| Keimling 2014^[64]^ | No | No | Yes | No | No | Yes | Yes | Yes | Critically Low |
| Arafa 2022^[65]^ | Yes | No | Yes | Yes | Yes | Yes | Yes | Yes | low |
| Kronstedt 2024^[66]^ | No | No | Yes | Yes | Yes | Yes | No | Yes | Critically Low |
| Vlaanderen 2014^[67]^ | No | No | Yes | No | Yes | Yes | Yes | Yes | low |
| Guha 2010^[68]^ | No | No | Yes | No | No | Yes | Yes | Yes | Critically Low |
| Boniol 2017^[69]^ | No | No | Yes | No | Yes | Yes | Yes | Yes | Critically Low |
| Harling 2010^[70]^ | Partial Yes | No | Yes | Yes | Yes | Yes | Yes | Yes | moderate |
| DeBono 2023^[71]^ | Yes | No | Yes | Yes | Yes | Yes | No | Yes | low |
| Boffetta 2001^[72]^ | No | No | No | No | Yes | Yes | Yes | Yes | Critically Low |
| Alif 2022^[73]^ | Partial Yes | No | Yes | Yes | Yes | Yes | Yes | Yes | low |
| Fu 1995^[74]^ | No | No | Yes | No | Yes | Yes | No | Yes | Critically Low |
| Collatuzzo 2024^[75]^ | Partial Yes | No | Yes | Yes | Yes | Yes | Yes | Yes | moderate |
| Seyyedsalehi 2024^[76]^ | Yes | No | No | Yes | Yes | Yes | Yes | Yes | low |
| Franco 2023^[77]^ | Partial Yes | No | Yes | Yes | Yes | Yes | Yes | Yes | Critically Low |
| Mundt 2018^[78]^ | Yes | No | Yes | Yes | Yes | Yes | Yes | Yes | Critically Low |
| Turati 2014^[79]^ | Partial Yes | No | Yes | No | Yes | Yes | Yes | Yes | low |
| Collins 1998^[80]^ | No | No | Yes | No | No | Yes | Yes | Yes | Critically Low |
| Cui 2023^[81]^ | Yes | No | Yes | Yes | Yes | Yes | Yes | Yes | low |
| Bai 2017^[82]^ | Yes | No | Yes | Yes | Yes | Yes | Yes | Yes | low |
| Shi 2018^[83]^ | Yes | No | Yes | Yes | Yes | Yes | Yes | Yes | moderate |
| Li 2022^[84]^ | Yes | No | Yes | Yes | Yes | Yes | Yes | Yes | moderate |

P,Patient / Population / Problem; I,Intervention; C,Comparison / Control; O, Outcome

References

[1] NASSOUR A J, JAIN A, HUI N, et al.: Relative Risk of Bladder and Kidney Cancer in Lynch Syndrome: Systematic Review and Meta-Analysis. Cancers (Basel). 2023; 15.

[2] CONNAUGHTON M, DABAGH M: Association of Hypertension and Organ-Specific Cancer: A Meta-Analysis. Healthcare (Basel). 2022; 10.

[3] ZHAO S K, XIE Q, YANG R D, et al.: High prevalence of secondary bladder cancer in men on radiotherapy for prostate cancer: evidence from a meta-analysis. Cancer Management and Research. 2019; 11:587-98.

[4] DAI X, FANG X, MA Y, et al.: Benign Prostatic Hyperplasia and the Risk of Prostate Cancer and Bladder Cancer: A Meta-Analysis of Observational Studies. Medicine (Baltimore). 2016; 95:e3493.

[5] BAYNE C E, FARAH D, HERBST K W, et al.: Role of urinary tract infection in bladder cancer: a systematic review and meta-analysis. World J Urol. 2018; 36:1181-90.

[6] YU Z, YUE W, JIUZHI L, et al.: The risk of bladder cancer in patients with urinary calculi: a meta-analysis. Urolithiasis. 2018; 46:573-9.

[7] GOYAL A, O'LEARY D, GOYAL K, et al.: Cutaneous T-cell lymphoma is associated with increased risk of lymphoma, melanoma, lung cancer, and bladder cancer. J Am Acad Dermatol. 2021; 85:1418-28.

[8] YAN L, CHEN P, CHEN E Z, et al.: Risk of bladder cancer in renal transplant recipients: a meta-analysis. Br J Cancer. 2014; 110:1871-7.

[9] FENG S, SHAO Z, JU L, et al.: Atopy, asthma, and risk of bladder cancer: Systematic review and meta-analysis of cohort studies. European Journal of Inflammation. 2021; 19.

[10] ZHANG M, WANG Y Z, WANG Y T, et al.: Association Between Systemic Lupus Erythematosus and Cancer Morbidity and Mortality: Findings From Cohort Studies. Frontiers in Oncology. 2022; 12.

[11] ROBERTSON D, NG S K, BAADE P D, et al.: Risk of extracolonic second primary cancers following a primary colorectal cancer: a systematic review and meta-analysis. Int J Colorectal Dis. 2022; 37:541-51.

[12] AHMADINEZHAD M, ARSHADI M, HESARI E, et al.: The relationship between metabolic syndrome and its components with bladder cancer: a systematic review and meta-analysis of cohort studies. Epidemiol Health. 2022; 44:e2022050.

[13] SHANG W F, NING Y, XU X, et al.: Incidence of Cancer in ANCA-Associated Vasculitis: A Meta-Analysis of Observational Studies. Plos One. 2015; 10.

[14] ALLEN I, HASSAN H, SOFIANOPOULOU E, et al.: Risks of second non-breast primaries following breast cancer in women: a systematic review and meta-analysis. Breast Cancer Res. 2023; 25:18.

[15] ONISHI A, SUGIYAMA D, KUMAGAI S, et al.: Cancer incidence in systemic sclerosis: meta-analysis of population-based cohort studies. Arthritis Rheum. 2013; 65:1913-21.

[16] WANG Y H, LI J Q, SHI J F, et al.: Depression and anxiety in relation to cancer incidence and mortality: a systematic review and meta-analysis of cohort studies. Mol Psychiatry. 2020; 25:1487-99.

[17] VAENGEBJERG S, SKOV L, EGEBERG A, et al.: Prevalence, Incidence, and Risk of Cancer in Patients With Psoriasis and Psoriatic Arthritis: A Systematic Review and Meta-analysis. JAMA Dermatol. 2020; 156:421-9.

[18] YU L L, YAN Y C, LIU W J, et al.: Association of ankylosing spondylitis with the risk of cancer: a meta-analysis of cohort studies. Rheumatology. 2024.

[19] ZHANG C, LIU S Z, PENG L, et al.: Does inflammatory bowel disease increase the risk of lower urinary tract tumors: a meta-analysis. Translational Andrology and Urology. 2021; 10.

[20] MA Y C, HUANG Z L, JIAN Z Y, et al.: The association between hepatitis C virus infection and renal cell cancer, prostate cancer, and bladder cancer: a systematic review and meta-analysis. Sci Rep. 2021; 11.

[21] LI W Q, WANG S M, HE Y H, et al.: Is periodontal disease a risk indicator for urogenital cancer? A systematic review and meta-analysis of cohort studies. Frontiers in Oncology. 2022; 12.

[22] YU J, LI H, LIU Z, et al.: Meat Intake and the Risk of Bladder Cancer: A Systematic Review and Meta-Analysis of Observational Studies. Nutr Cancer. 2023; 75:825-45.

[23] CRIPPA A, LARSSON S C, DISCACCIATI A, et al.: Red and processed meat consumption and risk of bladder cancer: a dose-response meta-analysis of epidemiological studies. Eur J Nutr. 2018; 57:689-701.

[24] YAO B, YAN Y, YE X, et al.: Intake of fruit and vegetables and risk of bladder cancer: a dose-response meta-analysis of observational studies. Cancer Causes Control. 2014; 25:1645-58.

[25] LIN J H, CHEN S J, LIU H, et al.: Vitamin E consumption and the risk of bladder cancer: A meta-analysis of prospective studies. International Journal for Vitamin and Nutrition Research. 2019; 89(3-4):168-75.

[26] WANG J, WANG C: Dietary fat intake and risk of bladder cancer: Evidence from a meta-analysis of observational studies. Cell Mol Biol (Noisy-le-grand). 2019; 65:5-9.

[27] DIANATINASAB M, FOROZANI E, AKBARI A, et al.: Dietary patterns and risk of bladder cancer: a systematic review and meta-analysis. BMC Public Health. 2022; 22:73.

[28] HONG X W, XU Q C, LAN K J, et al.: The Effect of Daily Fluid Management and Beverages Consumption on the Risk of Bladder Cancer: A Meta-analysis of Observational Study. Nutrition and Cancer-an International Journal. 2018; 70:1217-27.

[29] ZHENG S C, YAN J L, WANG J X, et al.: Unveiling the Effects of Cruciferous Vegetable Intake on Different Cancers: A Systematic Review and Dose-Response Meta-analysis. Nutrition Reviews. 2024.

[30] BALADIA E, MOñINO M, PLEGUEZUELOS E, et al.: Broccoli Consumption and Risk of Cancer: An Updated Systematic Review and Meta-Analysis of Observational Studies. Nutrients. 2024; 16.

[31] LONG T, LIU K, LONG J, et al.: Dietary glycemic index, glycemic load and cancer risk: a meta-analysis of prospective cohort studies. Eur J Nutr. 2022; 61:2115-27.

[32] LAO Y F, LI X L, HE L J, et al.: Association Between Alcohol Consumption and Risk of Bladder Cancer: A Dose-Response Meta-Analysis of Prospective Cohort Studies. Frontiers in Oncology. 2021; 11.

[33] BERMEJO L M, LóPEZ-PLAZA B, SANTURINO C, et al.: Milk and Dairy Product Consumption and Bladder Cancer Risk: A Systematic Review and Meta-Analysis of Observational Studies. Adv Nutr. 2019; 10:S224-s38.

[34] LI F, ZHOU Y, HU R T, et al.: Egg Consumption and Risk of Bladder Cancer: A Meta-Analysis. Nutrition and Cancer-an International Journal. 2013; 65:538-46.

[35] DAI Z W, CAI K D, LI F R, et al.: Association between coffee consumption and risk of bladder cancer in a meta-analysis of 16 prospective studies. Nutr Metab (Lond). 2019; 16:66.

[36] WU S, LIU Y, MICHALEK J E, et al.: Carotenoid Intake and Circulating Carotenoids Are Inversely Associated with the Risk of Bladder Cancer: A Dose-Response Meta-analysis. Adv Nutr. 2020; 11:630-43.

[37] WENG H, ZENG X T, LI S, et al.: Tea consumption and risk of bladder cancer: A dose-response meta-analysis. Frontiers in Physiology. 2017; 7(JAN) (no pagination).

[38] DAI Y N, YU E Y W, ZEEGERS M P, et al.: The Association between Dietary Inflammatory Potential and Urologic Cancers: A Meta-analysis. Advances in Nutrition. 2024; 15.

[39] BOYLE P, KOECHLIN A, AUTIER P: Sweetened carbonated beverage consumption and cancer risk: meta-analysis and review. Eur J Cancer Prev. 2014; 23:481-90.

[40] PARK S J, MYUNG S K, LEE Y, et al.: Effects of Vitamin and Antioxidant Supplements in Prevention of Bladder Cancer: a Meta-Analysis of Randomized Controlled Trials. J Korean Med Sci. 2017; 32:628-35.

[41] SEYYEDSALEHI M S, MOHEBBI E, SASANFAR B, et al.: Dietary N-nitroso compounds intake and bladder cancer risk: A systematic review and meta-analysis. Nitric Oxide. 2021; 115:1-7.

[42] LI Y, GUO L, HE K, et al.: Consumption of sugar-sweetened beverages and fruit juice and human cancer: A systematic review and dose-response meta-analysis of observational studies. Journal of Cancer. 2021; 12(10):3077-88.

[43] DAROOGHEGI MOFRAD M, MOZAFFARI H, ASKARI M R, et al.: Potato Consumption and Risk of Site-Specific Cancers in Adults: A Systematic Review and Dose-Response Meta-Analysis of Observational Studies. Adv Nutr. 2021; 12:1705-22.

[44] FILHO A M, TURNER M C, WARNAKULASURIYA S, et al.: The carcinogenicity of opium consumption: a systematic review and meta-analysis. European Journal of Epidemiology. 2023; 38:373-89.

[45] FAN B, MOHAMMED A, HUANG Y B, et al.: Can Aspirin Use Be Associated With the Risk or Prognosis of Bladder Cancer? A Case-Control Study and Meta-analytic Assessment. Frontiers in Oncology. 2021; 11.

[46] TANG H, SHI W, FU S, et al.: Pioglitazone and bladder cancer risk: a systematic review and meta-analysis. Cancer Med. 2018; 7:1070-80.

[47] XIE Y, XU P, WANG M, et al.: Antihypertensive medications are associated with the risk of kidney and bladder cancer: a systematic review and meta-analysis. Aging (Albany NY). 2020; 12:1545-62.

[48] ZHANG K, BAI P, DAI H, et al.: Metformin and risk of cancer among patients with type 2 diabetes mellitus: A systematic review and meta-analysis. Primary Care Diabetes. 2021; 15(1):52-8.

[49] TANG X, YANG L, HE Z, et al.: Insulin Glargine and Cancer Risk in Patients with Diabetes: A Meta-Analysis. PLoS ONE. 2012; 7(12) (no pagination).

[50] XIANG P, DU Z, HAO Y X, et al.: Impact of Androgen Suppression Therapy on the Risk and Prognosis of Bladder Cancer: A Systematic Review and Meta-Analysis. Frontiers in Oncology. 2021; 11.

[51] BOSETTI C, ROSATO V, BUNIATO D, et al.: Cancer risk for patients using thiazolidinediones for type 2 diabetes: a meta-analysis. Oncologist. 2013; 18:148-56.

[52] ZHANG H, JIANG D, LI X: Use of Nonsteroidal Anti-Inflammatory Drugs and Bladder Cancer Risk: A Meta-Analysis of Epidemiologic Studies. PLoS ONE. 2013; 8(7) (no pagination).

[53] XU X, MO Q W, SHEN H X, et al.: Reproductive and hormonal factors and bladder cancer risk: a prospective study and meta-analysis. Aging-Us. 2020; 12:14691-8.

[54] XU B, KANG B, LI S, et al.: Sodium-glucose cotransporter 2 inhibitors and cancer: a systematic review and meta-analysis. Journal of Endocrinological Investigation. 2024; 47(10):2421-36.

[55] SYMVOULIDIS P, TSIOUTIS C, ZAMBOGLOU C, et al.: The Effect of Statins on the Incidence and Prognosis of Bladder Cancer: A Systematic Review and Meta-Analysis. Curr Oncol. 2023; 30:6648-65.

[56] BOMMAREDDY K, HAMADE H, LOPEZ-OLIVO M A, et al.: Association of Spironolactone Use With Risk of Cancer: A Systematic Review and Meta-analysis. JAMA Dermatol. 2022; 158:275-82.

[57] SHI J, ZHANG K, XIAO T, et al.: Exposure to disinfection by-products and risk of cancer: A systematic review and dose-response meta-analysis. Ecotoxicology and Environmental Safety. 2024; 270(no pagination).

[58] LI J, DENG Z, SOERENSEN S J C, et al.: Ambient air pollution and urological cancer risk: A systematic review and meta-analysis of epidemiological evidence. Nat Commun. 2024; 15:5116.

[59] LIANG Z, WANG X, XIE B, et al.: Pesticide exposure and risk of bladder cancer: A meta-analysis. Oncotarget. 2016; 7:66959-69.

[60] VILLANUEVA C M, FERNáNDEZ F, MALATS N, et al.: Meta-analysis of studies on individual consumption of chlorinated drinking water and bladder cancer. J Epidemiol Community Health. 2003; 57:166-73.

[61] YAN H Q, YING Y F, XIE H Y, et al.: Secondhand smoking increases bladder cancer risk in nonsmoking population: a meta-analysis. Cancer Management and Research. 2018; 10:3781-91.

[62] ZHANG Y B, PAN X F, CHEN J X, et al.: Combined lifestyle factors, incident cancer, and cancer mortality: a systematic review and meta-analysis of prospective cohort studies. British Journal of Cancer. 2020; 122:1085-93.

[63] ZHAO X, WANG Y, LIANG C: Cigarette smoking and risk of bladder cancer: a dose-response meta-analysis. Int Urol Nephrol. 2022; 54:1169-85.

[64] KEIMLING M, BEHRENS G, SCHMID D, et al.: The association between physical activity and bladder cancer: systematic review and meta-analysis. Br J Cancer. 2014; 110:1862-70.

[65] ARAFA A, EWIS A, ESHAK E: Chronic exposure to nitrate in drinking water and the risk of bladder cancer: a meta-analysis of epidemiological evidence. Public Health. 2022; 203:123-9.

[66] KRONSTEDT S, CATHEY J, CHIU C B, et al.: Exposures and Bladder Cancer Risk Among Military Veterans: A Systematic Review and Meta-analysis. Urology. 2024.

[67] VLAANDEREN J, STRAIF K, RUDER A, et al.: Tetrachloroethylene exposure and bladder cancer risk: A meta-analysis of dry-cleaning-worker studies. Environmental Health Perspectives. 2014; 122(7):661-6.

[68] GUHA N, STEENLAND N K, MERLETTI F, et al.: Bladder cancer risk in painters: a meta-analysis. Occup Environ Med. 2010; 67:568-73.

[69] BONIOL M, KOECHLIN A, BOYLE P: Meta-analysis of occupational exposures in the rubber manufacturing industry and risk of cancer. Int J Epidemiol. 2017; 46:1940-7.

[70] HARLING M, SCHABLON A, SCHEDLBAUER G, et al.: Bladder cancer among hairdressers: a meta-analysis. Occup Environ Med. 2010; 67:351-8.

[71] DEBONO N L, DANIELS R D, BEANE FREEMAN L E, et al.: Firefighting and Cancer: A Meta-analysis of Cohort Studies in the Context of Cancer Hazard Identification. Saf Health Work. 2023; 14:141-52.

[72] BOFFETTA P, SILVERMAN D T: A meta-analysis of bladder cancer and diesel exhaust exposure. Epidemiology. 2001; 12:125-30.

[73] ALIF S M, SIM M R, HO C, et al.: Cancer and mortality in coal mine workers: a systematic review and meta-analysis. Occupational and Environmental Medicine. 2022; 79:347-57.

[74] FU H, BOFFETTA P: Cancer and occupational exposure to inorganic lead compounds: a meta-analysis of published data. Occup Environ Med. 1995; 52:73-81.

[75] COLLATUZZO G, HAMDANI M, BOFFETTA P: Risk of bladder, kidney and prostate cancer from occupational exposure to welding fumes: a systematic review and meta-analysis. Int Arch Occup Environ Health. 2024; 97:221-30.

[76] SEYYEDSALEHI M S, BONETTI M, SHAH D, et al.: Occupational benzene exposure and risk of kidney and bladder cancers: a systematic review and meta-analysis. Eur J Cancer Prev. 2024.

[77] FRANCO N, GODONO A, CLARI M, et al.: Occupational asbestos exposure and urinary bladder cancer: a systematic review and meta-analysis. World J Urol. 2023; 41:1005-15.

[78] MUNDT K A, DELL L D, CRAWFORD L, et al.: Cancer Risk Associated With Exposure to Bitumen and Bitumen Fumes: An Updated Systematic Review and Meta-Analysis. J Occup Environ Med. 2018; 60:e6-e54.

[79] TURATI F, PELUCCHI C, GALEONE C, et al.: Personal hair dye use and bladder cancer: a meta-analysis. Ann Epidemiol. 2014; 24:151-9.

[80] COLLINS J J, ACQUAVELLA J F: Review and meta-analysis of studies of acrylonitrile workers. Scand J Work Environ Health. 1998; 24 Suppl 2:71-80.

[81] CUI H J, QU Y, ZHANG L, et al.: Epidemiological and genetic evidence for the relationship between ABO blood group and human cancer. International Journal of Cancer. 2023; 153:320-30.

[82] BAI Y J, WANG X M, YANG Y B, et al.: Parity and bladder cancer risk: a dose-response meta-analysis. Bmc Cancer. 2017; 17.

[83] SHI J, LENG W, ZHAO L, et al.: Tooth loss and cancer risk: a dose-response meta analysis of prospective cohort studies. Oncotarget. 2018; 9:15090-100.

[84] LI Y D, GAO L, GOU Y Q, et al.: Age of menarche and primary bladder cancer risk: A meta-analysis and systematic review. Urol Oncol. 2022; 40:346.e17-.e26.

TableS26 Risk Factors and Grade Classification of Bladder Cancer

| Risk factors | Assessed with | Outcomes | Included MA | No. of studies T/C/P | Risk of bias | Inconsistency | Indirectness | Imprecision | Publication bias | Plausible confounding | Magnitude of effect | Dose-response gradient | Quality |
| --- | --- | --- | --- | --- | --- | --- | --- | --- | --- | --- | --- | --- | --- |
| **Significant** |  |  |  |  |  |  |  |  |  |  |  |  |  |
| LS | With vs. without | risk of bladder cancer | Nassour 2023 | 4/4/0 | No serious risk | Serious inconsistency | No serious indirectness | No serious imprecision | NA | Would not reduce effect | Yes | No | low |
| Hypertension | DBP per 10 mmHg | risk of muscle-invasive bladder cancer (MIBC) | Connaughton 2022 | 7/7/0 | Serious risk | NA | No serious indirectness | Serious imprecision | NA | Would not reduce effect | No | Yes | very low |
| radiotherapy for prostate cancer | With vs. without | secondary bladder cancer in men | Zhao 2019 | 16/16/0 | No serious risk | Serious inconsistency | No serious indirectness | No serious imprecision | Undetected | Would not reduce effect | No | No | very low |
| BPH | With vs. without | The relative risk of bladder cancer in men | Dai 2016 | 6/4/2 | No serious risk | No serious inconsistency | No serious indirectness | No serious imprecision | Undetected | would not reduce effect | No | No | low |
| urinary tract infection | With vs. without | risk of bladder cancer | Bayne 2018 | 8/0/8 | Serious risk | Serious inconsistency | No serious indirectness | No serious imprecision | Undetected | Would not reduce effect | No | No | very low |
| urinary calculi | With vs. without | risk of bladder cancer | Zhang 2018 | 13/3/10 | Serious risk | Serious inconsistency | No serious indirectness | No serious imprecision | Strongly suspected | Would not reduce effect | No | No | very low |
| Cutaneous T-cell lymphoma | With vs. without | risk of bladder cancer | Goyal 2021 | 8/8/0 | No serious risk | No serious inconsistency | No serious indirectness | Serious imprecision | Undetected | Would not reduce effect | No | No | very low |
| renal transplant | With vs. without | risk of bladder cancer | Yan 2014 | 11/11/0 | Serious risk | Serious inconsistency | No serious indirectness | Serious imprecision | Undetected | Would not reduce effect | Yes | No | very low |
| overall atopy | With vs. without | risk of bladder cancer | Feng 2021 | 10/10/0 | No serious risk | Serious inconsistency | No serious indirectness | Serious imprecision | Undetected | Would not reduce effect | No | No | very low |
| asthma | With vs. without | risk of bladder cancer | Feng 2021 | 7/7/0 | No serious risk | Serious inconsistency | No serious indirectness | No serious imprecision | Undetected | Would not reduce effect | No | No | very low |
| Lupus Erythematosus | With vs. without | risk of bladder cancer | Zhang 2022 | 12/12/0 | No serious risk | Serious inconsistency | No serious indirectness | Serious imprecision | Strongly suspected | Would not reduce effect | No | No | very low |
| primary colorectal cancer | With vs. without | secondary bladder cance | Robertson 2022 | 7/7/0 | Serious risk | Serious inconsistency | No serious indirectness | Serious imprecision | Undetected | Would not reduce effect | No | No | very low |
| disinfection by-products | exposure vs. non-exposure | risk of bladder cancer | Shi 2024 | 11/1/10 | No serious risk | Serious inconsistency | No serious indirectness | No serious imprecision | Undetected | Would not reduce effect | No | No | very low |
| PM2.5 | 5 μg/m^3^ increment | risk of bladder cancer | Li 2024 | 8/6/2 | No serious risk | No serious inconsistency | No serious indirectness | No serious imprecision | Undetected | Would not reduce effect | No | Yes | moderate |
| NO2 | 10 μg/m^3^ increment | risk of bladder cancer | Li 2024 | 6/4/2 | No serious risk | No serious inconsistency | No serious indirectness | Serious imprecision | Undetected | Would not reduce effect | No | Yes | low |
| lifestyle factors | healthiest with the least healthy lifestyles | risk of bladder cancer | Zhang 2020 | 2/2/0 | No serious risk | No serious inconsistency | No serious indirectness | No serious imprecision | Strongly suspected | Would not reduce effect | No | No | very low |
| ABO blood group | A vs O | risk of bladder cancer | Cui 2023 | 5/0/5 | No serious risk | No serious inconsistency | No serious indirectness | Serious imprecision | Undetected | Would not reduce effect | No | No | very low |
| diabetes | With vs. without | risk of bladder cancer | Ahmadinezhad 2022 | 17/17/0 | No serious risk | Serious inconsistency | No serious indirectness | No serious imprecision | Undetected | Would not reduce effect | No | No | very low |
| metabolic syndrome | With vs. without | risk of bladder cancer | Ahmadinezhad 2022 | 2/2/0 | No serious risk | No serious inconsistency | No serious indirectness | Serious imprecision | Undetected | Would not reduce effect | No | No | very low |
| Parity | ever parity | risk of bladder cancer | Bai 2017 | 13/6/7 | No serious risk | No serious inconsistency | No serious indirectness | No serious imprecision | Undetected | Would not reduce effect | No | No | low |
| physical activity | high vs low levels | risk of bladder cancer | Keimling 2014 | 15/11/4 | No serious risk | Serious inconsistency | No serious indirectness | Serious imprecision | Undetected | Would not reduce effect | No | No | very low |
| opium consumption | With vs. without | risk of bladder cancer | Filho 2023 | 15/1/14 | No serious risk | No serious inconsistency | No serious indirectness | No serious imprecision | Undetected | Would not reduce effect | Yes | No | moderate |
| Aspirin | Mean duration of use =>5 years | risk of bladder cancer | Fan 2021 | 7/3/4 | No serious risk | Serious inconsistency | No serious indirectness | No serious imprecision | Undetected | Would not reduce effect | No | No | very low |
| Aspirin | Mean duration of use < 5 years | risk of bladder cancer | Fan 2021 | 8/4/4 | No serious risk | Serious inconsistency | No serious indirectness | Serious imprecision | Undetected | Would not reduce effect | No | No | very low |
| Pioglitazone | With vs. without | risk of bladder cancer | Tang 2018 | 20/12/8 | No serious risk | No serious inconsistency | No serious indirectness | Serious imprecision | Undetected | Would reduce effect | No | No | low |
| ACEI | With vs. without | risk of bladder cancer | Xie 2020 | 4/2/2 | No serious risk | No serious inconsistency | No serious indirectness | Serious imprecision | Undetected | Would not reduce effect | No | No | very low |
| ARB | With vs. without | risk of bladder cancer | Xie 2020 | 3/2/1 | No serious risk | No serious inconsistency | No serious indirectness | No serious imprecision | Undetected | Would not reduce effect | No | No | low |
| Red Meat Intake | high versus low | risk of bladder cancer | Yu 2023 | 20/8/12 | No serious risk | Serious inconsistency | No serious indirectness | Serious imprecision | Strongly suspected | Would not reduce effect | No | No | very low |
| Processed meat | high versus low | risk of bladder cancer | Yu 2023 | 19/6/13 | No serious risk | No serious inconsistency | No serious indirectness | No serious imprecision | Undetected | Would not reduce effect | No | No | low |
| Fish | high versus low | risk of bladder cancer | Yu 2023 | 15/4/11 | No serious risk | Serious inconsistency | No serious indirectness | Serious imprecision | Undetected | Would not reduce effect | No | No | very low |
| processed meat consumption | 50 g per day incremen | risk of bladder cancer | Crippa 2018 | 10/4/6 | Serious risk | No serious inconsistency | No serious indirectness | Serious imprecision | Undetected | Would not reduce effect | No | Yes | very low |
| red meat consumption | 100 g per day increment | risk of bladder cancer | Crippa 2018 | 12/5/7 | Serious risk | Serious inconsistency | No serious indirectness | Serious imprecision | Undetected | Would not reduce effect | No | Yes | very low |
| total fruit and vegetables | high versus low | risk of bladder cancer | Yao 2014 | 10/8/2 | Serious risk | Serious inconsistency | No serious indirectness | Serious imprecision | Undetected | Would not reduce effect | No | No | very low |
| total vegetables | high versus low | risk of bladder cancer | Yao 2014 | 20/8/12 | Serious risk | Serious inconsistency | No serious indirectness | No serious imprecision | Undetected | Would not reduce effect | No | No | very low |
| total fruit | high versus low | risk of bladder cancer | Yao 2014 | 26/10/16 | Serious risk | Serious inconsistency | No serious indirectness | No serious imprecision | Strongly suspected | Would reduce effect | No | Yes | very low |
| vitamin E | high versus low | risk of bladder cancer | Lin 2019 | 8/8/0 | Serious risk | No serious inconsistency | No serious indirectness | Serious imprecision | Undetected | Would not reduce effect | No | No | very low |
| Dietary fat intake | highest vs lowest | risk of bladder cancer | Wang 2019 | 10/2/8 | Serious risk | Serious inconsistency | No serious indirectness | Serious imprecision | Undetected | Would not reduce effect | No | No | very low |
| Tetrachloroethylene Exposure | Exposure vs.non-Exposure | risk of bladder cancer | Vlaanderen 2014 | 8/3/5 | Serious risk | No serious inconsistency | No serious indirectness | Serious imprecision | Strongly suspected | Would not reduce effect | No | No | very low |
| painters | painters vs. non-painters | risk of bladder cancer | Guha 2010 | 41/11/30 | Serious risk | No serious inconsistency | No serious indirectness | No serious imprecision | Undetected | Would reduce effect | No | No | low |
| rubber-manufacturing industry | rubber-manufacturing industry vs. non-rubber-manufacturing industry | risk of bladder cancer | Boniol 2017 | 54/35/19 | Serious risk | Serious inconsistency | No serious indirectness | No serious imprecision | Undetected | Would not reduce effect | No | No | very low |
| WD | highest category | risk of bladder cancer | Dianatinasab 2022 | 4/2/2 | No serious risk | No serious inconsistency | No serious indirectness | No serious imprecision | NA | Would not reduce effect | No | No | low |
| MD | highest category | risk of bladder cancer | Dianatinasab 2022 | 6/4/2 | No serious risk | Serious inconsistency | No serious indirectness | Serious imprecision | NA | Would not reduce effect | No | No | very low |
| early menopause | menopause vs. non-menopause | risk of bladder cancer | Xu 2020 | 7/7/0 | Serious risk | No serious inconsistency | No serious indirectness | Serious imprecision | NA | Would not reduce effect | No | No | very low |
| Tooth loss | highest vs lowest | risk of bladder cancer | Shi 2018 | 2/2/0 | No serious risk | No serious inconsistency | No serious indirectness | No serious imprecision | Undetected | Would not reduce effect | No | No | low |
| pesticide exposure | pesticide exposure vs. non-pesticide exposure | risk of bladder cancer | Liang 2016 | 10/3/7 | No serious risk | Serious inconsistency | No serious indirectness | Serious imprecision | Undetected | Would not reduce effect | No | No | very low |
| Metformin | ever users vs. never users | risk of bladder cancer | Zhang 2021 | 3/3/0 | Serious risk | Serious inconsistency | No serious indirectness | Serious imprecision | Undetected | Would not reduce effect | No | No | very low |
| Insulin Glargine | insulin glargine versus non-glargine insulin. | risk of bladder cancer | Tang 2012 | 4/4/0 | No serious risk | NA | No serious indirectness | Serious imprecision | Strongly suspected | Would not reduce effect | No | No | very low |
| AVV | With vs. without | risk of bladder cancer | Shang 2015 | 5/5/0 | No serious risk | No serious inconsistency | No serious indirectness | No serious imprecision | Undetected | Would not reduce effect | Yes | No | moderate |
| total fluid intake | highest intake vs. lowest intake) | risk of bladder cancer | Hong 2018 | 20/18/2 | No serious risk | Serious inconsistency | No serious indirectness | Serious imprecision | Undetected | Would not reduce effect | No | No | very low |
| chlorinated drinking water | long term consumption | risk of bladder cancer | Villanueva 2003 | 5/0/5 | Serious risk | NA | No serious indirectness | No serious imprecision | Undetected | Would not reduce effect | No | No | very low |
| 5-ARI | With vs. without | risk of bladder cancer | Xiang 2021 | 4/3/1 | No serious risk | No serious inconsistency | No serious indirectness | No serious imprecision | NA | Would not reduce effect | No | No | low |
| TZD | use vs. no use | risk of bladder cancer | BOSETTI 2013 | 7/7/0 | No serious risk | No serious inconsistency | No serious indirectness | Serious imprecision | Undetected | Would not reduce effect | No | No | very low |
| thiazolidinediones: Cumulative dose of pioglitazone | Cumulative dose of pioglitazone | risk of bladder cancer | BOSETTI 2013 | 5/5/0 | No serious risk | Serious inconsistency | No serious indirectness | Serious imprecision | Undetected | Would not reduce effect | No | No | very low |
| Cruciferous Vegetable Intake | At least 412.5 g per week | risk of bladder cancer | Zheng 2024 | 6/0/6 | No serious risk | No serious inconsistency | No serious indirectness | Serious imprecision | Undetected | Would not reduce effect | No | Yes | low |
| Cruciferous Vegetable Intake | Unspecified | risk of bladder cancer | Zheng 2024 | 6/0/6 | No serious risk | Serious inconsistency | No serious indirectness | Serious imprecision | Undetected | Would not reduce effect | No | No | very low |
| Broccoli Consumption | High intake vs. low intake | risk of bladder cancer | Baladia 2024 | 3/0/3 | No serious risk | No serious inconsistency | No serious indirectness | No serious imprecision | Undetected | Would not reduce effect | No | No | low |
| Broccoli Consumption | High intake vs. low intake | risk of bladder cancer | Baladia 2024 | 1/1/0 | No serious risk | NA | No serious indirectness | Serious imprecision | Undetected | Would not reduce effect | No | No | very low |
| AO | Military Veterans vs. general population | risk of bladder cancer | Kronstedt 2024 | 4/4/0 | No serious risk | Serious inconsistency | No serious indirectness | Serious imprecision | NA | Would not reduce effect | No | No | very low |
| DU | Military Veterans vs. general population | risk of bladder cancer | Kronstedt 2024 | 4/4/0 | No serious risk | No serious inconsistency | No serious indirectness | Serious imprecision | NA | Would not reduce effect | Yes | No | low |
| hairdressers | Ever registered for employment | risk of bladder cancer | Harling 2010 | 18/8/10 | Serious risk | NA | No serious indirectness | No serious imprecision | Undetected | Would reduce effect | No | No | very low |
| hairdressers | Job held ≥ 10 years | risk of bladder cancer | Harling 2010 | 6/0/6 | Serious risk | NA | No serious indirectness | Serious imprecision | Undetected | Would not reduce effect | No | No | very low |
| male career firefighters | general, uniformed service, or working population referent | risk of bladder cancer | DeBono 2023 | 10/10/0 | Serious risk | No serious inconsistency | No serious indirectness | No serious imprecision | Undetected | Would not reduce effect | No | No | very low |
| occupational exposure to inorganic lead | exposure to inorganic lead vs. non-exposure to inorganic lead | risk of bladder cancer | Fu 1995 | 5/4/1 | Serious risk | NA | No serious indirectness | No serious imprecision | NA | Would not reduce effect | No | No | very low |
| breast cancer in women | breast cancer diagnosed at any age | risk of bladder cancer | Allen 2023 | 8/8/0 | No serious risk | NA | No serious indirectness | Serious imprecision | Undetected | Would not reduce effect | No | No | very low |
| breast cancer in women | breast cancer diagnosed at underage 50 | risk of bladder cancer | Allen 2023 | 4/4/0 | No serious risk | NA | No serious indirectness | No serious imprecision | Undetected | Would not reduce effect | No | No | very low |
| systemic sclerosis | With vs. without | risk of bladder cancer | Onishi 2013 | 2/2/0 | No serious risk | NA | No serious indirectness | Serious imprecision | Undetected | Would not reduce effect | Yes | No | very low |
| Depression and anxiety | With vs. without | risk of bladder cancer | Wang 2020 | 4/4/0 | No serious risk | Serious inconsistency | No serious indirectness | Serious imprecision | Undetected | Would not reduce effect | No | No | very low |
| Psoriasis and Psoriatic Arthritis | With vs. without | risk of bladder cancer | Vaengebjerg 2020 | 9/9/0 | Serious risk | No serious inconsistency | No serious indirectness | Serious imprecision | Undetected | Would not reduce effect | No | No | very low |
| Diesel Exhaust Exposure | Heavy equipment operators vs. non-Heavy equipment operators | risk of bladder cancer | Boffetta 2001 | 2/1/1 | Serious risk | NA | No serious indirectness | Serious imprecision | Strongly suspected | Would not reduce effect | No | No | very low |
| Diesel Exhaust Exposure | Truck drivers vs. non-Truck drivers | risk of bladder cancer | Boffetta 2001 | 11/0/11 | Serious risk | NA | No serious indirectness | Serious imprecision | Strongly suspected | Would not reduce effect | No | No | very low |
| Diesel Exhaust Exposure | Bus drivers vs. non-Bus drivers | risk of bladder cancer | Boffetta 2001 | 7/1/6 | Serious risk | NA | No serious indirectness | No serious imprecision | Strongly suspected | Would not reduce effect | No | No | very low |
| Dietary GI | highest versus lowest | risk of bladder cancer | Long 2022 | 3/3/0 | No serious risk | No serious inconsistency | No serious indirectness | No serious imprecision | Undetected | Would not reduce effect | No | No | low |
| Alcohol source：Liquor or spirits | 12 g increment | risk of bladder cancer | Lao 2021 | 5/5/0 | No serious risk | NA | No serious indirectness | Serious imprecision | NA | Would not reduce effect | No | Yes | very low |
| coal mine workers | coal mine workers vs. non-coal mine workers | risk of bladder cancer | Alif 2022 | 2/0/2 | No serious risk | NA | No serious indirectness | Serious imprecision | Undetected | Would not reduce effect | Yes | No | very low |
| Milk | high compared with low dairy product intake | risk of bladder cancer | Bermej 2019 | 18/12/6 | No serious risk | Serious inconsistency | No serious indirectness | Serious imprecision | Strongly suspected | Would not reduce effect | No | No | very low |
| Whole milk | high compared with low dairy product intake | risk of bladder cancer | Bermej 2019 | 4/0/4 | No serious risk | Serious inconsistency | No serious indirectness | Serious imprecision | Strongly suspected | Would not reduce effect | No | No | very low |
| Fermented dairy products | high compared with low dairy product intake | risk of bladder cancer | Bermej 2019 | 5/3/2 | No serious risk | Serious inconsistency | No serious indirectness | Serious imprecision | Strongly suspected | Would not reduce effect | No | No | very low |
| Secondhand smoking | With vs. without | risk of bladder cancer | Yan 2018 | 14/3/11 | No serious risk | No serious inconsistency | No serious indirectness | Serious imprecision | Undetected | Would not reduce effect | No | No | very low |
| smoking intensity | cigarettes/day (highest vs. lowestcategoryy) | risk of bladder cancer | Zhao 2022 | 31/7/24 | No serious risk | Serious inconsistency | No serious indirectness | No serious imprecision | Undetected | Would not reduce effect | No | No | very low |
| **Non-significant** |  |  |  |  |  |  |  |  |  |  |  |  |  |
| Total meat | high versus low | risk of bladder cancer | Yu 2023 | 11/2/9 | No serious risk | Serious inconsistency | No serious indirectness | Serious imprecision | Undetected | Would not reduce effect | No | No | very low |
| vitamin E | high versus low | risk of bladder cancer | Lin 2019 | 2 RCT | Serious risk | No serious inconsistency | No serious indirectness | Serious imprecision | Undetected | Would not reduce effect | No | No | low |
| Androgen suppression therapy：ADT | lack of AST | risk of bladder cancer | Xiang 2021 | 4/4/0 | No serious risk | Serious inconsistency | No serious indirectness | Serious imprecision | NA | Would not reduce effect | No | No | very low |
| Androgen suppression therapy:total | lack of AST | risk of bladder cancer | Xiang 2021 | 8/7/1 | No serious risk | Serious inconsistency | No serious indirectness | Serious imprecision | NA | Would not reduce effect | No | No | very low |
| Rosiglitazone | use vs. no use | risk of bladder cancer | BOSETTI 2013 | 3/3/0 | No serious risk | No serious inconsistency | No serious indirectness | Serious imprecision | Undetected | Would not reduce effect | No | No | very low |
| Cruciferous Vegetable Intake | Unspecified | risk of bladder cancer | Zheng 2024 | 10/10/0 | No serious risk | No serious inconsistency | No serious indirectness | Serious imprecision | Strongly suspected | Would not reduce effect | No | No | very low |
| male career firefighters | duration of employment:<10 yrs | risk of bladder cancer | DeBono 2023 | 4/4/0 | Serious risk | No serious inconsistency | No serious indirectness | Serious imprecision | Undetected | Would not reduce effect | No | No | very low |
| male career firefighters | duration of employment:10-20 yrs | risk of bladder cancer | DeBono 2023 | 4/4/0 | Serious risk | No serious inconsistency | No serious indirectness | Serious imprecision | Undetected | Would not reduce effect | No | No | very low |
| male career firefighters | duration of employment:>20 yrs | risk of bladder cancer | DeBono 2023 | 4/4/0 | Serious risk | No serious inconsistency | No serious indirectness | Serious imprecision | Undetected | Would not reduce effect | No | No | very low |
| breast cancer in women | breast cancer diagnosed at age 50 or over | risk of bladder cancer | Allen 2023 | 4/4/0 | No serious risk | NA | No serious indirectness | Serious imprecision | Undetected | Would not reduce effect | No | No | very low |
| Duration of pioglitazone | Duration of pioglitazone | risk of bladder cancer | BOSETTI 2013 | 11/9/2 | No serious risk | Serious inconsistency | No serious indirectness | Serious imprecision | Undetected | Would not reduce effect | No | No | very low |
| hairdressers | Job held ≥ 5 years | risk of bladder cancer | Harling 2010 | 3/1/2 | Serious risk | NA | No serious indirectness | Serious imprecision | Undetected | Would not reduce effect | No | No | very low |
| ankylosing spondylitis | With vs. without | risk of bladder cancer | Yu 2024 | 5/5/0 | No serious risk | Serious inconsistency | No serious indirectness | Serious imprecision | Undetected | Would not reduce effect | No | No | very low |
| inflammatory bowel | With vs. without | risk of bladder cancer | Zhang 2021 | 11/9/2 | No serious risk | No serious inconsistency | No serious indirectness | Serious imprecision | Undetected | Would not reduce effect | No | No | very low |
| disinfection by-products | Chloroform | risk of bladder cancer | Shi 2024 | 3/1/2 | No serious risk | Serious inconsistency | No serious indirectness | Serious imprecision | Undetected | Would not reduce effect | No | No | very low |
| ABO blood group | AB vs O | risk of bladder cancer | Cui 2023 | 5/0/5 | No serious risk | No serious inconsistency | No serious indirectness | Serious imprecision | Undetected | Would not reduce effect | No | No | very low |
| ABO blood group | B vs O | risk of bladder cancer | Cui 2023 | 5/0/5 | No serious risk | No serious inconsistency | No serious indirectness | Serious imprecision | Undetected | Would not reduce effect | No | No | very low |
| Age of menarche | Menarcheal age | risk of bladder cancer | Li 2022 | 12/10/2 | No serious risk | No serious inconsistency | No serious indirectness | Serious imprecision | Undetected | Would not reduce effect | No | No | very low |
| overweight (BMI=>25 kg/m2 | With vs. without | risk of bladder cancer | Ahmadinezhad 2022 | 23/23/0 | No serious risk | Serious inconsistency | No serious indirectness | Serious imprecision | Undetected | Would not reduce effect | No | No | very low |
| obesity (BMI =>30kg/m2 | With vs. without | risk of bladder cancer | Ahmadinezhad 2022 | 23/23/0 | No serious risk | Serious inconsistency | No serious indirectness | Serious imprecision | Undetected | Would not reduce effect | No | No | very low |
| Pioglitazone | With vs. without | risk of bladder cancer | Tang 2018 | 2 RCT | No serious risk | No serious inconsistency | No serious indirectness | Serious imprecision | Undetected | Would not reduce effect | No | No | moderate |
| acetaminophen | regular/any use | risk of bladder cancer | Zhang 2013 | 10/2/8 | Serious risk | No serious inconsistency | No serious indirectness | Serious imprecision | Undetected | Would not reduce effect | No | No | very low |
| aspirin | regular/any use | risk of bladder cancer | Zhang 2013 | 11/6/5 | Serious risk | No serious inconsistency | No serious indirectness | Serious imprecision | Undetected | Would not reduce effect | No | No | very low |
| non-aspirin NSAIDs | regular/any use | risk of bladder cancer | Zhang 2013 | 6/3/3 | Serious risk | Serious inconsistency | No serious indirectness | Serious imprecision | Undetected | Would not reduce effect | No | No | very low |
| CCB | With vs. without | risk of bladder cancer | Xie 2020 | 6/2/4 | No serious risk | Serious inconsistency | No serious indirectness | Serious imprecision | Undetected | Would not reduce effect | No | No | very low |
| diuretics | With vs. without | risk of bladder cancer | Xie 2020 | 2/0/2 | No serious risk | Serious inconsistency | No serious indirectness | Serious imprecision | Undetected | Would not reduce effect | No | No | very low |
| Egg | egg consumption | risk of bladder cancer | Li 2013 | 13/4/9 | Serious risk | Serious inconsistency | No serious indirectness | Serious imprecision | Undetected | Would not reduce effect | No | No | very low |
| coffee consumption | Each 1 cup/d increment | risk of bladder cancer | Dai 2019 | 15/15/0 | Serious risk | Serious inconsistency | No serious indirectness | Serious imprecision | Undetected | Would not reduce effect | No | Yes | very low |
| total carotenoid intake | highest compared with lowest categories | risk of bladder cancer | Wu 2020 | 11/4/7 | No serious risk | Serious inconsistency | No serious indirectness | Serious imprecision | Undetected | Would not reduce effect | No | No | very low |
| circulating carotenoid concentrations | highest with the lowest | risk of bladder cancer | Wu 2020 | 3/0/3 | No serious risk | Serious inconsistency | No serious indirectness | Serious imprecision | Undetected | Would not reduce effect | No | No | very low |
| White meat | high versus low | risk of bladder cancer | Yu 2023 | 15/6/9 | No serious risk | Serious inconsistency | No serious indirectness | Serious imprecision | Undetected | Would not reduce effect | No | No | very low |
| tea consumption | highest vs. lowest | risk of bladder cancer | Weng 2017 | 32/7/25 | No serious risk | Serious inconsistency | No serious indirectness | Serious imprecision | Undetected | Would not reduce effect | No | No | very low |
| black tea consumption | high versus low | risk of bladder cancer | Weng 2017 | 10/3/7 | No serious risk | No serious inconsistency | No serious indirectness | Serious imprecision | Undetected | Would not reduce effect | No | No | very low |
| green tea consumption | high versus low | risk of bladder cancer | Weng 2017 | 7/3/4 | No serious risk | Serious inconsistency | No serious indirectness | Serious imprecision | Undetected | Would not reduce effect | No | No | very low |
| Dietary Inflammatory Potential | highest versus the lowest | risk of bladder cancer | Dai 2024 | 6/3/3 | No serious risk | Serious inconsistency | No serious indirectness | Serious imprecision | Strongly suspected | Would not reduce effect | No | No | very low |
| total fluid intake | highest vs lowest | risk of bladder cancer | Liu 2017 | 26/5/21 | No serious risk | No serious inconsistency | No serious indirectness | Serious imprecision | Undetected | Would not reduce effect | No | No | very low |
| occupational exposure to welding fumes | exposure vs. non-exposure | risk of bladder cancer | Collatuzzo 2024 | 5/5/0 | No serious risk | Serious inconsistency | No serious indirectness | Serious imprecision | Undetected | Would not reduce effect | No | No | very low |
| benzene exposure | exposure vs. non-exposure | risk of bladder cancer | Seyyedsalehi 2024 | 35/30/5 | No serious risk | No serious inconsistency | No serious indirectness | Serious imprecision | Undetected | Would not reduce effect | No | No | very low |
| occupational asbestos exposure | exposure vs. non-exposure | risk of bladder cancer | Franco 2023 | 28/28/0 | No serious risk | Serious inconsistency | No serious indirectness | Serious imprecision | Undetected | Would not reduce effect | No | No | very low |
| hepatitis C virus infection | With vs. without | risk of bladder cancer | Ma 2021 | 5/3/2 | Serious risk | No serious inconsistency | No serious indirectness | Serious imprecision | Undetected | Would not reduce effect | No | No | very low |
| periodontal disease | With vs. without | risk of bladder cancer | Li 2022 | 3/3/0 | No serious risk | No serious inconsistency | No serious indirectness | Serious imprecision | Undetected | Would not reduce effect | No | No | very low |
| DII | highest vs lowest | risk of bladder cancer | Dianatinasab 2022 | 4/2/2 | No serious risk | Serious inconsistency | No serious indirectness | Serious imprecision | NA | Would not reduce effect | No | No | very low |
| Bitumen workers | Bitumen workers vs. non-Bitumen workers | risk of bladder cancer | Mundt 2018 | 21/15/6 | No serious risk | No serious inconsistency | No serious indirectness | Serious imprecision | Undetected | Would not reduce effect | No | No | very low |
| roofers | roofers vs. non-roofers | risk of bladder cancer | Mundt 2018 | 7/5/2 | No serious risk | No serious inconsistency | No serious indirectness | Serious imprecision | Undetected | Would not reduce effect | No | No | very low |
| pavers | pavers vs.non-pavers | risk of bladder cancer | Mundt 2018 | 11/10/1 | No serious risk | No serious inconsistency | No serious indirectness | Serious imprecision | Undetected | Would not reduce effect | No | No | very low |
| Soft drinks | highest vs. lowest | risk of bladder cancer | Boyle 2014 | 5/5/0 | Serious risk | No serious inconsistency | No serious indirectness | Serious imprecision | Undetected | Would not reduce effect | No | No | very low |
| estrogen-progestogen therapy for hormone replacement therapy | With vs. without | risk of bladder cancer | Xu 2020 | 5/5/0 | Serious risk | Serious inconsistency | No serious indirectness | Serious imprecision | NA | Would not reduce effect | No | No | very low |
| Personal hair dye use | any type of hair dyes compared with no use | risk of bladder cancer | Turati 2014 | 17/2/15 | Serious risk | No serious inconsistency | No serious indirectness | Serious imprecision | Undetected | Would not reduce effect | No | No | very low |
| Personal hair dye use | personal use of permanent hair dyes compared with no use | risk of bladder cancer | Turati 2014 | 7/1/6 | Serious risk | No serious inconsistency | No serious indirectness | Serious imprecision | Undetected | Would not reduce effect | No | No | very low |
| nitrate | daily nitrate intake | risk of bladder cancer | Arafa 2022 | 3/1/2 | No serious risk | Serious inconsistency | No serious indirectness | Serious imprecision | Undetected | Would not reduce effect | No | No | very low |
| Sodium-glucose co-transporter-2 inhibitors | With vs. without | risk of bladder cancer | Spiazzi 2024 | 6RCT | No serious risk | No serious inconsistency | No serious indirectness | Serious imprecision | Undetected | Would not reduce effect | No | No | moderate |
| statins | statins vs. placebos | risk of bladder cancer | Symvoulidis 2023 | 4RCT | No serious risk | No serious inconsistency | No serious indirectness | Serious imprecision | Undetected | Would not reduce effect | No | No | moderate |
| statins | statins vs. controls | risk of bladder cancer | Symvoulidis 2023 | 6RCT | No serious risk | Serious inconsistency | No serious indirectness | Serious imprecision | Undetected | Would not reduce effect | No | No | low |
| alcohol consumption | Light versus none | risk of bladder cancer | Lao 2021 | 2/2/0 | No serious risk | No serious inconsistency | No serious indirectness | Serious imprecision | NA | Would not reduce effect | No | No | very low |
| alcohol consumption | moderate versus none | risk of bladder cancer | Lao 2021 | 2/2/0 | No serious risk | Serious inconsistency | No serious indirectness | Serious imprecision | NA | Would not reduce effect | No | No | very low |
| alcohol consumption | heavy versus none | risk of bladder cancer | Lao 2021 | 2/2/0 | No serious risk | No serious inconsistency | No serious indirectness | Serious imprecision | NA | Would not reduce effect | No | No | very low |
| Alcohol source：Beer | 12 g (1 drink) increment of alcohol | risk of bladder cancer | Lao 2021 | 5/5/0 | No serious risk | NA | No serious indirectness | Serious imprecision | NA | Would not reduce effect | No | Yes | very low |
| Alcohol source：Wine | 12 g (1 drink) increment of alcohol | risk of bladder cancer | Lao 2021 | 4/4/0 | No serious risk | NA | No serious indirectness | Serious imprecision | NA | Would not reduce effect | No | Yes | very low |
| Vitamin A | High dose vs. low dose | risk of bladder cancer | Park 2017 | 5RCT | No serious risk | Serious inconsistency | No serious indirectness | Serious imprecision | Undetected | Would not reduce effect | No | No | low |
| Vitamin B6 | High dose vs. low dose | risk of bladder cancer | Park 2017 | 3RCT | No serious risk | Serious inconsistency | No serious indirectness | Serious imprecision | Undetected | Would not reduce effect | No | No | low |
| Vitamin C | High dose vs. low dose | risk of bladder cancer | Park 2017 | 2RCT | No serious risk | Serious inconsistency | No serious indirectness | Serious imprecision | Undetected | Would not reduce effect | No | No | low |
| Vitamin D | High dose vs. low dose | risk of bladder cancer | Park 2017 | 1RCT | No serious risk | NA | No serious indirectness | Serious imprecision | Undetected | Would not reduce effect | No | No | low |
| Beta-carotene | High dose vs. low dose | risk of bladder cancer | Park 2017 | 6RCT | No serious risk | No serious inconsistency | No serious indirectness | Serious imprecision | Undetected | Would not reduce effect | No | No | moderate |
| nitrate | High dose vs. low dose | risk of bladder cancer | Seyyedsalehi 2021 | 10/6/4 | No serious risk | Serious inconsistency | No serious indirectness | Serious imprecision | Undetected | Would not reduce effect | No | No | very low |
| nitrate | Moderate dose vs. low dose | risk of bladder cancer | Seyyedsalehi 2021 | 12/4/8 | No serious risk | Serious inconsistency | No serious indirectness | Serious imprecision | Undetected | Would not reduce effect | No | No | very low |
| nitrite | High dose vs. low dose | risk of bladder cancer | Seyyedsalehi 2021 | 6/5/1 | No serious risk | Serious inconsistency | No serious indirectness | Serious imprecision | Undetected | Would not reduce effect | No | No | very low |
| nitrite | Moderate dose vs. low dose | risk of bladder cancer | Seyyedsalehi 2021 | 7/5/2 | No serious risk | Serious inconsistency | No serious indirectness | Serious imprecision | Undetected | Would not reduce effect | No | No | very low |
| sugar-sweetened beverages | highest vs. lowest | risk of bladder cancer | Li 2021 | 6/1/5 | No serious risk | No serious inconsistency | No serious indirectness | Serious imprecision | Strongly suspected | Would not reduce effect | No | No | very low |
| coal mine workers | coal mine workers vs .non-coal mine workers | risk of bladder cancer | Alif 2022 | 4/4/0 | No serious risk | NA | No serious indirectness | Serious imprecision | Undetected | Would not reduce effect | No | No | very low |
| acrylonitrile workers | acrylonitrile workers vs. non-acrylonitrile workers | risk of bladder cancer | Collins 1998 | 3/3/0 | Serious risk | NA | No serious indirectness | Serious imprecision | Undetected | Would not reduce effect | No | No | very low |
| Potato Consumption | high and low intake | risk of bladder cancer | Mofrad 2021 | 5/0/5 | No serious risk | NA | No serious indirectness | Serious imprecision | Undetected | Would not reduce effect | No | No | very low |
| total dairy products | high compared with low dairy product intake | risk of bladder cancer | Bermej 2019 | 6/2/4 | No serious risk | Serious inconsistency | No serious indirectness | Serious imprecision | Strongly suspected | Would not reduce effect | No | No | very low |
| Cheese | high compared with low dairy product intake | risk of bladder cancer | Bermej 2019 | 7/3/4 | No serious risk | Serious inconsistency | No serious indirectness | Serious imprecision | Strongly suspected | Would not reduce effect | No | No | very low |
| Butter | high compared with low dairy product intake | risk of bladder cancer | Bermej 2019 | 5/2/3 | No serious risk | No serious inconsistency | No serious indirectness | Serious imprecision | Strongly suspected | Would not reduce effect | No | No | very low |
| Spironolactone | With vs. without | risk of bladder cancer | Bommareddy 2022 | 3/1/2 | No serious risk | Serious inconsistency | No serious indirectness | Serious imprecision | NA | Would not reduce effect | No | No | very low |
| rhinitis | With vs. without | risk of bladder cancer | Feng 2021 | 4/4/0 | No serious risk | Serious inconsistency | No serious indirectness | Serious imprecision | Undetected | Would not reduce effect | No | No | very low |
| contaminated drinking water | Military Veterans vs. general population | risk of bladder cancer | Kronstedt 2024 | 2/2/0 | No serious risk | No serious inconsistency | No serious indirectness | Serious imprecision | NA | Would not reduce effect | No | No | very low |
| estrogen-progestogen therapy for hormone replacement therapy | estrogen-progestogen therapy vs. non-estrogen-progestogen therapy | risk of bladder cancer | Xu 2020 | 7/7/0 | Serious risk | No serious inconsistency | No serious indirectness | Serious imprecision | NA | Would not reduce effect | No | No | very low |
| Cruciferous Vegetable Intake | Intake >3.5 servings/wk | risk of bladder cancer | Zheng 2024 | 10/10/0 | No serious risk | No serious inconsistency | No serious indirectness | Serious imprecision | Strongly suspected | Would not reduce effect | No | No | very low |

WD, Western diet; MD, Mediterranean diet; GI, glycemic index; DII, dietary-inflammatory-index; BMI, Body Mass Index; LS, Lynch syndrome; DBP, Diastolic blood pressure; PM2.5, Particulate Matter with a diameter of less than or equal to 2.5 micrometers; NO₂, Nitrogen Dioxide; MIBC, Muscle-invasive bladder cancer; BPH, Benign prostatic hyperplasia; AAV, ANCA-associated vasculitis; NSAIDs, Non-Steroidal Anti-Inflammatory Drugs; CCB, Calcium channel blockers; ADT, androgen deprivation therapy; AST, androgen suppression therapy; ACEI, angiotensin-converting enzyme inhibitors; ARB, angiotensin II receptor blockers; 5-ARI, 5-alpha reductase inhibitor; TZD, thiazolidinediones; TTHM, total trihalomethanes; AO, Agent Orange; DU, Depleted uranium; T, Total study; C, Cohort study; P, Case-control study; NA, Not Available


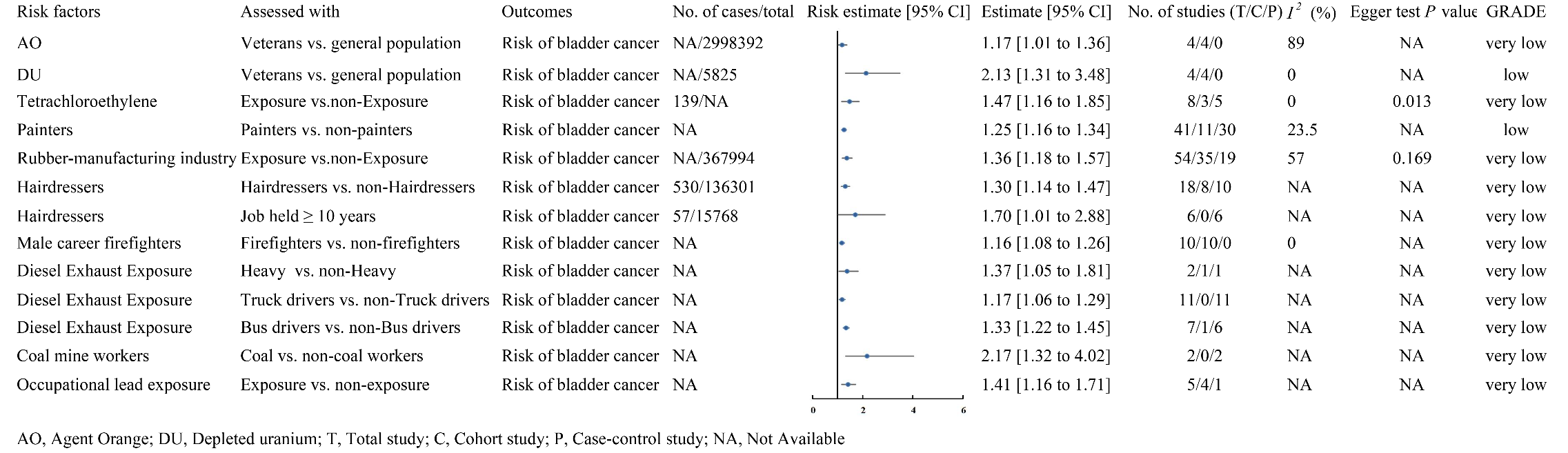


Figure S1 The Impact of Occupational Factors on Bladder Cancer Risk.


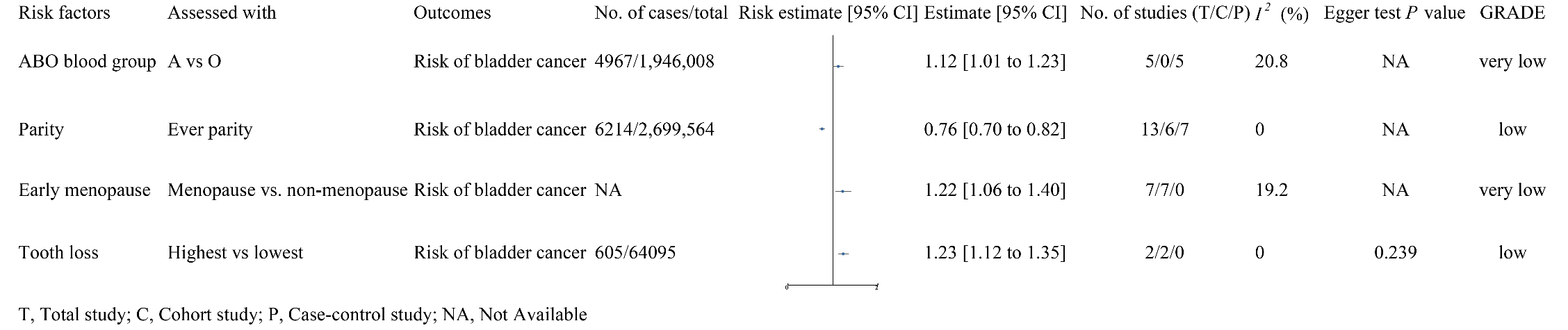


Figure S2 The Impact of Physiological Factors on Bladder Cancer Risk.


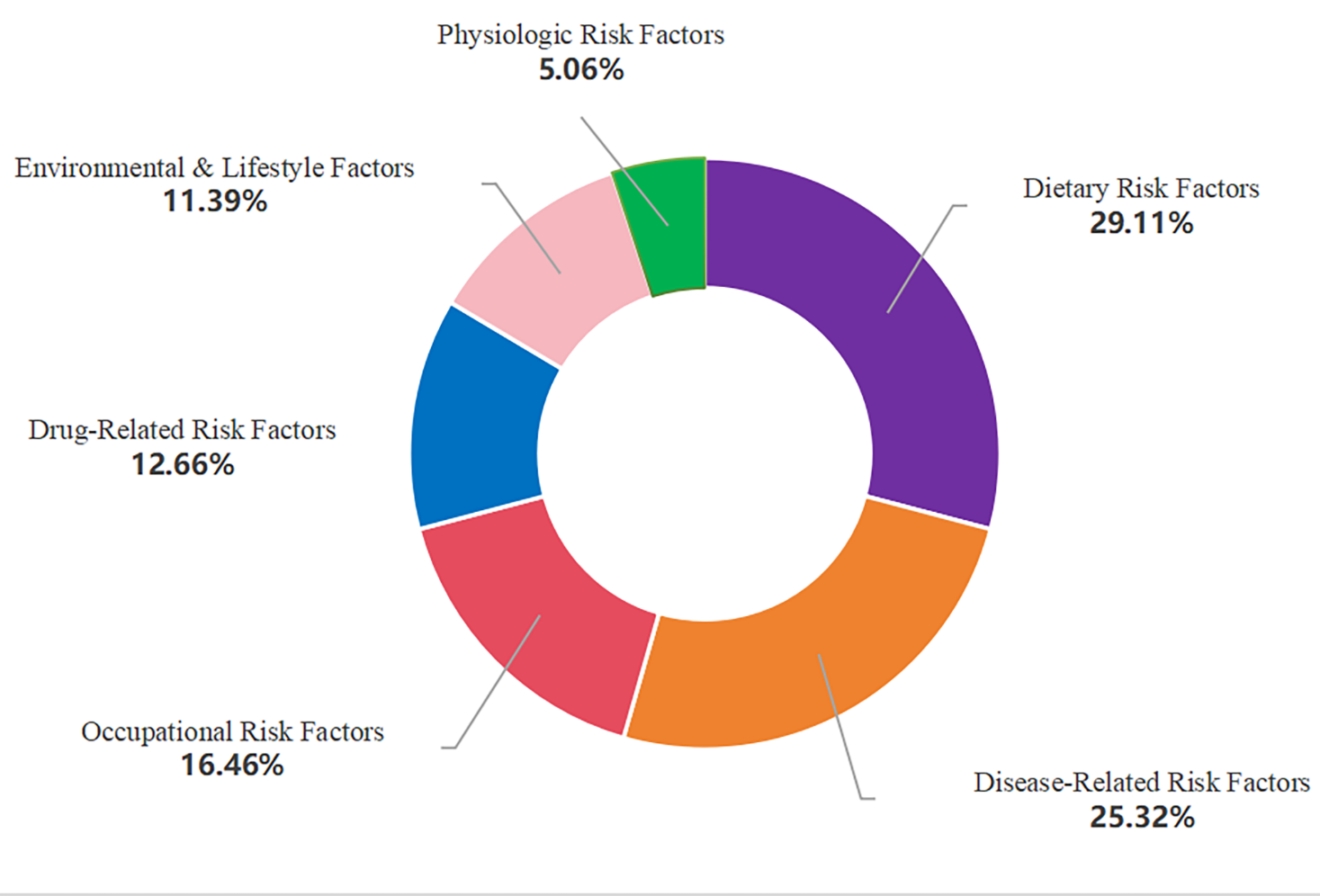


Figure S3 Result Figures Related to Bladder Cancer Risk Factors


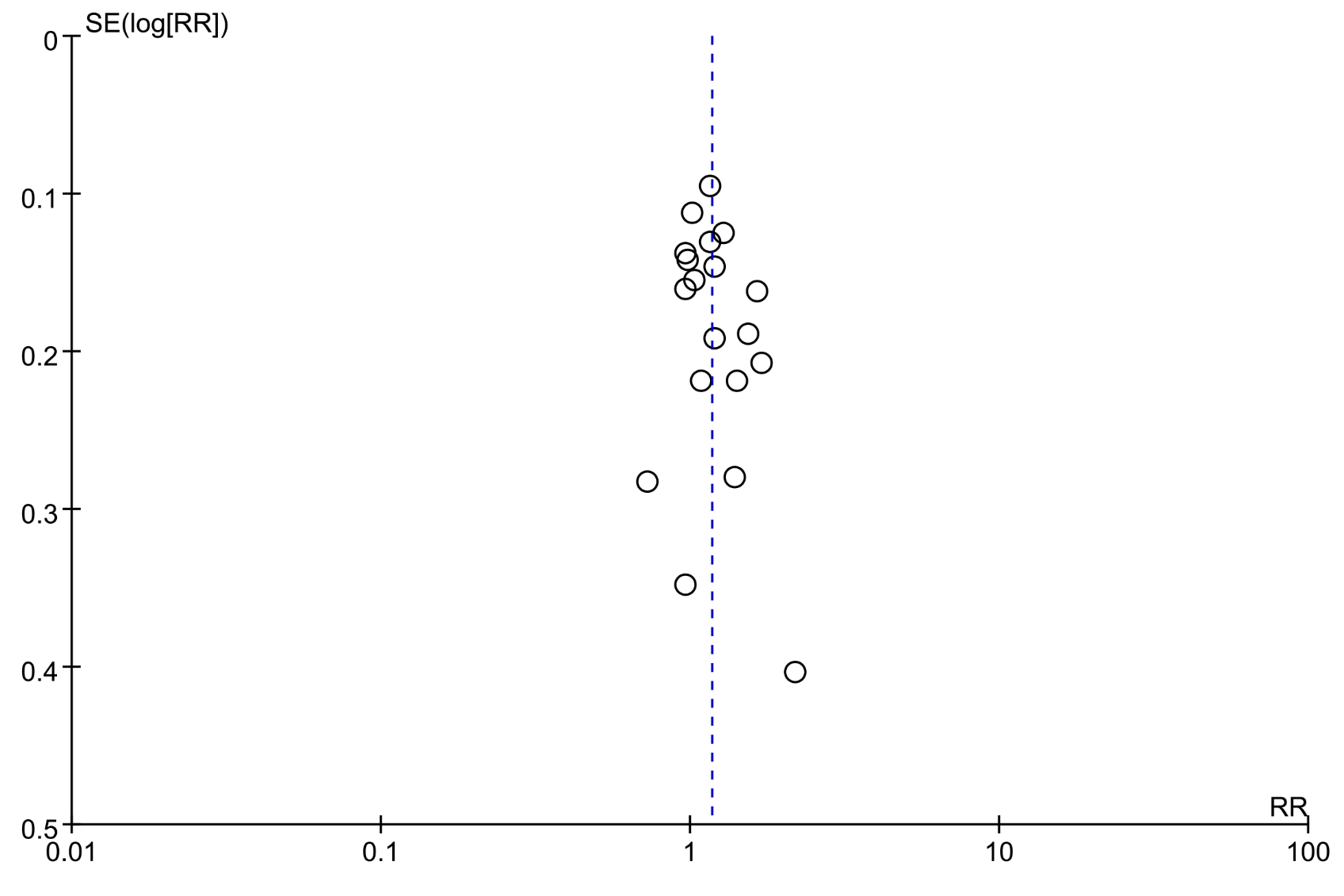


Figure S4 Funnel Plot of Processed Meat and Bladder Cancer Risk


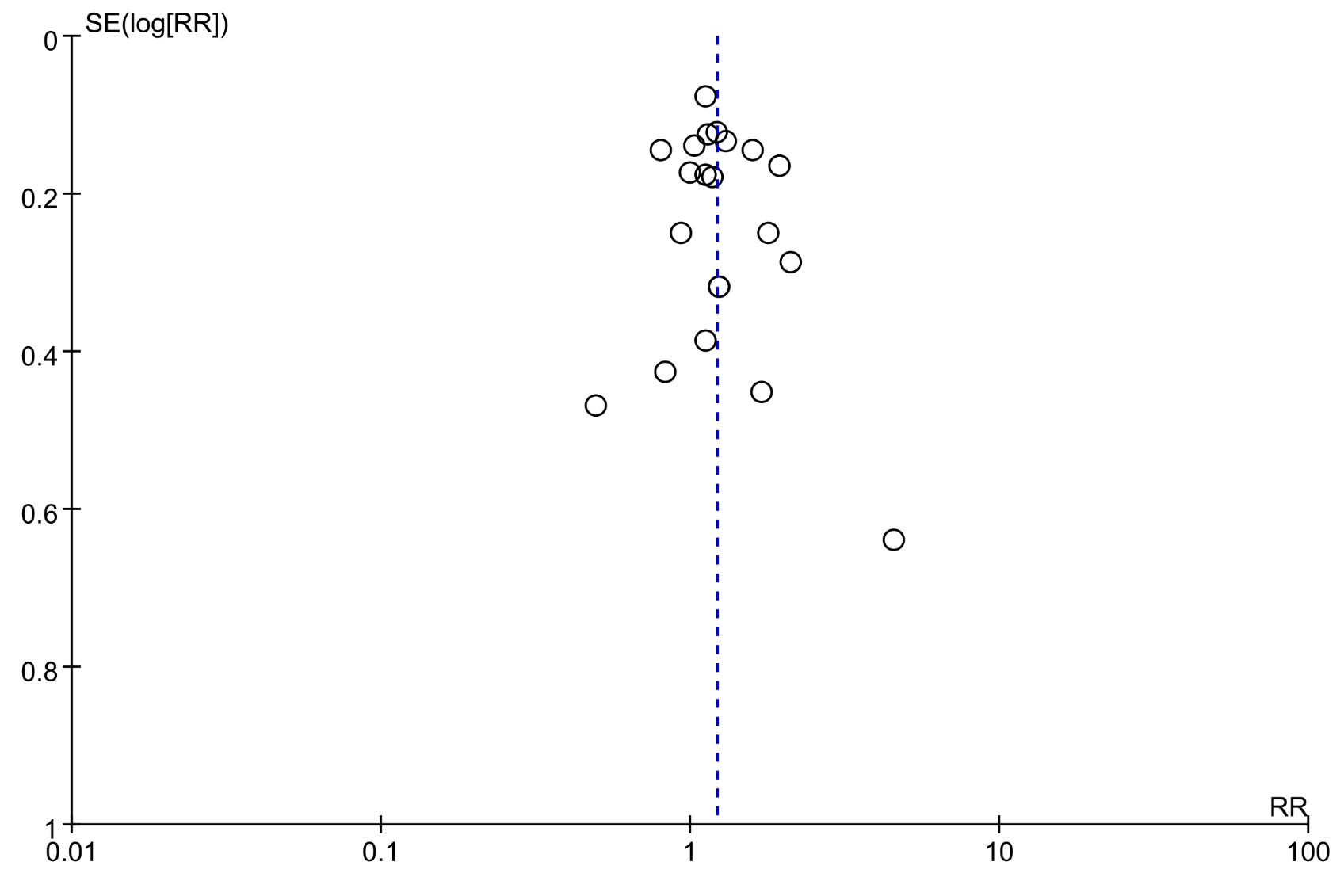


Figure S5 Funnel Plot of Red Meat Intake and Bladder Cancer Risk


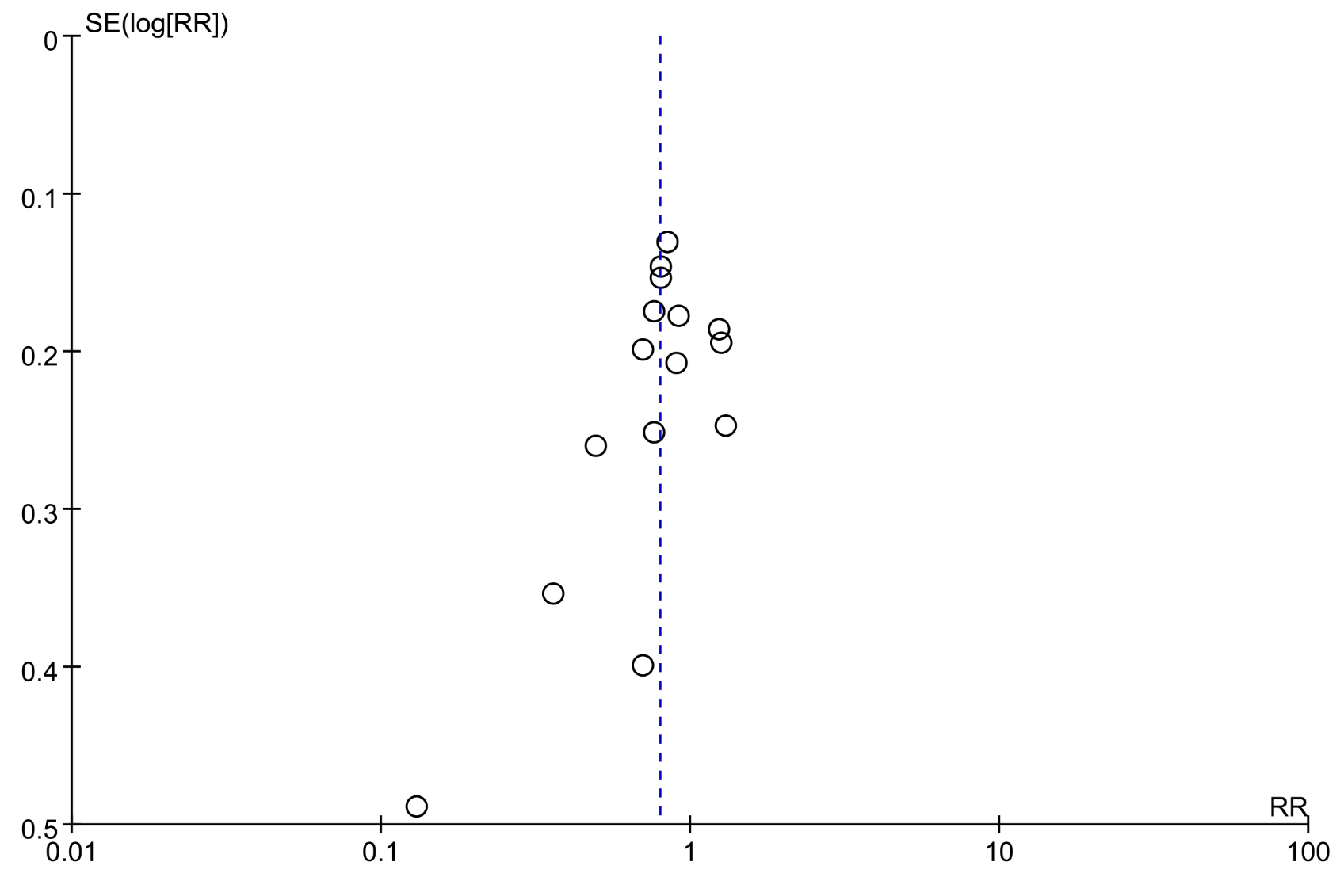


Figure S6 Funnel Plot of Fish Intake and Bladder Cancer Risk


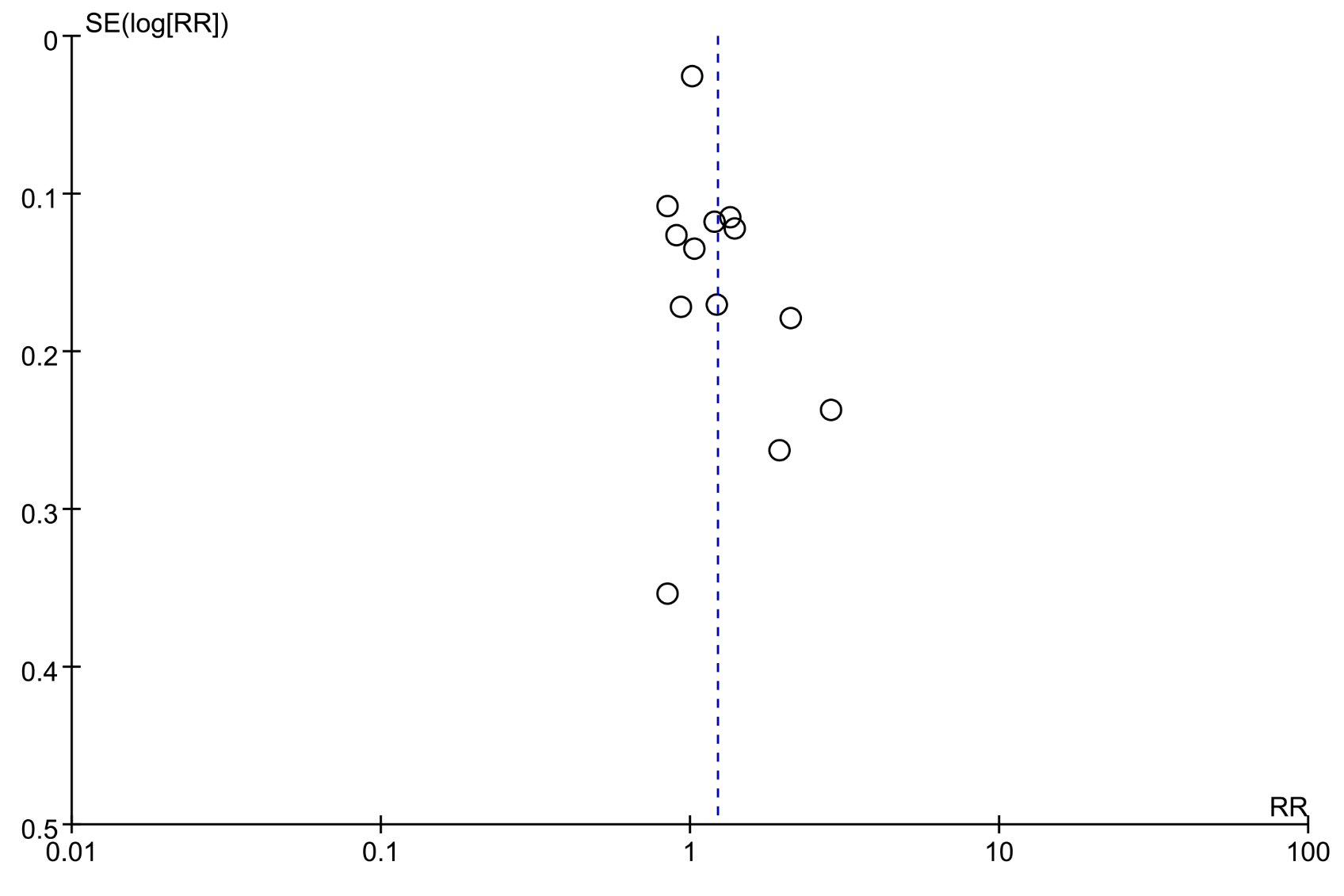


Figure S7 Funnel Plot of Processed Meat Consumption and Bladder Cancer Risk


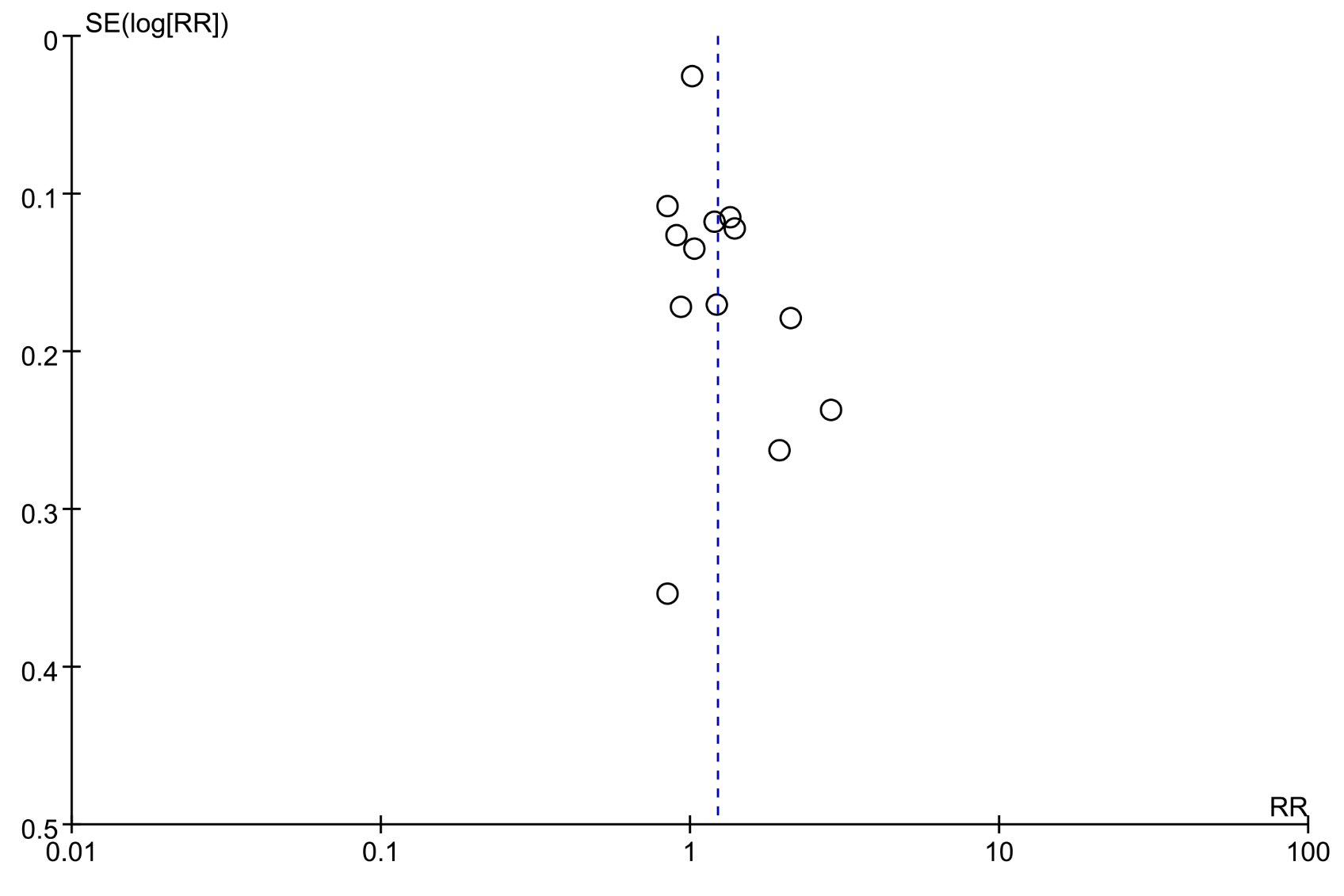


Figure S8 Funnel Plot of Red Meat Consumption (100 g Per Day Increment) and Bladder Cancer Risk


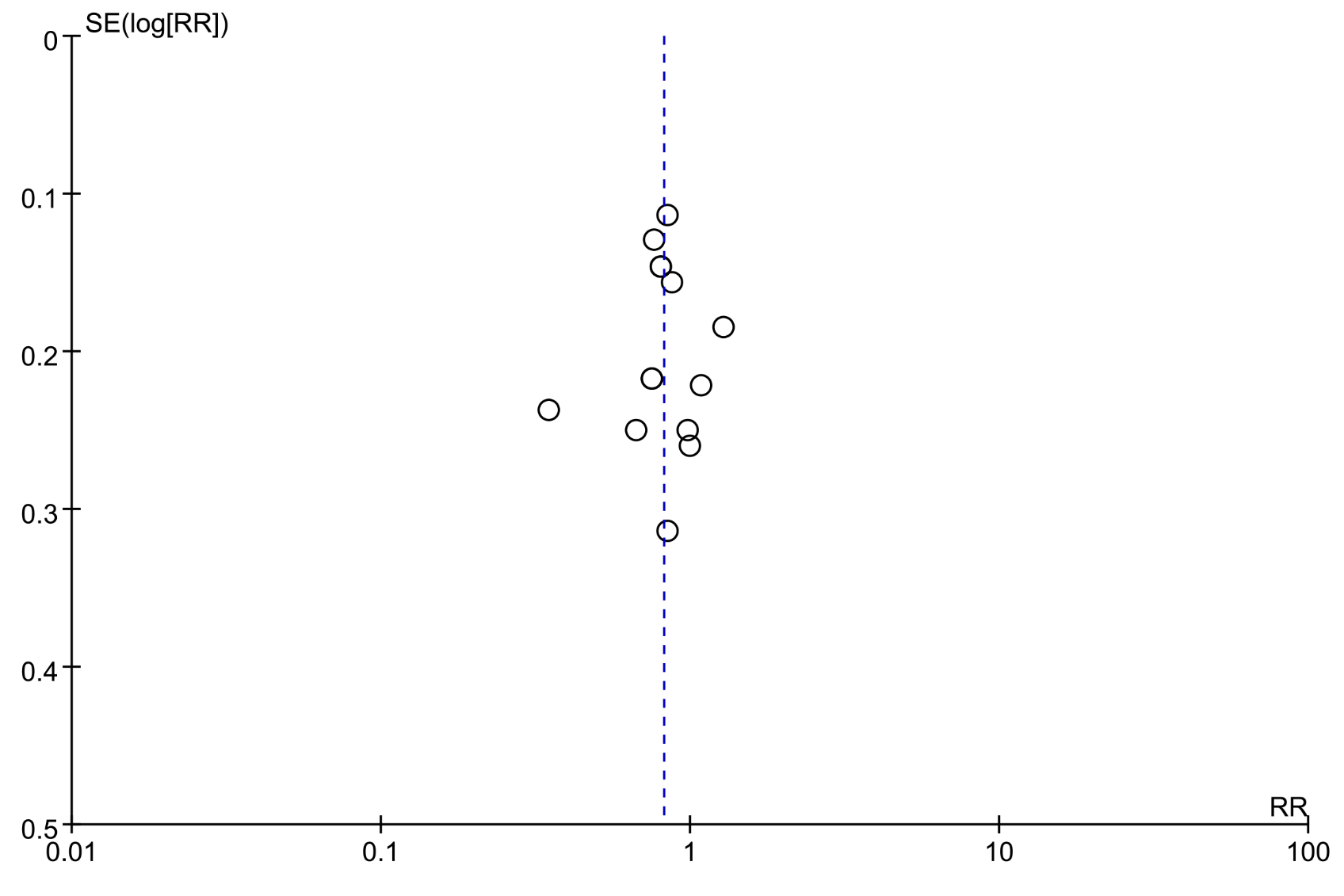


Figure S9 Funnel Plot of Total Fruit and Vegetable Intake and Bladder Cancer Risk


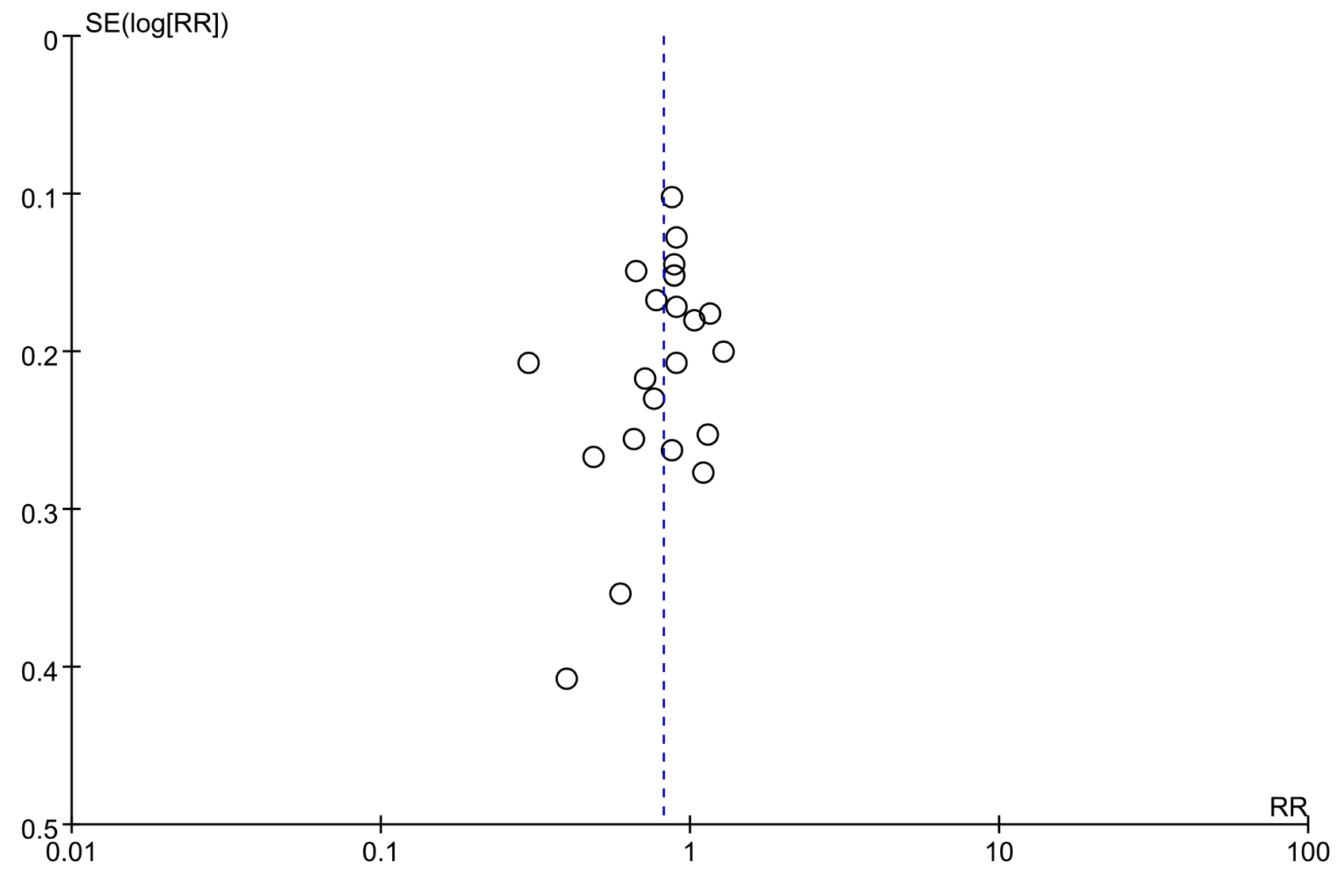


Figure S10 Funnel Plot of Total Vegetable Intake and Bladder Cancer Risk


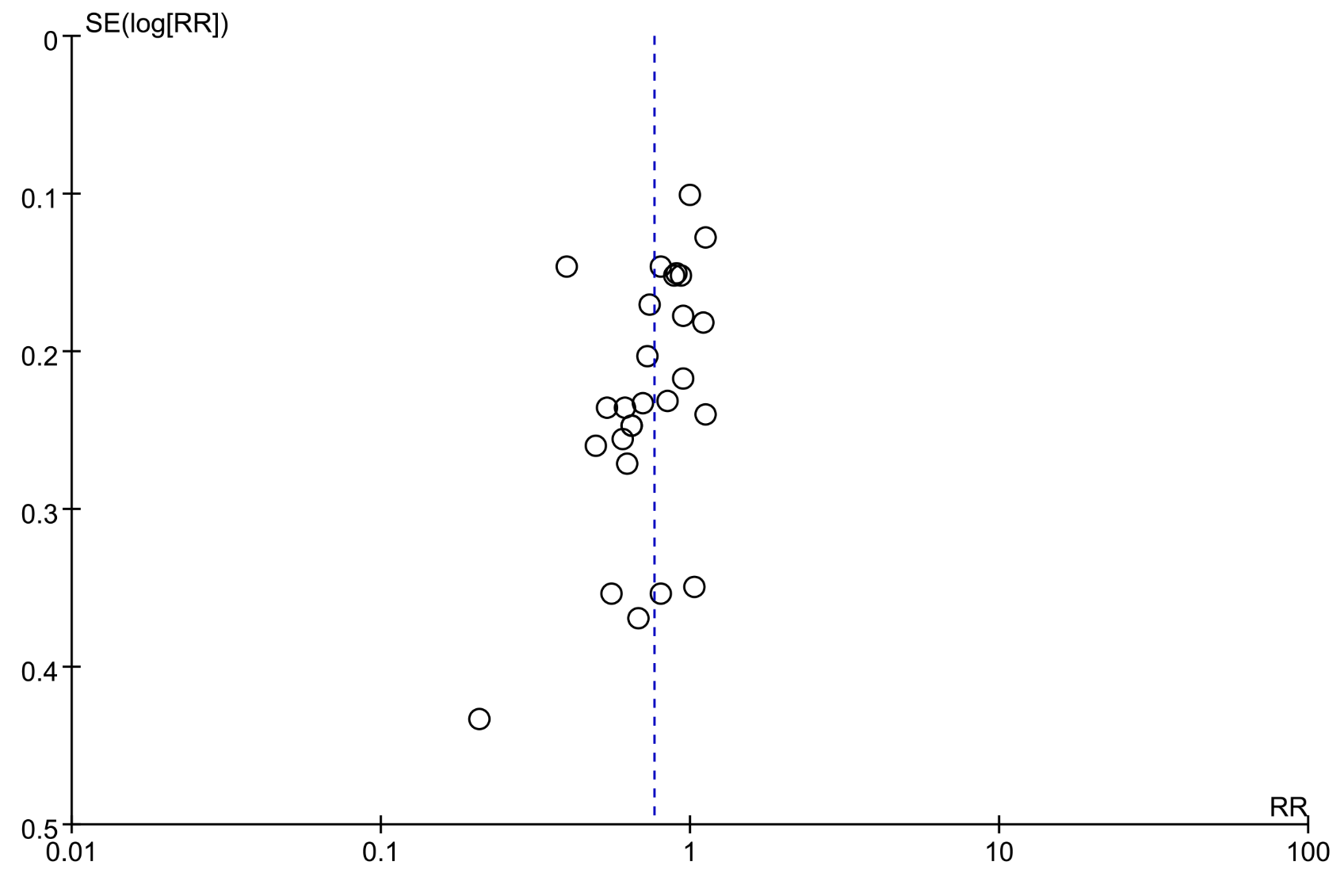


Figure S11 Funnel Plot of Total Fruit Intake and Bladder Cancer Risk


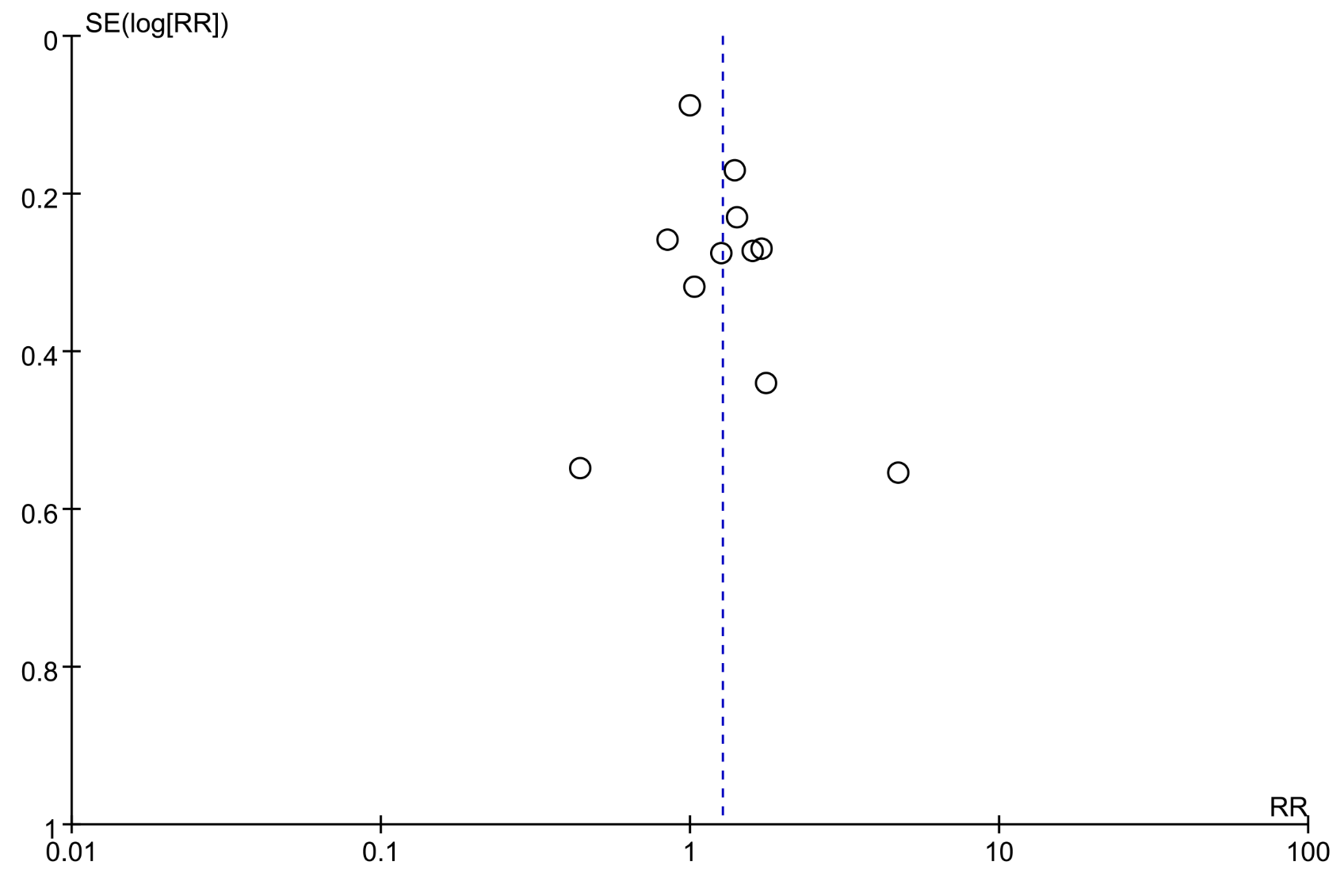


Figure S12 Funnel Plot of Dietary Fat Intake and Bladder Cancer Risk


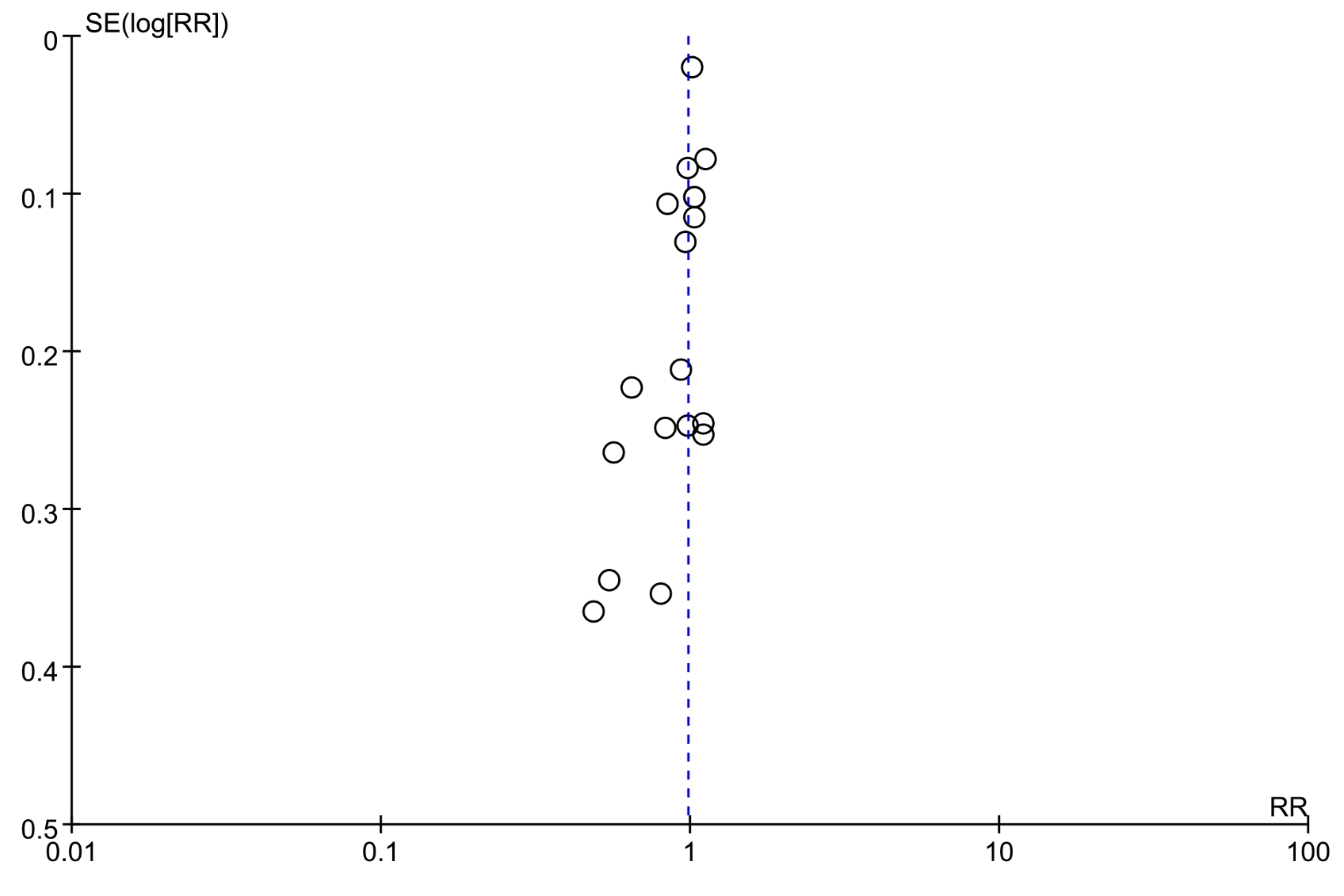


Figure S13 Funnel Plot of Milk Intake and Bladder Cancer Risk


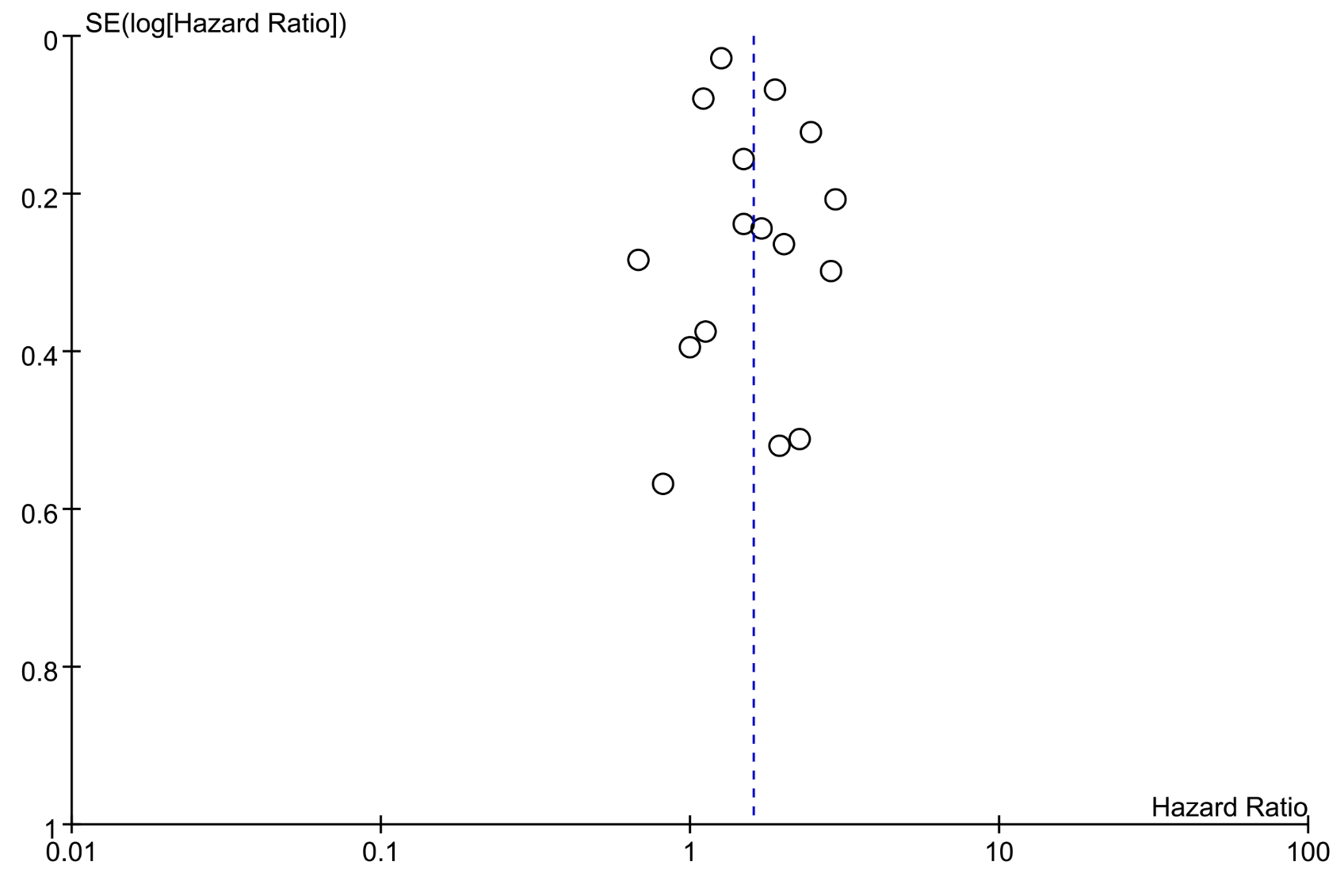


Figure S14 Funnel Plot of Radiotherapy for Prostate Cancer and Bladder Cancer Risk


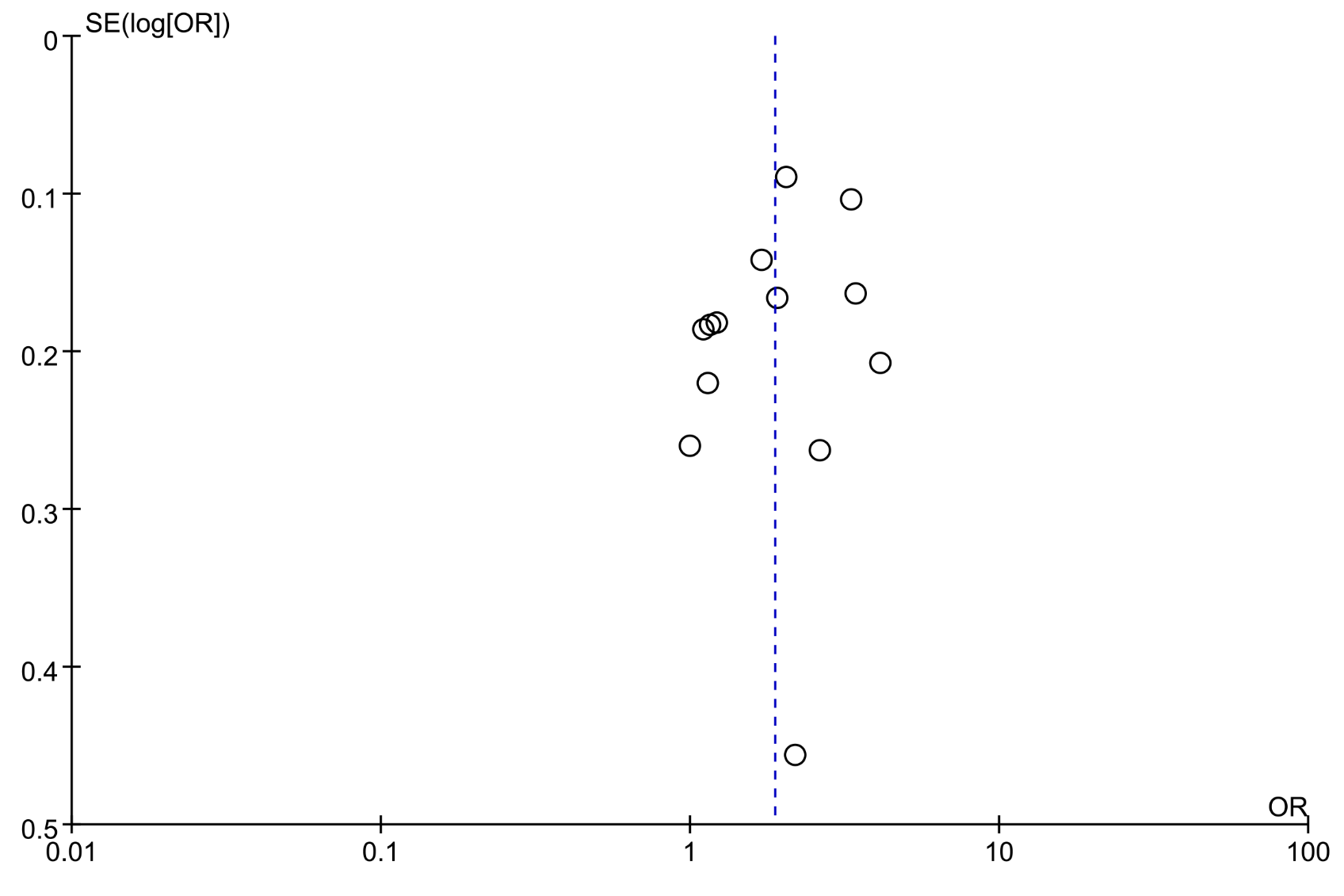


Figure S15 Funnel Plot of Urinary Calculi and Bladder Cancer Risk


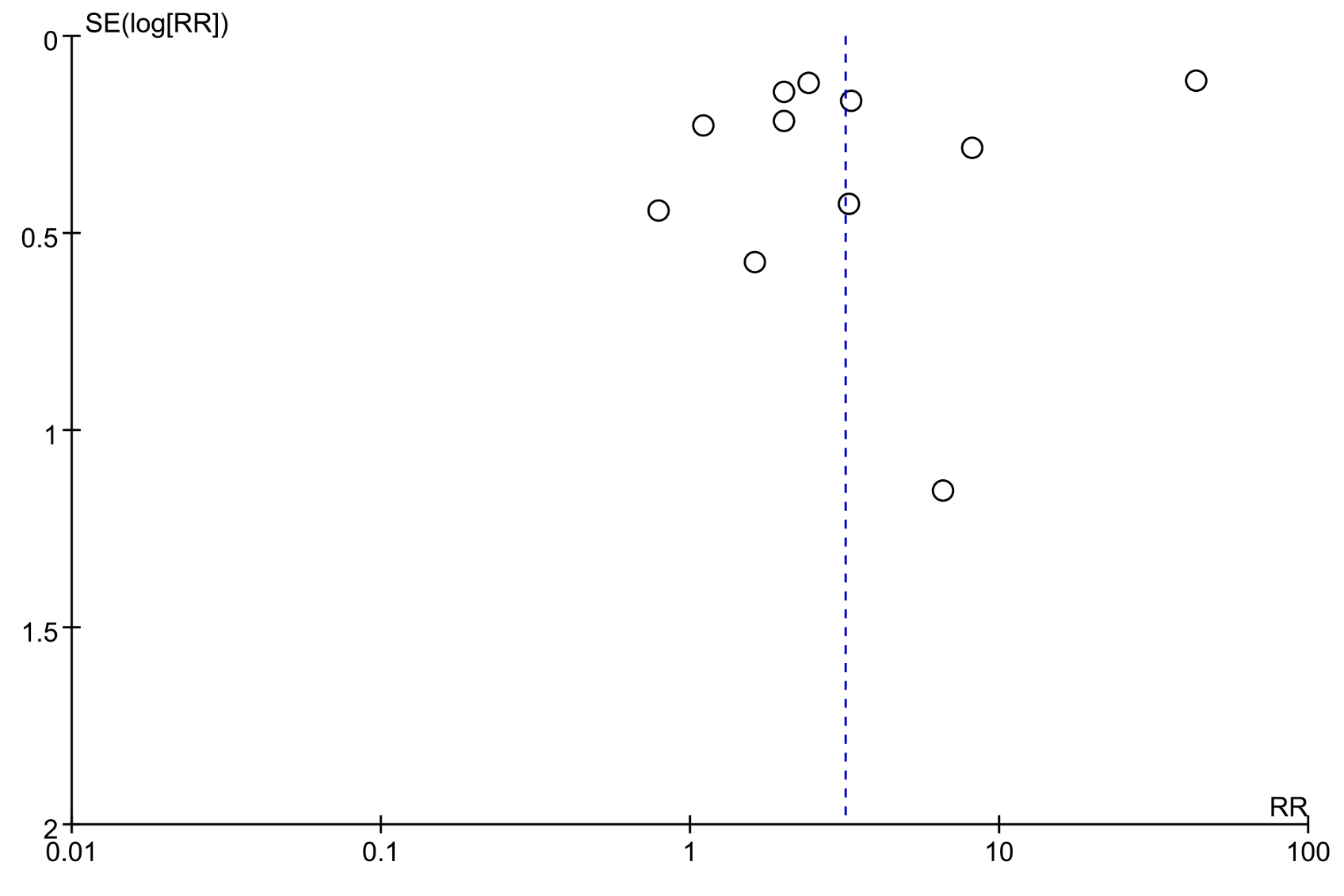


Figure S16 Funnel Plot of Renal Transplant and Bladder Cancer Risk


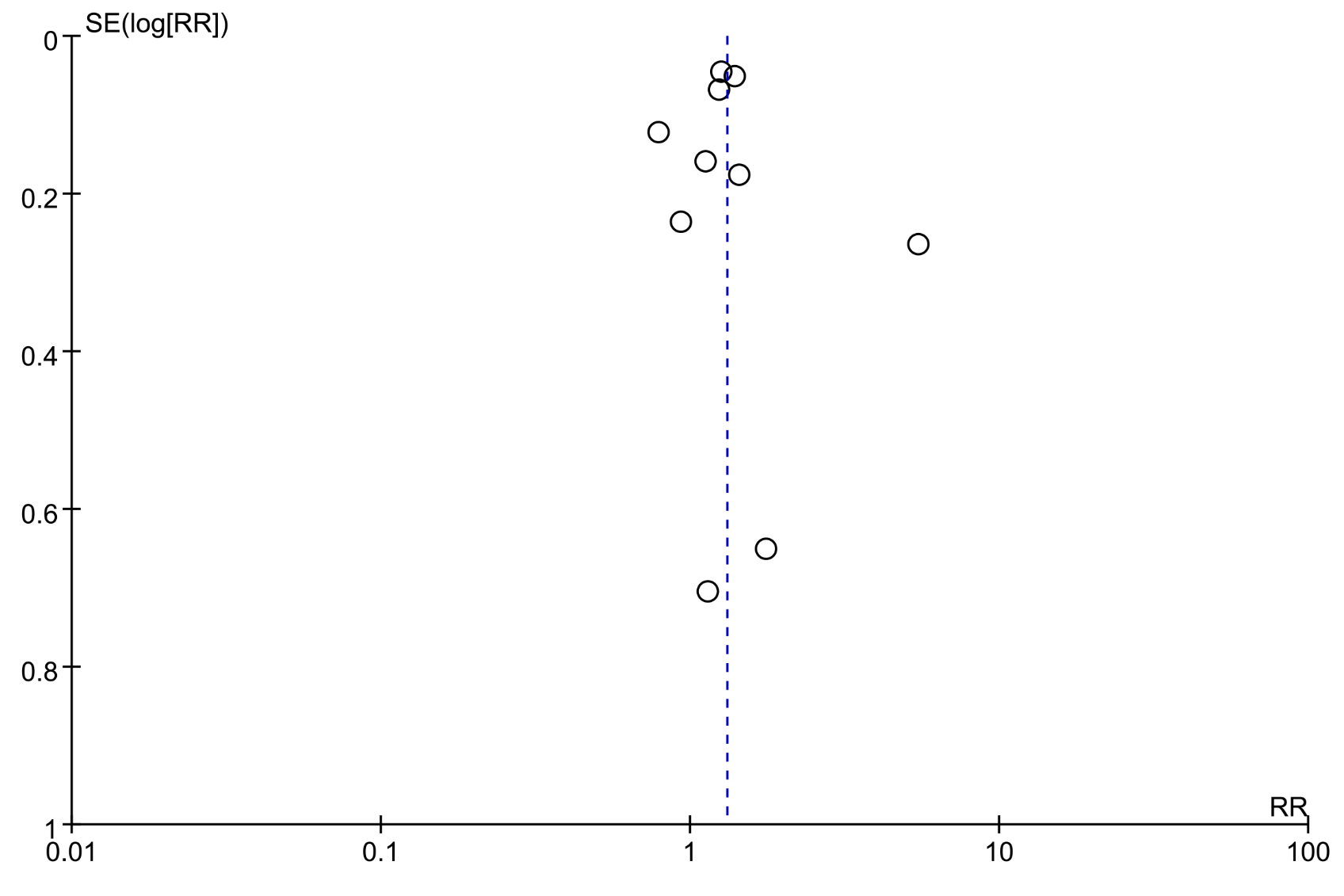


Figure S17 Funnel Plot of Overall Atopy and Bladder Cancer Risk


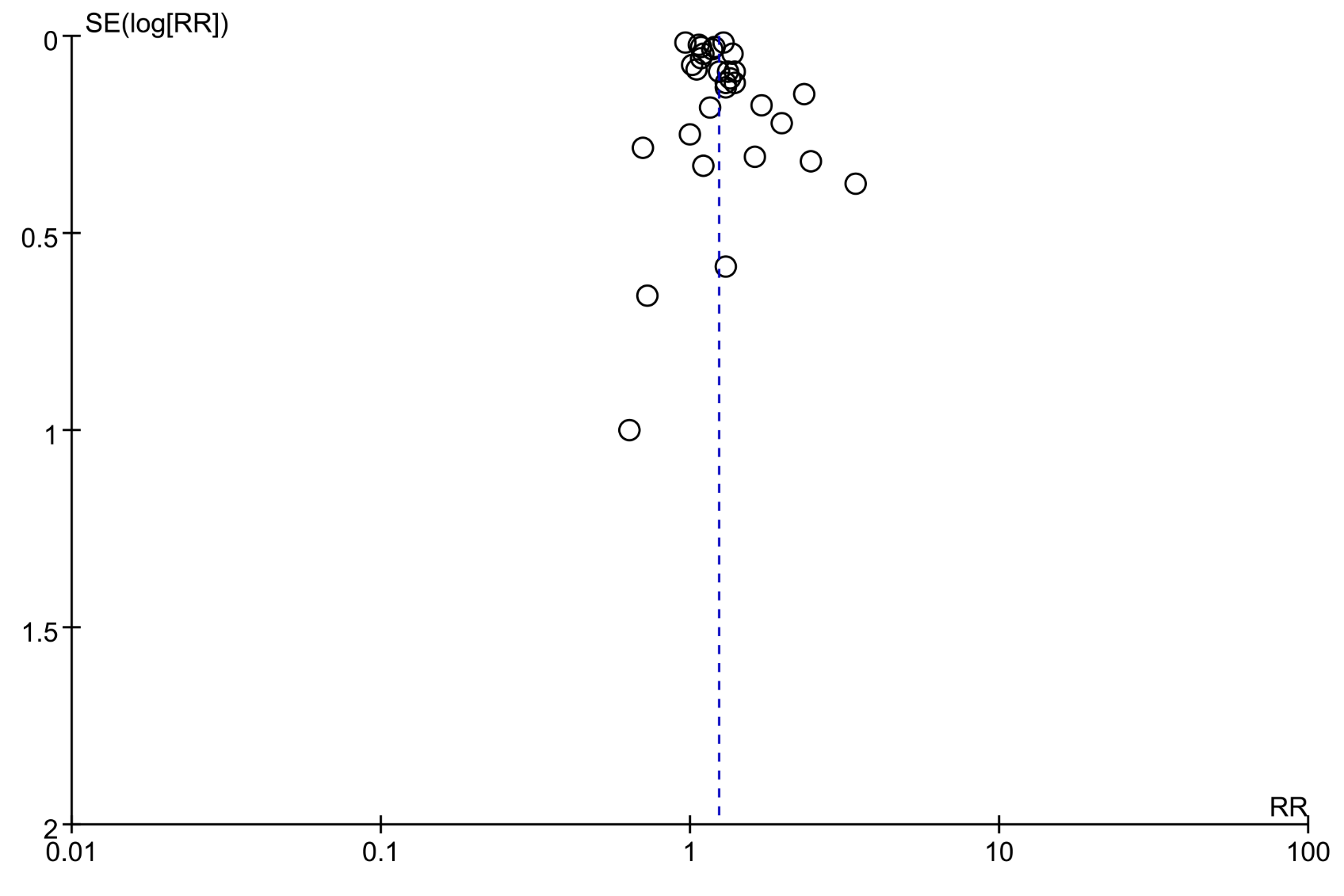


Figure S18 Funnel Plot of Diabetes and Bladder Cancer Risk


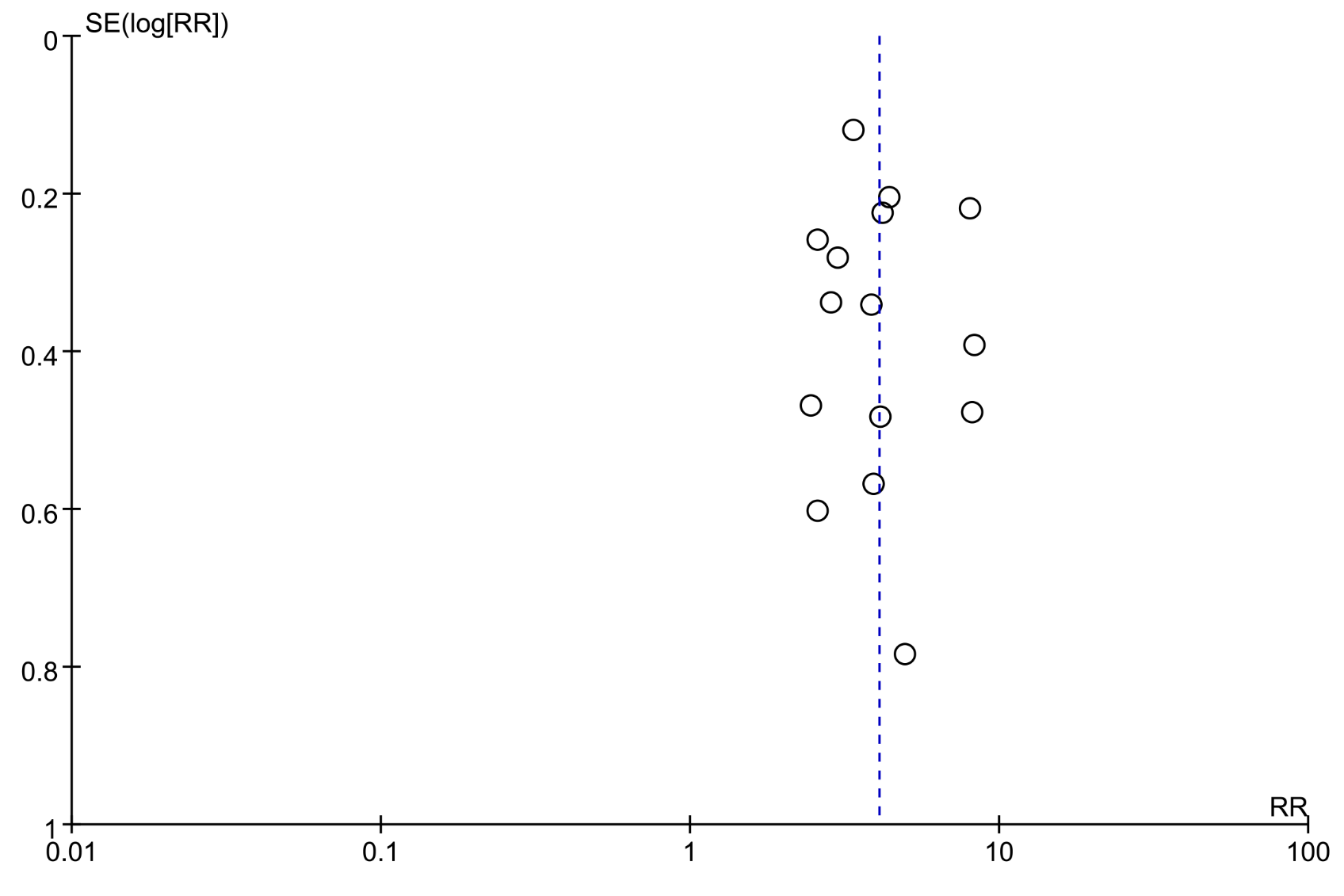


Figure S19 Funnel Plot of Opium Consumption and Bladder Cancer Risk


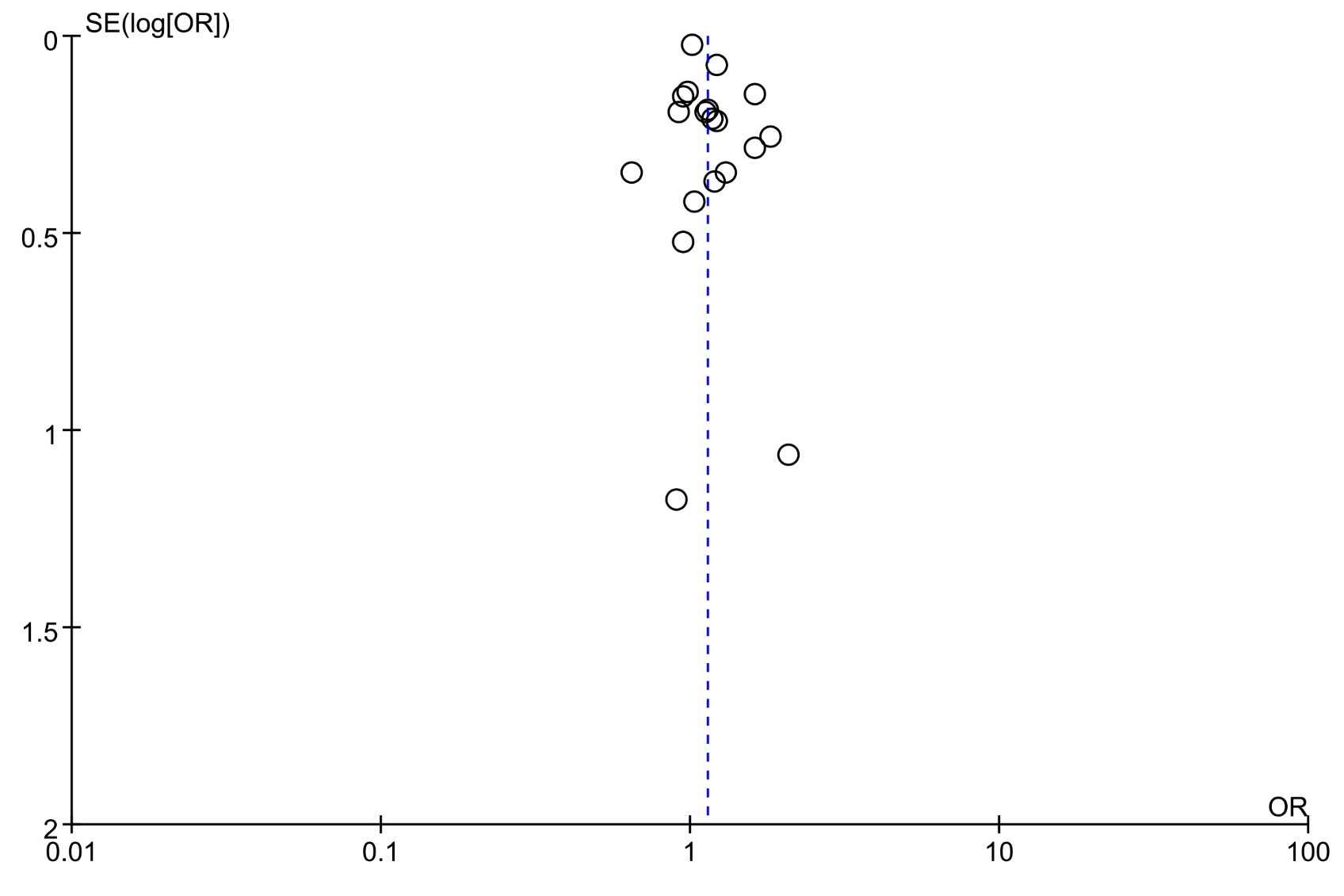


Figure S20 Funnel Plot of Pioglitazone and Bladder Cancer Risk


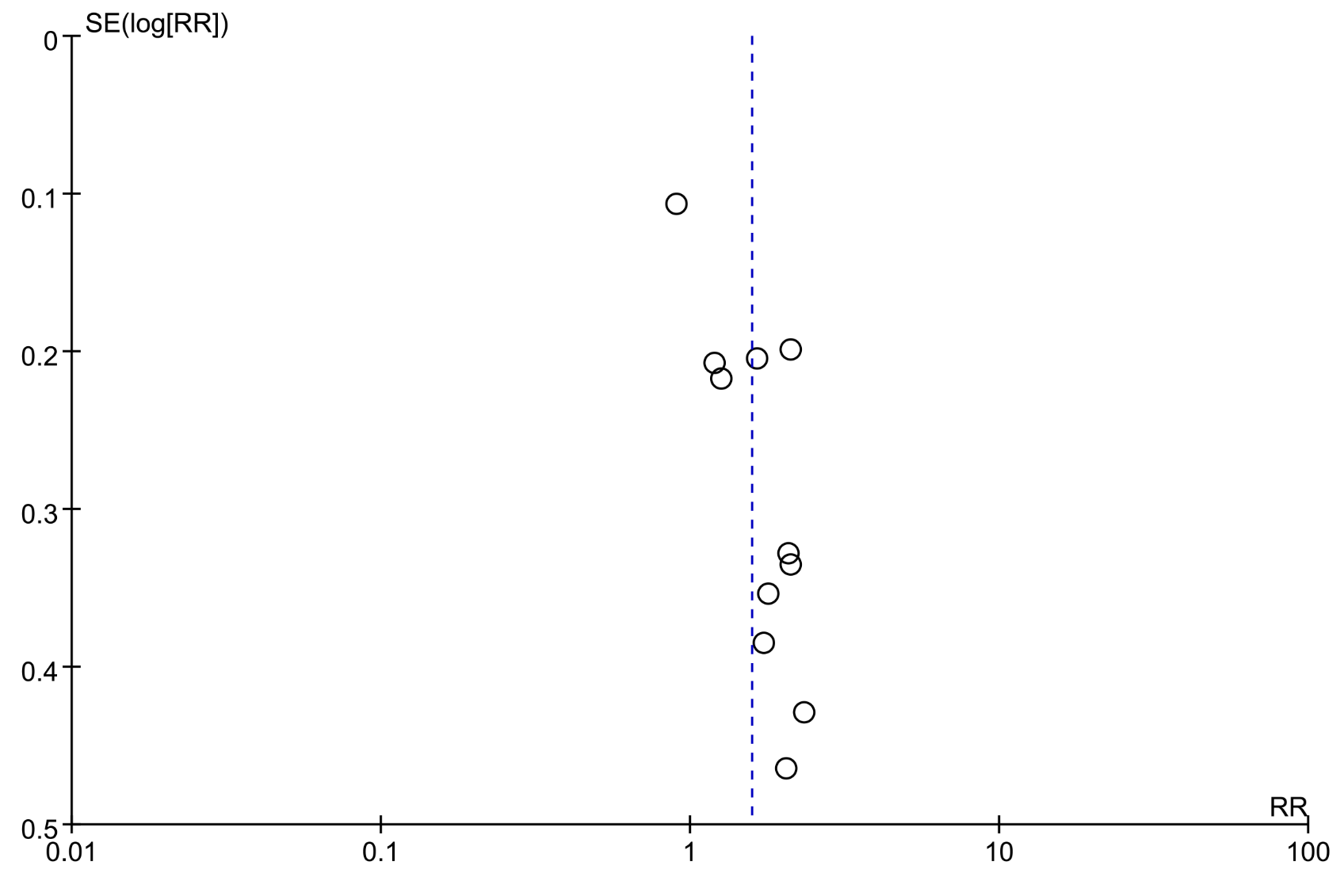


Figure S21 Funnel Plot of Disinfection By-products and Bladder Cancer Risk


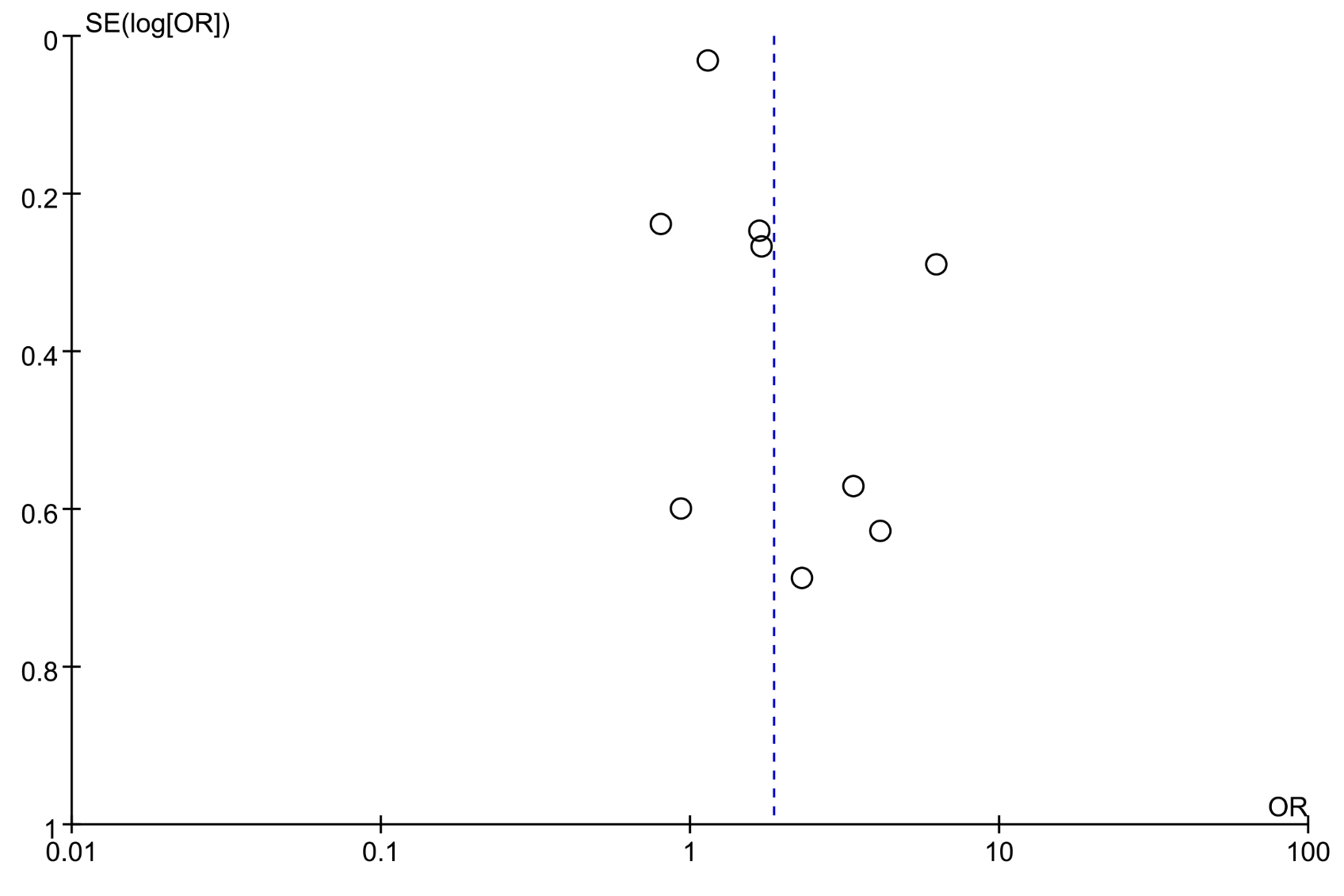


Figure S22 Funnel Plot of Pesticide Exposure and Bladder Cancer Risk


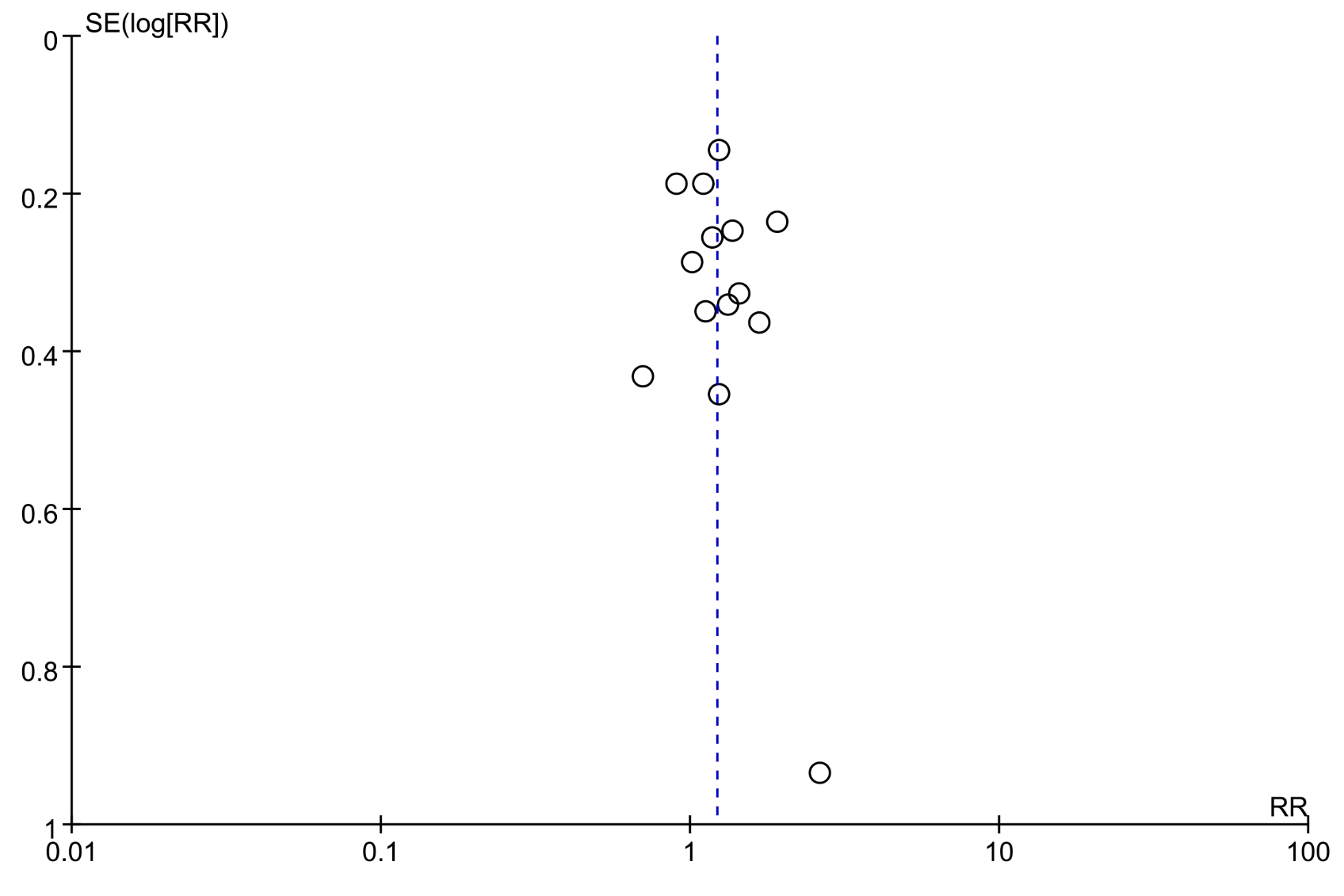


Figure S23 Funnel Plot of Secondhand Smoking and Bladder Cancer Risk


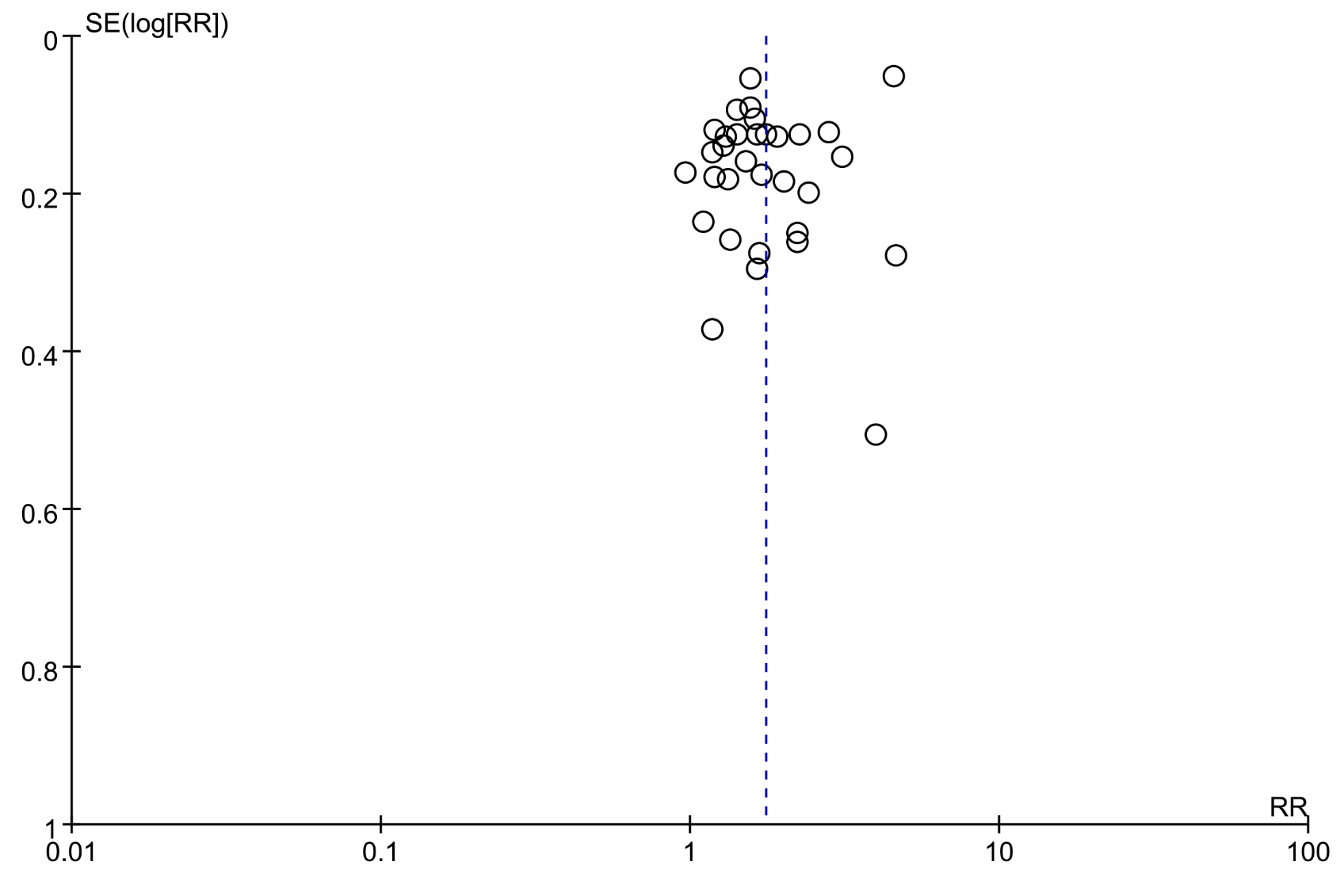


Figure S24 Funnel Plot of Smoking Intensity and Bladder Cancer Risk


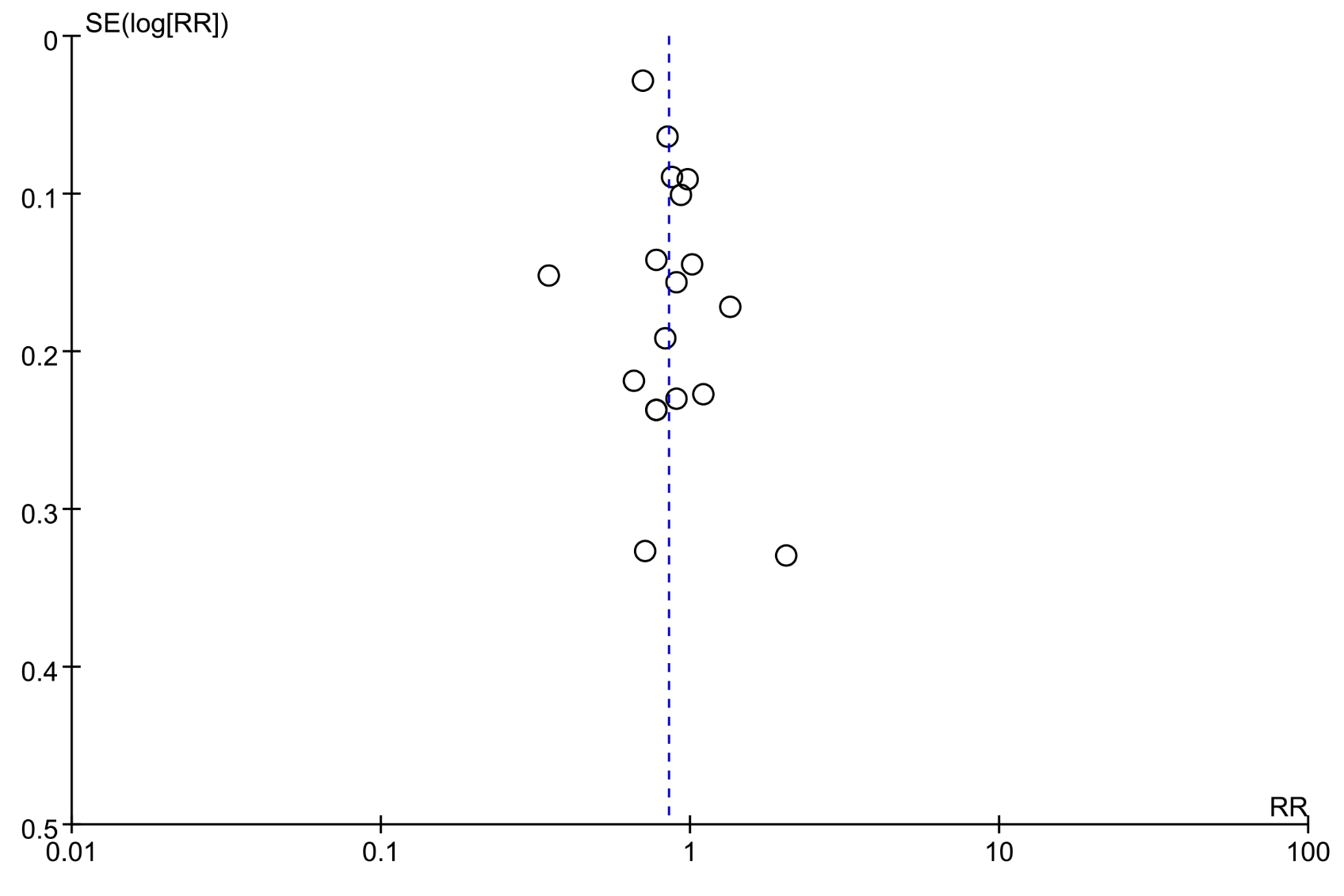


Figure S25 Funnel Plot of Physical Activity and Bladder Cancer Risk


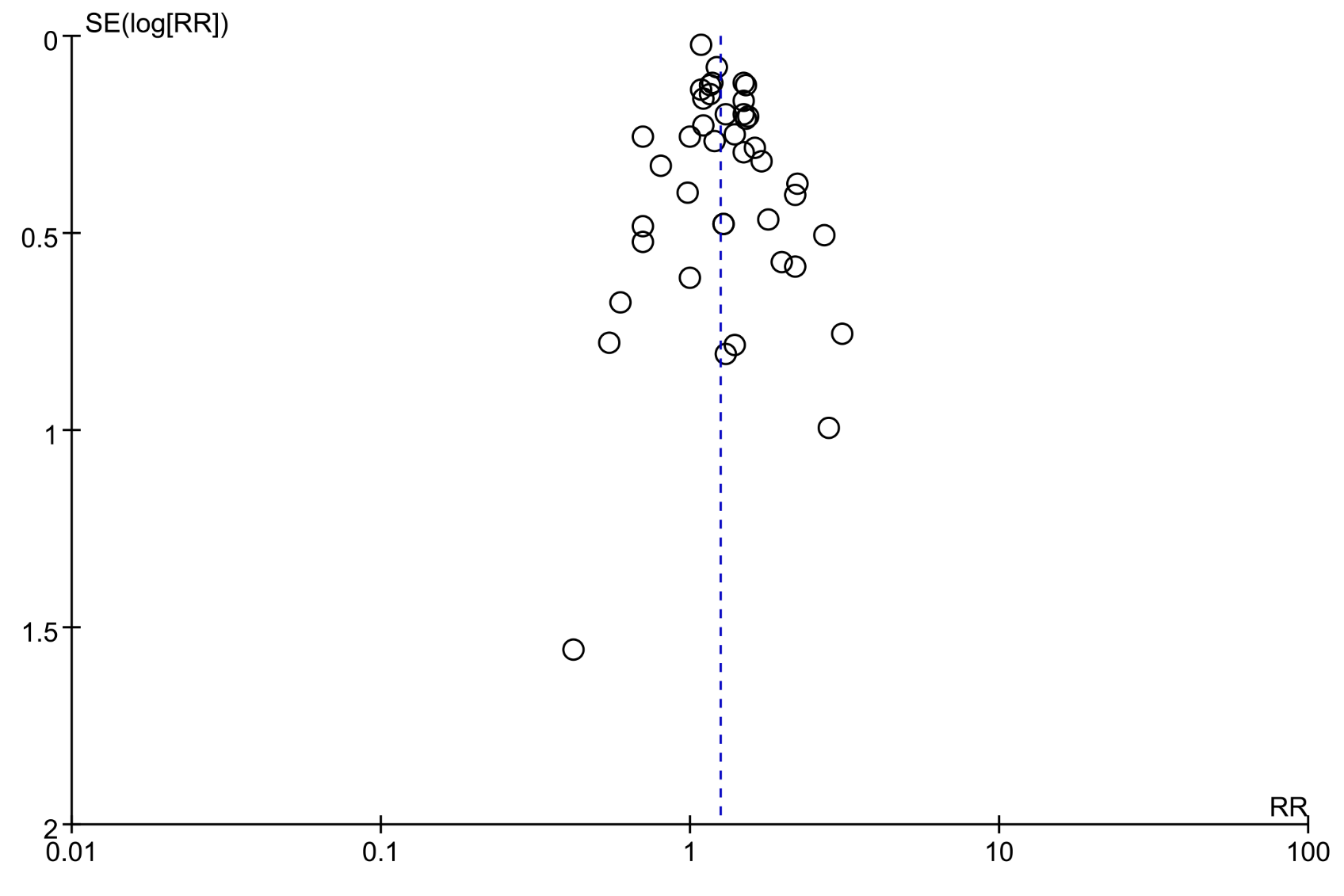


Figure S26 Funnel Plot of Painters and Bladder Cancer Risk


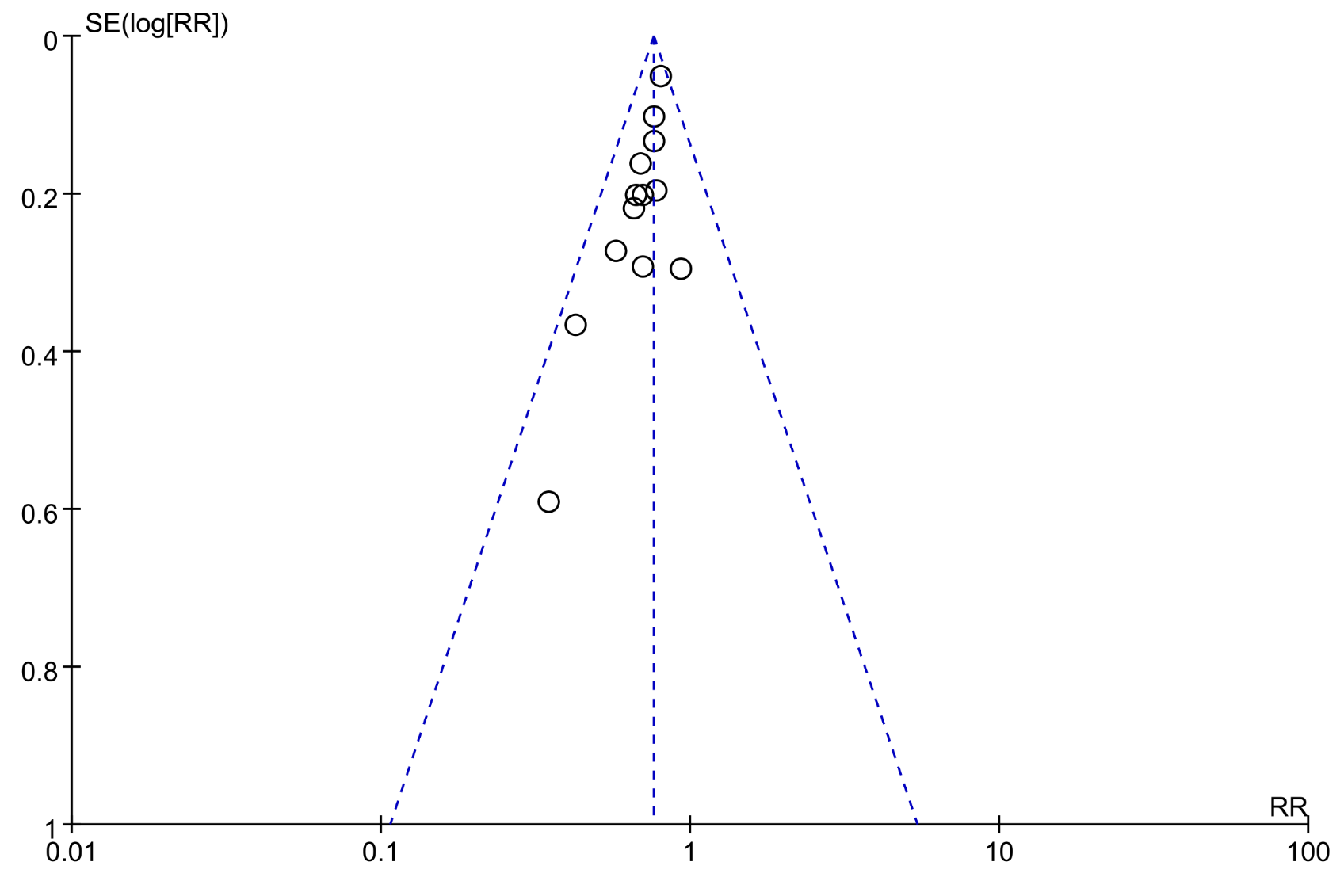


Figure S27 Funnel Plot of Parity and Bladder Cancer Risk


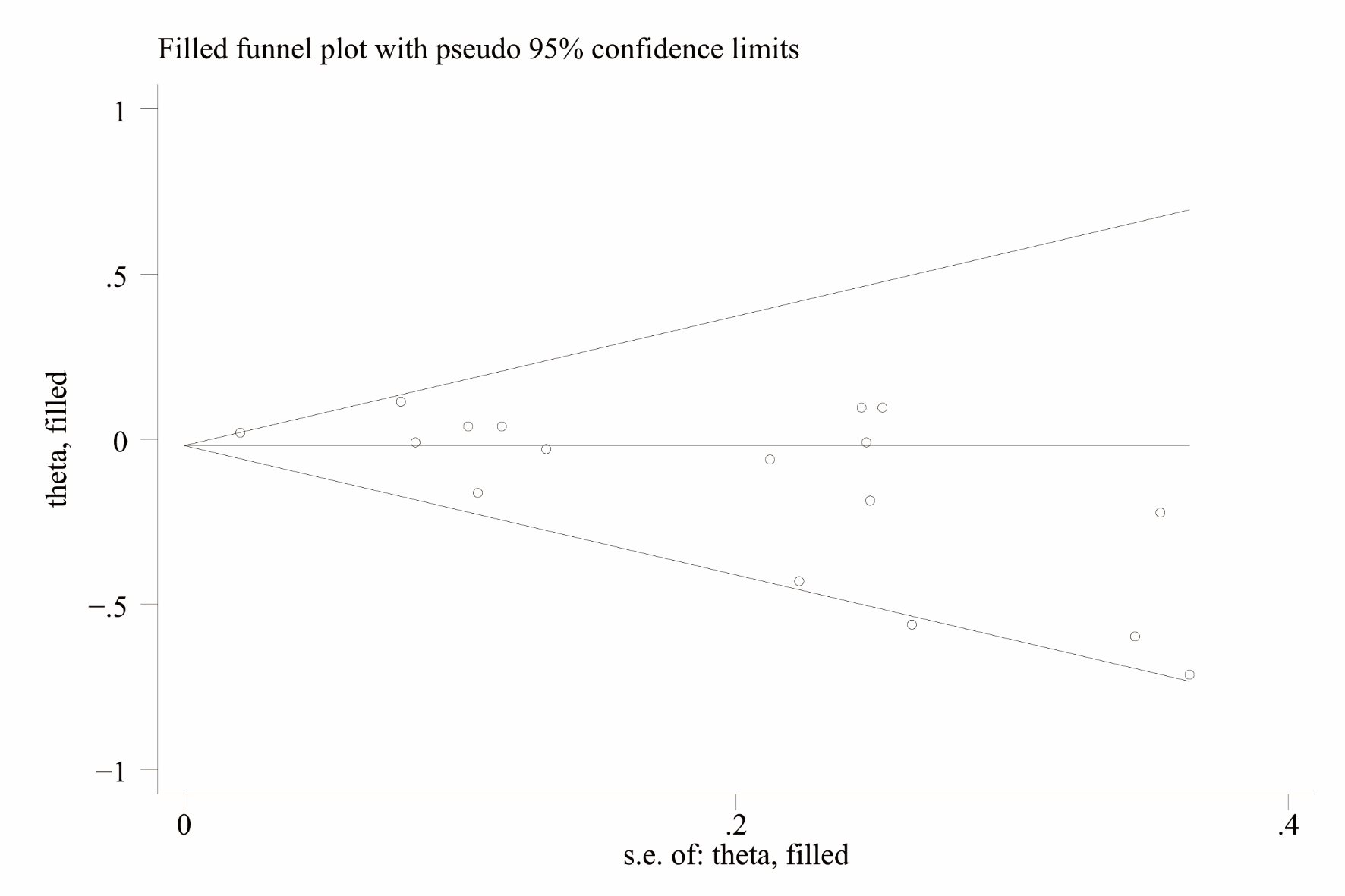


Figure S28. Trim-and-fill adjusted funnel plot for milk intake and bladder cancer risk


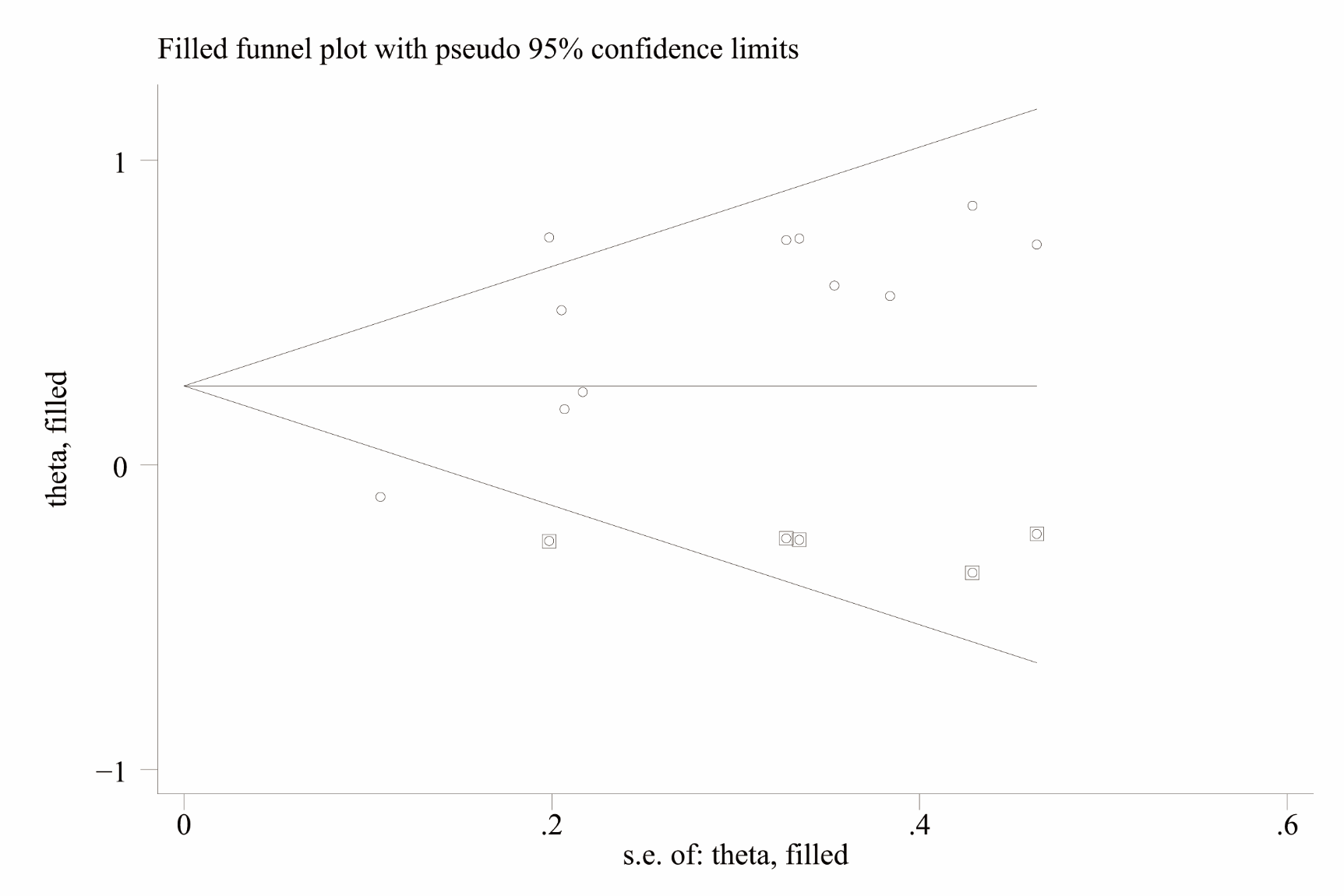


Figure S29. Trim-and-fill adjusted funnel plot for disinfection by-products exposure and bladder cancer risk


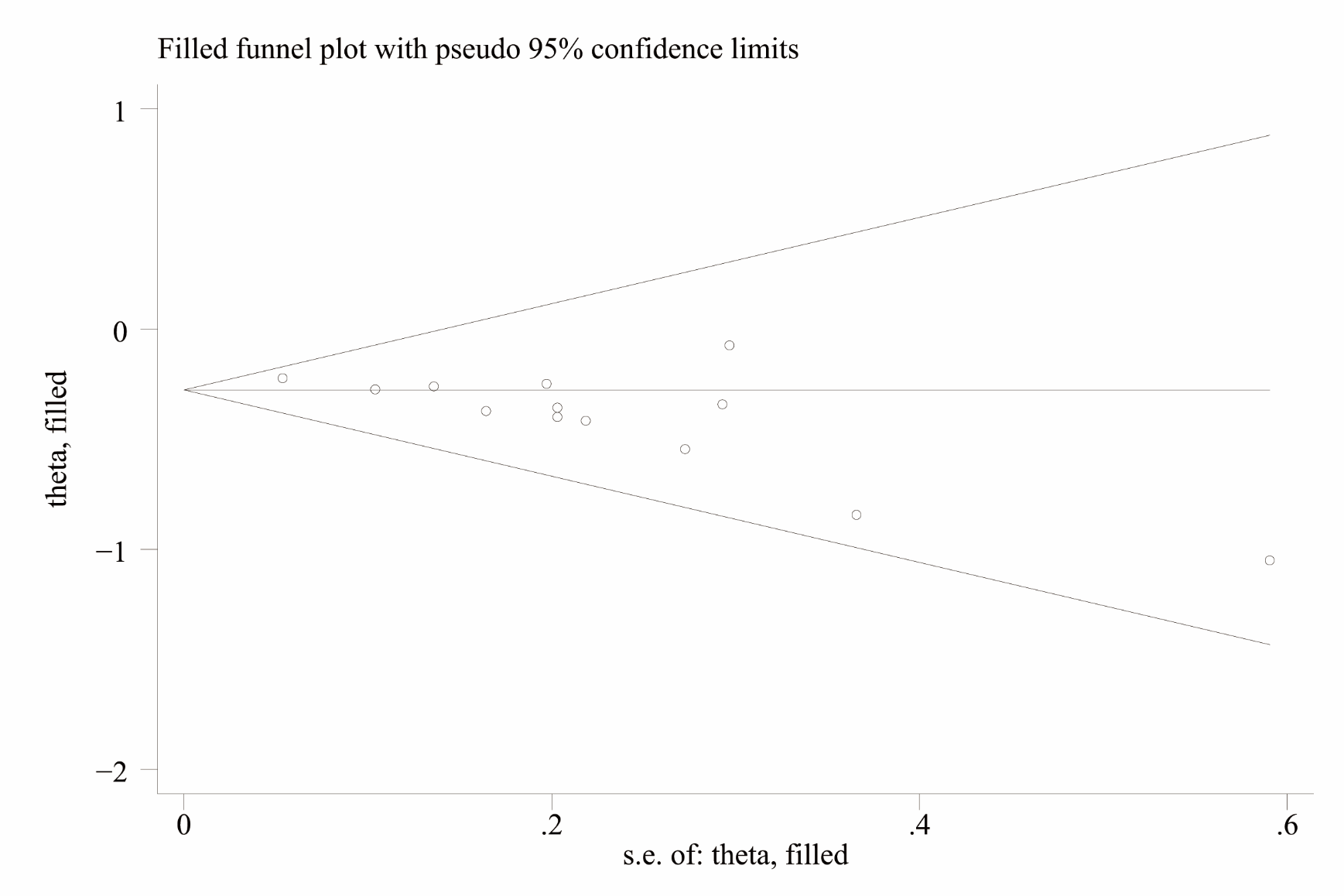


Figure S30. Trim-and-fill adjusted funnel plot for parity and bladder cancer risk
